# Gut bacteria of *Cuora amboinensis* (turtle) produce broad-spectrum antibacterial molecules

**DOI:** 10.1038/s41598-019-52738-w

**Published:** 2019-11-18

**Authors:** Noor Akbar, Naveed Ahmed Khan, K. Sagathevan, Mazhar Iqbal, Abdul Tawab, Ruqaiyyah Siddiqui

**Affiliations:** 1 Department of Biological Sciences, School of Science and Technology, Sunway University, Bandar Sunway, 47500 Petaling Jaya, Selangor Malaysia; 2 Department of Biology, Chemistry and Environmental Sciences, College of Arts and Sciences, American University of Sharjah, University City, Sharjah, 26666 United Arab Emirates; 3 Health Biotechnology Division, National Institute for Biotechnology and Genetic Engineering (NIBGE), Jhang Road, Faisalabad, 44000 Punjab Pakistan

**Keywords:** Applied microbiology, Drug development

## Abstract

Antimicrobial resistance is a major threat to human health, hence there is an urgent need to discover antibacterial molecule(s). Previously, we hypothesized that microbial gut flora of animals are a potential source of antibacterial molecules. Among various animals, *Cuora amboinensis* (turtle) represents an important reptile species living in diverse ecological environments and feed on organic waste and terrestrial organisms and have been used in folk medicine. The purpose of this study was to mine turtle’s gut bacteria for potential antibacterial molecule(s). Several bacteria were isolated from the turtle gut and their conditioned media were prepared. Conditioned media showed potent antibacterial activity against several Gram-positive (*Bacillus cereus*, *Streptococcus pyogenes* and methicillin-resistant *Staphylococcus aureus*) and Gram-negative (neuropathogenic *Escherichia coli* K1, *Serratia marcescens, Pseudomonas aeruginosa, Salmonella enterica* and *Klebsiella pneumoniae*) pathogenic bacteria. Conditioned media-mediated bactericidal activity was heat-resistant when treated at 95°C for 10 min. By measuring Lactate dehydrogenase release, the results showed that conditioned media had no effect on human cell viability. Tandem Mass Spectrometric analysis revealed the presence of various secondary metabolites, i.e., a series of known as well as novel *N-*acyl-homoserine lactones, several homologues of 4-hydroxy-2-alkylquinolines, and rhamnolipids, which are the signature metabolites of *Pseudomonas* species. These findings are significant and provide the basis for rational development of therapeutic interventions against bacterial infections.

## Introduction

Drug-resistant bacterial pathogens are posing a major threat to public health, contributing to more than 14 million deaths annually^1^. Among various bacteria, *Escherichia coli, Staphylococcus aureus*, *Pseudomonas aeruginosa*, *Mycobacterium* spp., *Listeria* spp., *Shigella* spp., *Campylobacter* spp., *Salmonella* spp., and *Klebsiella pneumoniae* are major cause of urinary tract infections, gastroenteritis, food poisoning, respiratory infections, meningitis, skin diseases etc.,^2–6^ while *Enterococcus faecium, K. pneumoniae, Enterobacter* species*, Acinetobacter baumanii, S. aureus*, and *P. aeruginosa* are frequently attributed to nosocomial infections^7^. The ability of pathogenic bacteria to acquire resistance to multiple drugs has exacerbated the situation further^8,9^. The rate at which bacterial pathogens are developing drug resistance combined with limited success in the introduction of new antibacterials in clinical practice highlights the need to identify new antibacterial molecules from unique sources^10,11^.

Microorganisms compete in their natural habitats for food and space^12^. They synthesize and secrete bioactive molecules to kill the competitors. The producer species are immune to these secreted compounds^12^. For example, *Staphylococcus lugdunensis* isolated from human nasal cavities produce secondary metabolite exhibited broad-spectrum antibacterial activities^13^. Gut bacteria produce several compounds such as phenolic compounds and other acids that can inhibit the growth of other bacteria^14^. Similarly, they produce different peptides and non-peptidic molecules that showed promising antibacterial properties^14^. Bacteria isolated from the gut of honeybee produced phenazine like compounds with robust antibacterial activities^15^. Our recent research revealed that cockroach gut bacteria produced broad spectrum antibacterial molecules^12^. We have hypothesized earlier that gut bacteria of animals/pests represent a potentially unique source to mine for potential antibacterial molecules. In support, our recent studies showed that gut bacteria of cockroaches showed potent and broad-spectrum antibacterial activities against Gram-positive and Gram-positive bacteria^12^. *Cuora amboinensis* (turtle) represents an important reptile species living in diverse ecological environments such as in the storm drains of Brunei and feed on *Chironomidae pupae* and larvae, organic waste and terrestrial organisms such as snails, cockroach and carrion^16^. These are seriously polluted environments, and yet these turtles thrive in these habitat. More importantly, *C. amboinensis* has been hunted for use in traditional medicine^17,18^. In this study, we aim to mine turtle’s gut bacteria for potential antibacterial molecule(s). Several bacteria were isolated from the turtle gut, these bacteria were cultured in minimal media and their conditioned media (CM) were prepared. The CM were tested for their antibacterial activity against selected Gram-positive and Gram-negative pathogenic bacteria. The CM with potent antibacterial properties were selected for further study. The selected bacteria were identified by microbiological, biochemical and molecular methods. Moreover, among all four CM tested, CM3 (*P. aeruginosa*) showed most promising broad-spectrum antibacterial activities. CM3 was further subjected to detailed characterization of the secondary metabolites. The results from spectrometry analysis revealed the production of several known and novel *N-*acyl homoserine lactone (AHLs), 4-hydroxy-2-alkylquinolines (HAQs) and numerous rhamnolipids molecules with potent antibacterial properties. The aim of this study was to mine turtle’s gut bacteria to identify the potential antibacterial molecule(s).

## Materials and Methods

### Dissection of turtle and identification of bacteria

The use of animal was approved by the Sunway University Research Ethics Committee, SUNREC 2017/040. Furthermore, we also confirmed that all experimentations were performed in accordance with applicable regulations and guidelines as previously described^19^. All dissecting and surgical instruments were sterilized prior to experiments as well as surface sterilized using 70% ethanol throughout the dissection. Turtle was dissected and the entire gut was removed aseptically. Next, the bacteria were isolated from the turtle gut using sterile cotton swabs, plated on blood agar plates and incubated at 37 °C for 24 h. Numerous bacterial species were observed based on appearance, shape, texture and colour on blood agar plates. Selected colonies were grown on nutrient agar plates for 24 h at 37°C. Next, bacteria were identified using microbiological, Analytical Profile Index (API) and biochemical tests^12^. The bacteria were further identified using universal primers (27 F 5′ AGAGTTTGATCMTGGCTCAG 3′ and 1492 R 5′ TACGGYTACCTTGTTACGACTT 3′ to amplify 16 S rRNA gene^20^. Next, PCR product were sequenced commercially (1^st^ Base; Axil Scientific Pt. Ltd. Singapore). The resulted sequences were exported into alignment tool “Basic Local Alignment Search Tool (BLAST) to identify matches with existing reference sequences. The phylogenetic tree was reconstructed using the Maximum Likelihood (ML) method (MEGA 7.) based on GTR + G model with concatenated 16 S rRNA sequences.

### Preparation of bacterial conditioned medium

For conditioned media (CM) preparation, single bacterial colonies were grown in 200 mL of Roswell Park Memorial Institute (RPMI) (minimal medium) for 24 h aerobically at 37 °C. After this incubation, the overnight bacterial cultures were centrifuged at 10,000 × g for 1 h at 4 °C. Finally, the supernatants were collected, filter-sterilized using 0.22 µm pore size filter and stored at −80 °C till further use for biological assays.

### Bacterial cultures

Bacterial species used in this study include methicillin resistant *S. aureus* (MRSA), neuropathogenic *E. coli* K1, *E. coli* K-12, *P. aeruginosa*, *S. enterica, S. marcescens, K. pneumoniae, B. cereus* and *S. pyogenes* (Table 1). Neuropathogenic *E. coli* K1 (018: K1:H7), strain E44 and MRSA were originally isolated from the cerebrospinal fluid (CSF) and blood cultures of a meningitis patient and of sepsis patients respectively (obtained from the Luton & Dunstable NHS Foundation Trust, Luton, England, UK). All other strains are isolated from clinical samples including *S. enterica, P. aeruginosa, S. marcescens, K. pneumoniae, B. cereus* and *S. pyogenes* (Table 1). Bacteria were cultured in nutrient broth for overnight at 37 °C aerobically prior to experiments as previously described^12,21^.

**Table 1:** Bacteria used in this study.

Bacteria	Strain
Methicillin-resistant *Staphylococcus aureus*	MTCC 381123 (clinical isolate)
*Streptococcus pyogenes*	ATCC 49399 (clinical isolate)
*Bacillus cereus*	MTCC 131621 (clinical isolate)
*Escherichia coli* K1	MTCC 710859 (clinical isolate)
*Salmonella enterica*	ATTC 14028 (clinical isolate)
*Serratia marcescens*	MTTC 13880 (clinical isolate)
*Pseudomonas aeruginosa*	ATCC 10145 (clinical isolate)
*Klebsiella pneumoniae*	ATCC 13883 (clinical isolate)
*Escherichia coli* K-12	MTCC 817356 (non-clinical isolate)

### Evaluation of bacterial supernatants for antibacterial assays

Antibacterial assays were performed as described previously^12,21^. Briefly, 1 × 10^6^ bacterial cells were incubated with 100 µL of CM for 2 h at 37 °C. Next, cultures were serially diluted and plated onto nutrient agar plates for 24 h at 37°C and bacterial c.f.u. were enumerated the following day. For negative controls, bacteria were incubated in PBS and *E. coli* K-12 CM while for positive control, bacteria were incubated with gentamicin (100 µg/mL). In some experiments, heat-inactivation of CM was performed for 10 min at 95°C prior to antibacterial assays^12,22^.

### *In vitro* cytotoxicity assays

Host cell cytotoxicity assays were performed to determine the effects of CM on human keratinocytes cells (HaCaT) as described earlier^23,24^. Briefly, HaCaT cells monolayers were grown in a 96 well plates and cells were incubated with 100 µL of CM for 24 h at 37°C with 5% CO_2_. Following this, supernatants were collected and cytotoxicity was determined using lactate dehydrogenase (LDH) kit (Cytotoxicity Detection kit). Percent cytotoxicity was determined by the estimation of LDH released from HaCaT cells as follows: cytotoxicity (%) = (sample value – negative control value) / (positive control value – negative control value) × 100. HaCaT cells monolayer grown in RPMI alone and incubated with Triton X-100 (0.1%) were taken as negative and positive controls respectively.

### MTT assay

MTT assay was performed as previously described^25^. HaCaT cell were grown up to 80-90% confluency in a 96 well plate at 37°C for 24 h in the presence of 5% CO_2_ and 95% humidity. After this incubation, CM (25, 50, 75 and 100 µL) were added to each well and incubated for 24 h at 37°C with 5% CO_2._ Next, 10 μL of freshly prepared MTT dye solution was added and incubated for 4 h and subsequently added 100 μL of DMSO to dissolve formazan crystals formed by live cells which reduces MTT. DMSO added to HaCaT cells monolayer grown in RPMI was taken as negative control. The absorbance was recorded at 540 nm and % inhibition was calculated using following formula; % Viability = Mean OD of test sample/Mean OD of negative control x 100. CC_50_ and MNTD (CC_90_) values were determined using GraphPad Prism version 8.0.2 (GraphPad Software, San Diego, CA, USA) software.

### Metabolic profiling of *P. aeruginosa* (CM3) supernatant using Mass Spectrometric analysis

To determine the identity of bioactive molecules, CM3 were subjected to LCMS/MS as described earlier^26,27^. Briefly, *P. aeruginosa* (CM3) was grown in RPMI medium (200 mL) and incubated at 37°C for 24 h aerobically. Next, the bacterial culture was centrifuged at 10,000 × g for 1 h at 4°C and the supernatant collected for further extraction using chloroform at (1:3) ratio. The chloroform layer was collected and evaporated under reduced pressure using rotary evaporation. The resultant residues were re-dissolved in 5 μL LCMS grade methyl alcohol and then subjected to Tandem Mass Spectrometric analysis (LTQ XL Linear Ion Trap Mass Spectrophotometer, Thermo Scientific, USA), using Electrospray (ESI) ionization probe. The system was operated by the Xcalibur software. Direct syringe pump method was used to inject the sample with a flow rate of 5 μL/min. Samples were scanned at both positive and negative total ion full scan modes (mass scan range m/z 50–2,000) with source voltage and capillary voltage of 4.8 kV and 23 V, respectively. Capillary temperature and sheath gas flow (N_2_) were 350°C and 30 arbitrary units during both scan modes. Fragmentation of selected analytes was done at both positive and negative ion modes using collision induced dissociation (CID) energy of 30 (% of 5 V). The mass spectra for molecule(s) present in CM3 were run against the NIST Mass Spectral Search Program for the identification of homologous compounds. The compounds identification was made after correlation with published data. The structures of novel compounds were ascertained using mechanistic Chemistry approach.

### Statistical analysis

Statistical analysis of the data was performed using GraphPad Prism version 8.0.2 (GraphPad Software, San Diego, CA, USA). The data are presented as the mean ± standard error of several independent experiments performed in duplicate. P value of < 0.05 was considered statistically significant for all parameters and the confidence interval (CI) was 95%. The statistical analyses were carried out using two-tailed Student’s t–test and each of the resultant P values for a comparison is shown in the appropriate text or in the figure legend.

### “Ethical approval” and consent to participate

This article does not contain any studies with human participants. The use of animals was approved by the Sunway University Research Ethics Committee, SUREC 2017/040. We also confirm that all experiments were performed in accordance with applicable rules and regulations.

## Results

### Isolation and identification of bacterial isolates derived from the gut of turtle

**Figure 1:**
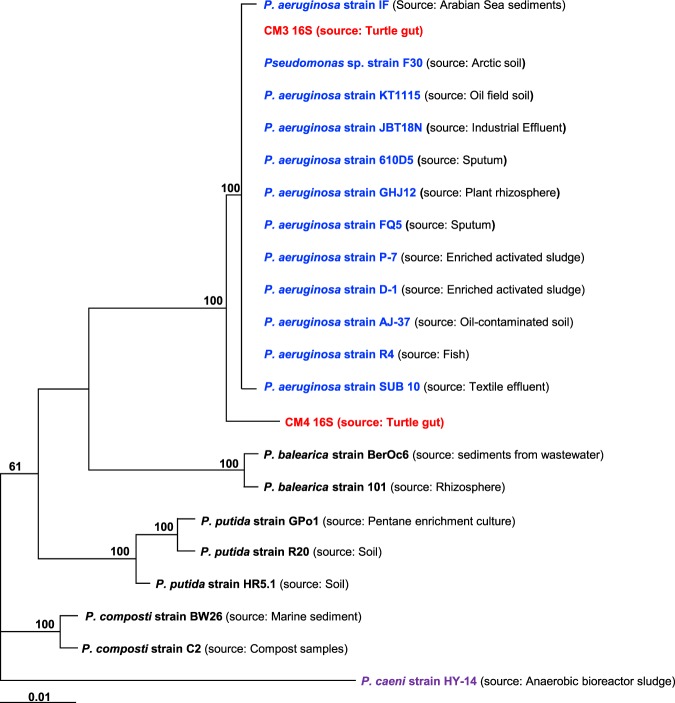
Phylogenetic tree representing 19 *Pseudomonas* strains with *Pseudomonas caeni* as the outgroup and two *Pseudomonas* strains used in this study (red) based on the phylogenetic analysis of 16 S rRNA genes. The dendogram was reconstructed using the Maximum Likelihood (ML) method (MEGA 7.) based on GTR + G model with concatenated 16 S rRNA sequences. Percentage bootstrap values was higher than 50% of 1000 replicates are indicated at branching nodes.

Several bacteria were isolated from the gut of turtle and sub-cultured on nutrient agar plates to obtain pure cultures (Table 2). Bacterial extracts were tested against selected Gram-positive and Gram-negative bacteria. Bacterial extracts with highest antibacterial activities were selected and their identification was done using microbiological, Analytical Profile Index (API) strips tests (data not shown) and 16 S rRNA gene amplification and sequencing. The results revealed *Enterobacter cloacae, Aeromonas hydrophila, Pseudomonas aeruginosa*, and *P. aeruginosa* (Fig. 1 and supplementary Fig. S4). The isolated bacteria were subjected to antibiotic sensitivity testing (data not shown). Among these bacteria, *E. cloacae* showed resistance to amoxicillin and cefuroxime while found sensitive to imipenem, gentamicin, ciprofloxacin, and sulbactam. *A. hydrophila* was found sensitive to tazobactam, ceftazidime, imipenem, ciprofloxacin, gentamicin, and cefoperazone. *P. aeruginosa* showed resistance to ceftazidime and ampicillin while sensitive to gentamicin, ciprofloxacin, imipenem, cefoperazone. *P. aeruginosa* was found sensitive to piperacillin, ciprofloxacin, ceftazidime, imipenem, gentamicin and cefoperazone. Bacterial conditioned media (CM) were prepared. The CM from different bacteria included CM1 (*E. cloacae*), CM2 (*A. hydrophila*), CM3 (*P. aeruginosa*), CM4 (*P*. *aeruginosa*) and CM5 (*E. coli* K-12) (Table 2).

**Table 2:** Bacterial species isolated from the gut of turtle.

Bacterial source	Conditioned medium
*Enterobacter cloacae*	CM1
*Aeromonas hydrophila*	CM2
*Pseudomonas aeruginosa*	CM3
*P. aeruginosa*	CM4
*E. coli* K-12	CM5

### Conditioned media of bacteria isolated from turtle gut exhibited broad-spectrum bactericidal activities

**Figure 2:**
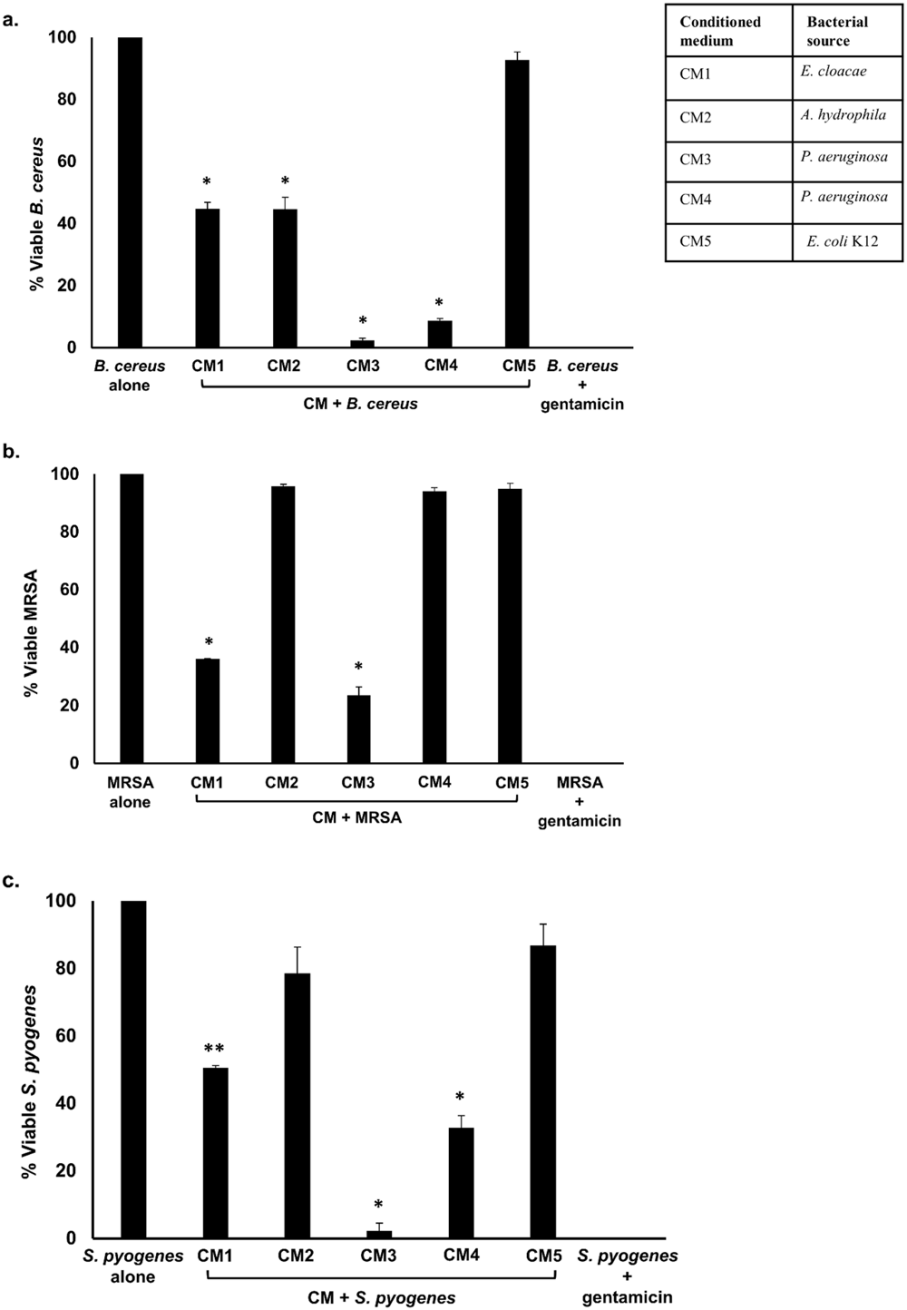
Turtle gut bacteria showed bactericidal activities against selected Gram-positive bacteria. Briefly, CM were incubated with different Gram-positive bacteria (1 × 10^6^) and their antibacterial effects were determined as described in Materials and Methods. The data are presented as the mean ± standard error of several independent experiments performed in duplicate. Student’s T-test was used to determine *P* values, (*) is *P* ≤ 0.05. Bacteria incubated with PBS and CM5 from *E. coli* K-12 were taken as negative and incubation with gentamicin (100 µg/mL) as positive controls. (**a**) CM when tested against *B. cereus*, (**b**) against MRSA and (**c**) against *S. pyogenes*. CM1 is *E. cloacae*, CM2 is *A. hydrophila*, CM3 is *P. aeruginosa*, CM4 is *P.* *aeruginosa* and CM5 is *E. coli* K-12.

When the CM were tested against MRSA, the results showed that CM1 and CM3 exhibited significant bactericidal effects (*P* < 0.05 using student’s t-test, two-tailed distribution) (Fig. 2a and Table 3). When tested against *S. pyogenes*, all CM except CM2 and CM5 exhibited bactericidal activities (*P* < 0.05) (Fig. 2b and Table 3). For *B. cereus*, all CM except CM5 demonstrated substantial antibacterial properties (Fig. 2c and Table 3) (*P* < 0.05).

**Table 3:** Representation of CM bactericidal effects against selected Gram-positive and Gram-negative pathogenic bacteria.

Conditioned Media	Antibacterial activities against Gram-positive bacteria			Antibacterial activities against Gram-negative bacteria				
	*B. cereus*	MRSA	*S. pyogenes*	*E. coli* K1	*K. pneumoniae*	*P. aeruginosa*	*S. marcescens*	*S. enterica*
CM1	+	+	+	+	+	+	+	-
CM2	+	-	-	+	-	+	+	+
CM3	+	+	+	+	+	+	+	+
CM4	+	-	+	+	+	+	-	+
CM5	-	-	-	-	-	-	-	-

**Figure 3:**
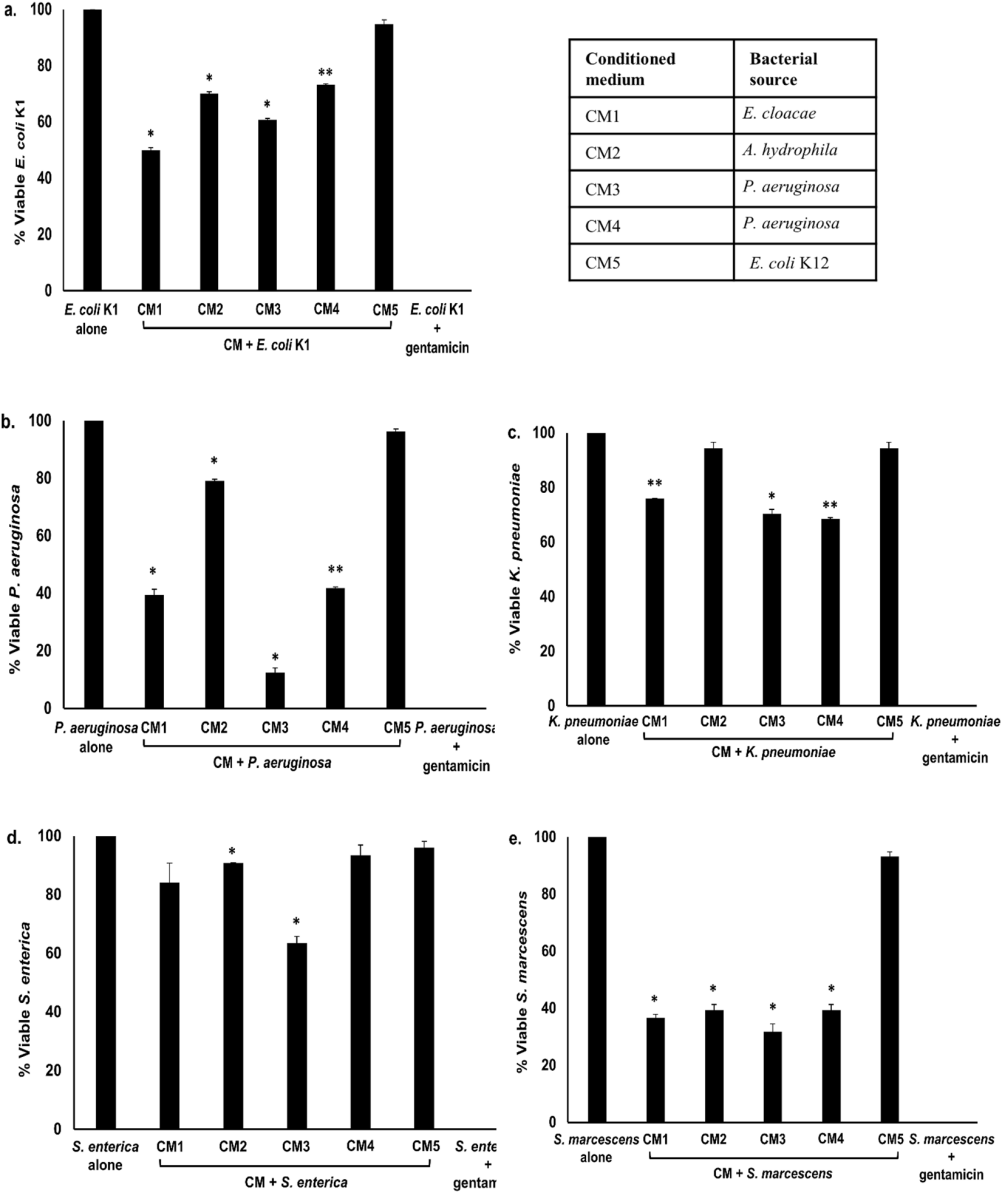
Conditioned medium from turtle gut bacteria demonstrated significant antibacterial effects against selected Gram-negative bacteria. Briefly, 1 × 10^6^ bacteria were incubated with turtle gut bacterial CM for 2 h at 37°C. Following this, serial dilution and plating onto nutrient agar plates were performed and plates were incubated overnight at 37°C. Bacterial colonies were enumerated on the following day. The data are expressed as the mean ± standard error of several independent experiments performed in duplicate. T-test statistics was performed to find *P* values, (*) is *P* ≤ 0.05. Bacteria incubated with gentamicin (100 µg/mL) while with PBS and CM5 were taken as positive and negative controls. (**a**) CM tested against *E. coli* K1, (**b**) against *P. aeruginosa* (**c**) against *K. pneumoniae* (**d**) against *S. enterica*, and (**e**) against *S. marcescens*. CM1 is *E. cloacae*, CM2 is *A. hydrophila*, CM3 is *P. aeruginosa*, CM4 is *P.* *aeruginosa *and CM5 is *E. coli* K-12.

Among Gram-negative bacteria, All the CM except CM5 showed antibacterial effects against *E. coli* K1 (*P* < 0.05) (Fig. 3a and Table 3). When the CM were screened against *P. aeruginosa*, all CM apart from CM5 exhibited significant bactericidal activities (*P* < 0.05) (Fig. 3b and Table 3). When tested against *K. pneumoniae*, all CM except CM2 and CM5 exhibited important antibacterial activities (Fig. 3c and Table 3) (*P* < 0.05). Against *S. enterica*, CM2 as well as CM3 exhibited significant bactericidal effects (*P* < 0.05) (Fig. 3d and Table 3) while all the CM except CM5 showed significant antibacterial activities against *S. marcescens* (*P* < 0.05) (Fig. 3e and Table 3). Overall, CM3 (*P. aeruginosa*) showed potent bactericidal effects against all bacterial isolates tested.

### CM-mediated bactericidal effects were heat-resistant

**Figure 4:**
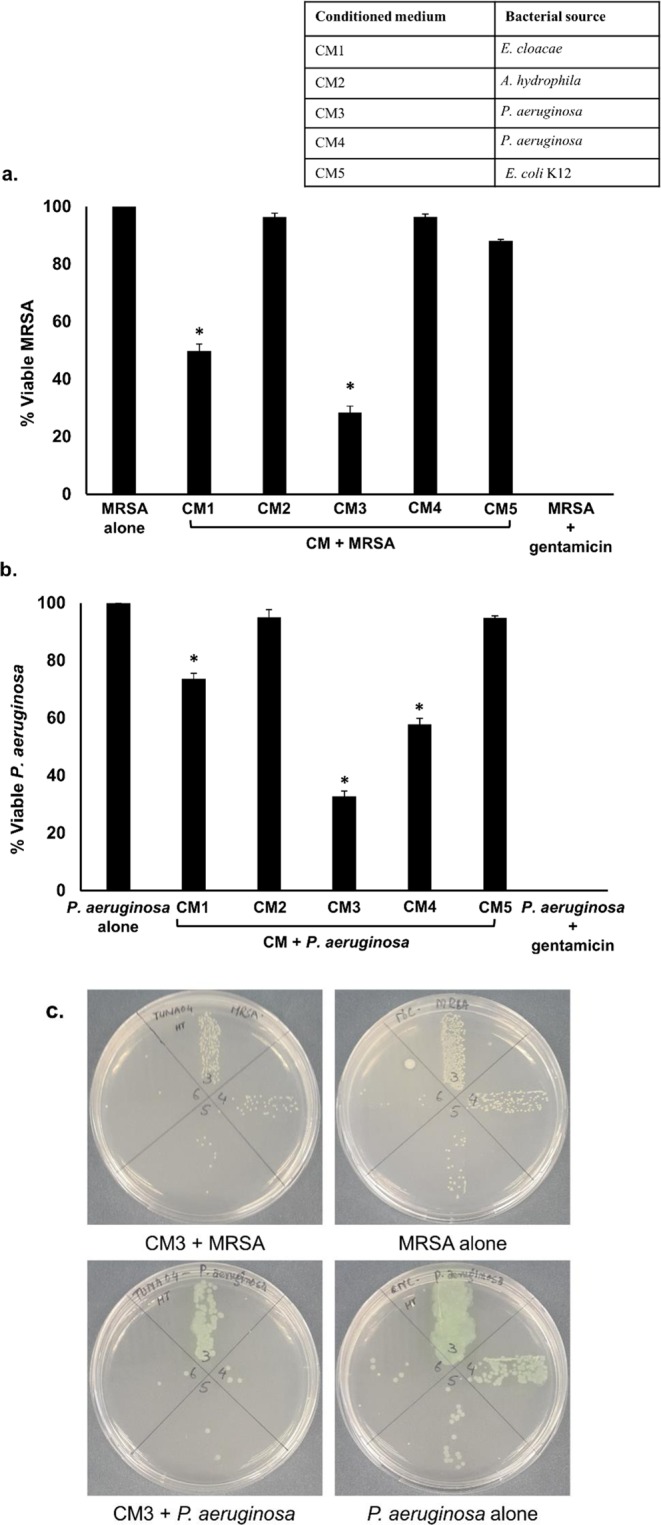
Heat inactivated CM from turtle gut bacteria exhibited bactericidal activities against *P. aeruginosa* and MRSA. Briefly, heat treated CM were incubated with 1 × 10^6^ bacteria at 37°C for 2 h. Next, the culture was serially diluted and then plated on nutrient agar plates. Plates were incubated for 24 h at 37°C and bacterial viability was measured by counting viable bacterial colonies. Experiments were performed several times in duplicate and *P* values were determined using T-test. (*) denotes *P* ≤ 0.05. (**a)** CM tested against MRSA (**b)** against *P. aeruginosa* and (**c)** representative effects of CM against MRSA and *P. aeruginosa*.

CM were heat inactivated at 95°C for 10 min and tested for their antibacterial effects against MRSA and *P. aeruginosa*. The results showed that CM1 and CM3 exhibited notable bactericidal activities against MRSA following heat-inactivation (*P* < 0.05) (Fig. 4a). When tested against *P. aeruginosa*, all CM except CM2 and CM5 exhibited significant antibacterial effects (*P* < 0.05) (Fig. 4b) suggesting that active molecule(s) are heat-resistant.

### Conditioned media of turtle gut bacteria showed minimal cytotoxicity

**Figure 5:**
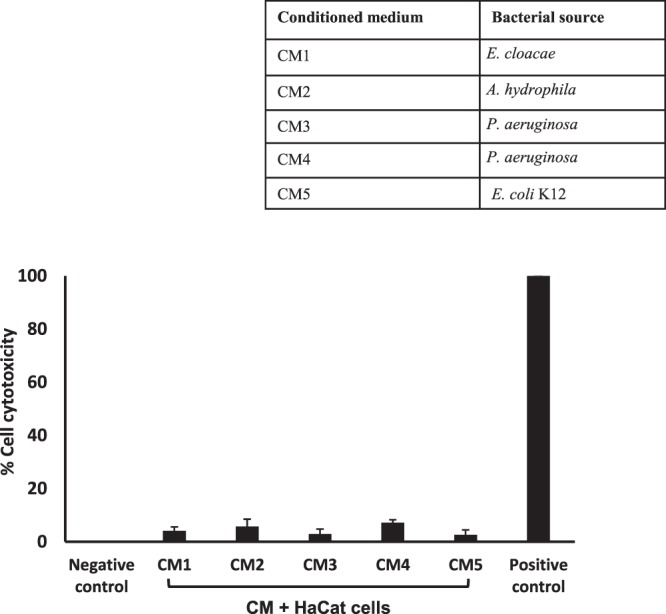
Cytotoxicity assays of turtle gut bacterial conditioned medium against HaCaT cell lines. Briefly, CM from the gut of turtle were incubated with HaCaT cells monolayer in a 96 well plate at 37°C for 24 h in the presence of 5% CO_2_ and humidified conditions. Following day, LDH released by cells was measured as described in methods. (**a)** All CM tested were non-toxic against HaCaT cells.

**Figure 6:**
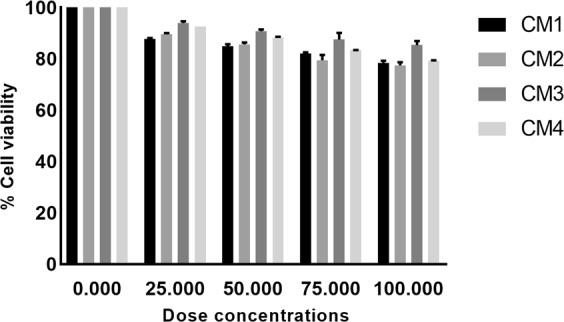
Effect of turtle gut bacterial CM in a graduate concentration/volume on HaCaT cell viability. Briefly, HaCaT cells were grown in a 96-well plate and incubated with various concentrations of CM for 24 h at 37°C in the presence of 95% humidity with 5% CO_2_. Cells incubated alone was taken as negative control. The data are expressed as mean ± standard error of three times independent experiments performed in duplicates. Data was analyzed using GraphPad prism software.

Furthermore, effects of the CM on human keratinocyte HaCaT cell viability were determined using lactate dehydrogenase assays. The results revealed that all CM tested produced minimal (less than 10%) cytotoxic effects against normal dermal cells (Fig. 5). To determine the cytotoxic effects of CM at graduated concentrations, HaCaT cell were exposed to CM using MTT assays. The extent of cytotoxicity from each concentration of CM was measured as percentage of cell viability. According to ISO 10993-5, cell viability above 80% is considered as non-cytotoxic; within 60% to 80% weak; 40% to 60% moderate while lower than 40% potent cytotoxic respectively^25^. The results revealed that the cell viability range was within 80% and 100% (Fig. 6). Among all the CM tested, CM3 were found best showed minimum cytotoxicity and higher cell viability (Fig. 6).

### Mass spectrometric analysis of CM extract revealed the presence of several secondary metabolites 

**Figure 7:**
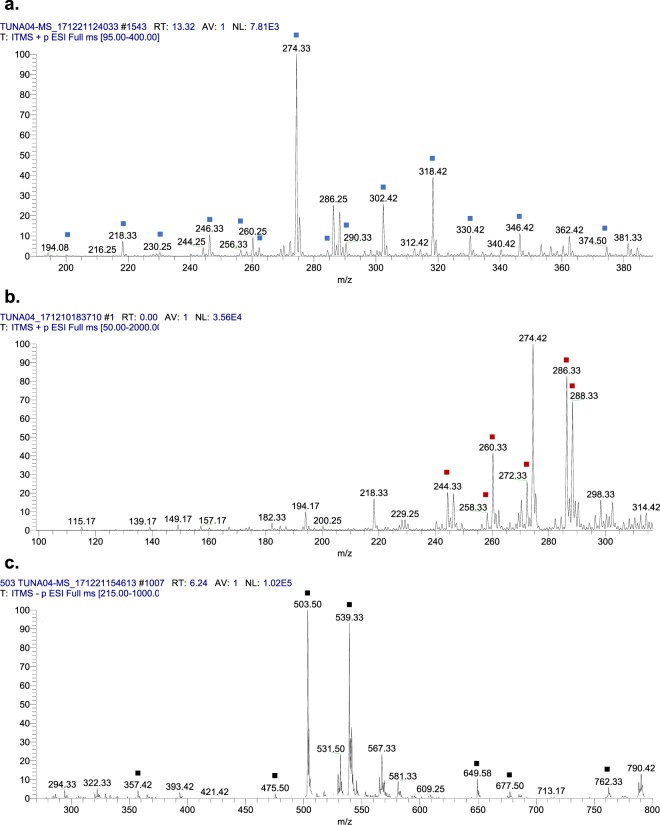
LCMS spectrum of *P. aeruginosa* (CM3) revealed the presence of **a)** *N*-acyl-homoserine lactone (AHL), **b)** 4-hydroxy-2-Alkylquinolines (HAQs) and **c)** rhamnolipids and their daughter ion species (labelled as blue, red and black).

Among all CM, CM3 (*P. aeruginosa*) showed profound broad-spectrum bactericidal effects against both, Gram-negative as well as Gram-positive bacteria. Hence, the CM3 was subjected to chloroform extraction, followed by evaporation and re-dissolving in methanol, which further analyzed by ESI-MS/MS. CM extract was injected using direct syringe pump method and analysis were performed at positive as well as negative ion scan modes. The results revealed that CM3 demonstrated several known and novel homologues of *N*-acyl-homoserine lactones (AHLs) having *m/z* ranged from 200 to 374 [M + H]^+^ (Fig. 7a). The structures of these molecules was confirmed by MS/MS, AHLs were further confirmed through tandem mass spectrometry by trapping their M + H^+^ ions in Ion Trap Mass Spectrometer and fragmenting them at 30 V of collision-induced dissociation (for details see materials and methods).

**Figure 8:**
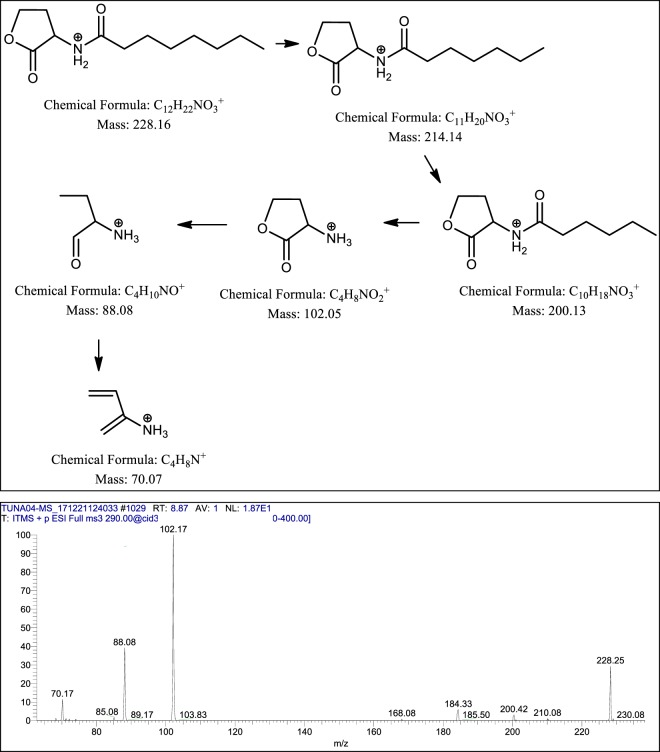
Putative structures of different fragments obtained by CID of the *m/z* 228 [M + H]^+^.

CM3 (*P. aeruginosa*) revealed the presence of a range of HAQ molecules (Table 4) and also presented three series of AHLs (Fig. 5). For latter, serial no. 1 was comprised of homoserine lactone ring with saturated straight acyl chains starting from C_5:0_ -C_5_H_11_ to C_11:0_ -C_11_H_23_ with low abundance (2-9%) (Table 5). All of the four homologues were confirmed with Tandem Mass Spectrometry. Their MS/MS have generated the daughter ion peaks *m/z* 88 and 102, these are the finger prints of AHLs (Fig. 8 and supplementary Fig. S1), which are in agreement with the published data reported previously^26,28,29^.

**Table 4:** CC_50_ and MNTD_90_ values of CM of turtle gut bacteria (CC_50_ and MNTD_90_ are the concentrations at which 50% and 90% cells survive).

Samples	CC_50_	MNTD_90_
CM1	694.7	17.56
CM2	407.9	21.86
CM3	851.1	46.66
CM4	374.1	33.94

**CC**_**50**_ = Cytotoxic concentration

**MNTD**_**90**_ = Maximum non-toxic dose.

**Table 5:** Metabolites produced by *Pseudomonas aeruginosa* identified by ESI-MS and tandem mass spectrometry.

S. No.	Structures of metabolites	Side Chains	Observed Peaks (m/z)			MS/MS (Verified)*	MS/MS (Reported)	References
	N-acyl-homoserine lactones (AHLs)		[M + H]^+^		[M−H]^−^			
			m/z	Abundance*				
1		C_5:0_-**C**_**5**_**H**_**11**_	200	2	-	(+) 172, 156, 102, 88.	-**	^29^
		C_7:0_ -**C**_**7**_**H**_**15**_	228	9	-	(+) 214, 200, 102, 88, 70.	-**	^26,28^
		C_9:0_ -**C**_**9**_**H**_**19**_	256	3	-	(+) 228, 212, 200, 102, 88.	-**	^26^
		C_11:0_-**C**_**11**_**H**_**23**_	284	9	-	(+) 256, 240, 102, 88.	-**	^26^
2		C_5:0_ -**C**_**5**_**H**_**11**_	218	20	-	(+) 200, 174, 156, 142, 116, 106, 102, 88, 70.	-**	^28^
		C_7:0_ -**C**_**7**_**H**_**15**_	246	25	-	(+) 228, 202, 184, 106, 102, 88.	-**	^28^
		C_9:0_-**C**_**9**_**H**_**19**_	274	100	-	(+) 256, 230, 212, 106, 102, 88.	-**	^26^
		C_11:0_-**C**_**11**_**H**_**23**_	302	18	-	(+) 284, 258, 240, 106, 102, 88.	-**	^26^
		C_13:0_ –**C**_**13**_**H**_**27**_	330	7	-	(+) 312, 286, 268, 106, 102.	-**	^26^
3		C_6:0_ -**C**_**6**_**H**_**13**_**O**_3_^***^	262	13	-	(+) 244, 200, 102, 88.	-**	Current study
		C_8:0_-**C**_**8**_**H**_**17**_**O**_3_^***^	290	18	-	(+) 272, 228, 102, 88.	-**	Current study
		C_10:0_-**C**_**10**_**H**_**21**_**O**_3_^***^	318	75	-	(+) 300, 256, 102.	-**	Current study
		C_12:0_–**C**_**12**_**H**_**25**_**O**_3_^***^	346	12	-	(+) 328, 284, 102, 88.	-**	Current study
		C_14:0_–**C**_**14**_**H**_**29**_**O**_3_^***^	374	5	-	(+) 356, 312, 102.	-**	Current study
	**4-hydroxy-2-Alkylquinolines (HAQs)**							
4		C_7:0_–**C**_**7**_**H**_**15**_	244	22	242	(+) 226, 186, 172, 159, 146. (−) 228, 198, 170, 158, 158, 144, 143.	(+) 200, 188, 186, 172, 159, 146. (+) 186, 172, 159, 146.	^27,50^
		C_8:0_–**C**_**8**_**H**_**17**_	258	10	-	(+) 244, 230, 226, 216, 202, 198, 188, 186, 184, 174, 172, 170, 162, 160, 159, 156, 146, 132.	(+) 240, 224, 198, 188, 186, 172, 159, 146.	^27,47^
		C_9:0_–**C**_**9**_**H**_**19**_	272	29	270	(+) 258, 254, 240, 226, 216, 202, 198, 188, 186, 172, 162, 160, 159, 146, 132. (−) 242, 226, 212, 198, 184, 170, 157.	(+) 186, 172, 159, 146. (−) 184, 170, 158, 157, 144. (+) 184, 172, 159.	^27,34^
5		C_8:1_–**C**_**8**_**H**_**15**_	286	85	284	(+) 268, 258, 254, 244, 240, 230, 226, 216, 212, 202. (−) 270, 256, 240, 228, 186, 173, 159.	(+) 268, 258, 240, 226, 216, 212, 202, 198, 188, 186, 184, 174, 172, 162, 160, 159, 146, 132.	^27,50^
6		C_7:0_–**C**_**7**_**H**_**15**_	260	42	258	(+) 242, 228, 214, 200, 186, 175, 172, 162, 159. (−) 240, 230, 214, 186, 173, 159, 144.	(+) 188, 175. (+) 242, 186, 175, 172, 162, 159. (−) 241, 240, 230, 214, 187, 173, 172, 159, 144.	^27,34^
	**Rhamnolipids**							
**Mono-rhamno-di-lipidic congers**								
7		**Rha-C**_**10**_**-C**_**10**_	-	100	503	(−) 474, 362, 339, 334, 324, 306, 169.	-**	^51^
		**Rha-C**_**10**_**-C**_**10**_	-	9	504	(−) 361, 340, 169.	-**	^52^
**Di-rhamno-di-lipidic congers**								
8		**Rha-Rha-C**_**10**_**-C**_**10**_	-	19	649	(−) 479, 339.	-**	^51^
9		**Decenoyl-Rha-Rha-C**_**10**_**-C**_**10**_	803	62	-	(+) 301.	-**	^35^

*Relative ions abundance was measured from full scan MS, **MS/MS not reported, *** Structures have been verified by Tandem Mass Spectrometry (see Fig. 10 and Supplementary Figures S1 (viii, ix, x, xi)).

**Figure 9:**
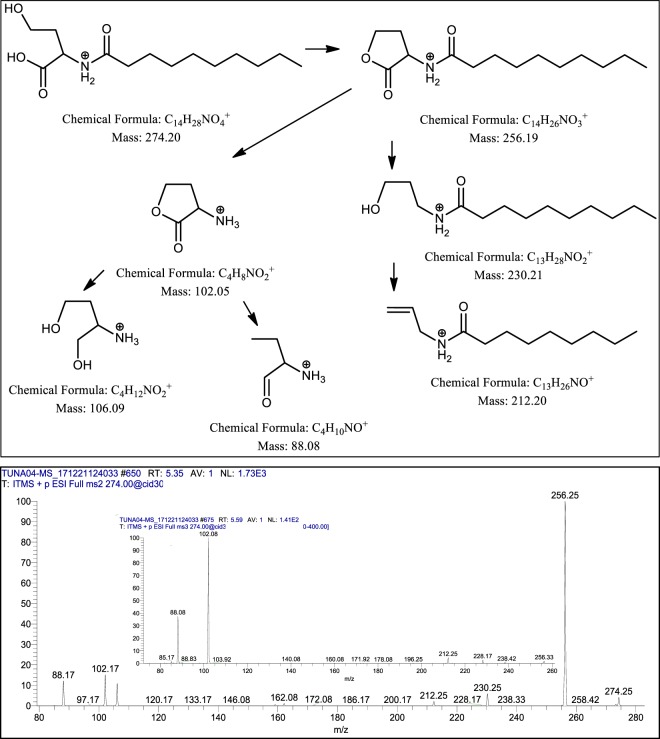
Profiling of the fragmentation data obtained from tandem mass spectrometry of *m/z* 274 [M + H]^+^.

The second series AHLs demonstrated open lactone ring structures with saturated straight acyl chains ranged from *m/z* 218 – 330 (Table 5, Serial No. 2). This sub-class of AHLs represented the higher abundance as compared to Serial No. 1 and 3 compounds. An AHL having *m/z* at 274 [M + H]^+^, indicated the maximum peak intensity (base peak) in MS full scan positive ion mode (Fig. 7a). As a representative AHL of open lactone ring series, the chemistry of this compound was comprehensively studied employing CID at positive ion mode (Fig. 9). Upon CID, the molecule spontaneously lost one mole of H_2_O, leaving a stable ion at *m/z* 256 (AHL). Further fragmentation of *m/z* 256 produced the anticipated daughter ion peaks at *m/z* 230, 212, 106, 102 and 88 (Fig. 9) The presence of daughter ion peaks at *m/z* 88, 102 and 106 confirmed the structure because these are considered as finger prints of AHLs^30^. Similarly, the other AHLs having open lactone ring follow the similar pattern of fragmentation (as does *m/z* 274) during MS/MS by instantaneously loosing water to produce stable daughter ion base peaks at *m/z* 218 – 18 = 200, *m/z* 246 – 18 = 228, *m/z* 302 – 18 = 284 and *m/z* 330 – 18 = 312 (Table 5, Serial No. 2; Supplementary Fig. S1). The second round MS/MS of the daughter ion peaks at *m/z* 200, *m/z* 228, *m/z* 284 and *m/z* 312 have produced the *m/z* 88, 102 and 106, which can confirm the AHLs structures. Additionally, such open lactone ring AHLs have also been reported^26,28^.

The third series AHLs were found to be the most interesting, have demonstrated homoserine lactone ring acylated with saturated straight chains having additional oxygens, positioned most probably, at the terminal of the side chains. The second aspect of this sub-class is their higher masses (than usual AHLs), ranged from *m/z* 262 – 374 (Table 5, Serial No. 3). This sub-class of AHLs demonstrated the higher abundance as compared to Serial No. 1 compounds. The structure elucidation of these AHLs seems complex. Using Tandem Mass Spectrometry and data analysis based on mechanistic Chemistry approach, the structures of these compounds have been assigned.

**Figure 10:**
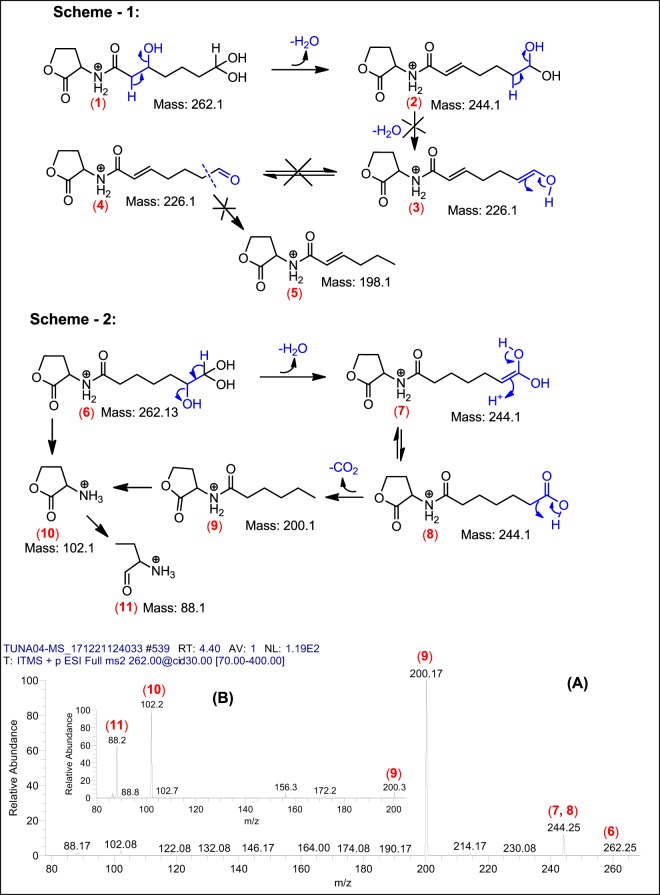
Illustrative structures of the fragments generated through the tandem mass spectrometry of *m/z* 262 using CID (energy 30.0) at positive ion mode.

As a representative to this sub-class the proposed AHL with *m/z* 262 was fragmented through collusion induced dissociation, which generated AHLs signature / confirmatory peaks at *m/z* 88 and *m/z* 102 (Table 5, Serial No. 3). Additionally, this fragmentation produced daughter ions at *m/z* 244 (-18, loss of 1H_2_O) and subsequently *m/z* 200 (-44, loss of 1CO_2_) as a base peak, which revealed that one of the oxidized carbon is present at the end of the side chain (at terminal position). However, the position of hydroxyl group, which yield the loss of 1H_2_O and gave daughter ions at *m/z* 244, needs further investigation. Generally, the presence of oxygen in the form of β-hydroxy group (3OH-C_n_-HSL) on acyl chains of AHLs, is widely reported^26,28,30^. On the basis of these facts, initially the anticipated structure (**1**) was proposed (Fig. 10, scheme 1), which on collision-induced dissociation @ (30 V) yielded daughter ion (**2**) having *α*-*β* enone moiety at *m/z* 244 by losing 1 mole of water [-18 mass] with relative ion abundance of less than 20%. On contrary, when authentic 3OH C8-HSL sample having *m/z* 244 was fragmented at similar energy of 30 V (Fig. 10), it yielded *m/z* 226 (after losing 1 M H_2_O) with 100% relative ion abundance, which is far higher abundance as compared to daughter ion (**2**). *β*-Hydroxy carbonyls are well known to produce daughter ions with high relative ion abundance (generally base peak) having *α*-*β* enone moiety under Mass Spectrometric fragmentation^31^. This lack of ion abundance correlation puts doubts on the validity of structure (**1**), which generated further uncertainty on fragmentation of structure (**2**) (Fig. 10, scheme 1), because none of the anticipated daughter ions (**3**), (**4**) and (**5**) proposed in the Scheme-1, could be spotted in the Mass Spectrum (Fig. 10, Mass Spectrum **A**).

Considering the second school of thought, which reveals that the two hydroxyl groups are positioning at terminal acyl carbon chain (geminal position) and 3^rd^ hydroxyl group is present on the second last carbon (vicinal position), as illustrated in the proposed structure (**6**) (Fig. 10, Scheme 2). Fragmentation of structure (**6**) yielded the anticipated daughter ion (**7**) at *m/z* 244, which after *keto-enol* isomerization (as per of the mechanistic expectation) can yield structure (**8**) having similar mass (*m/z* 244). The structure (**8**) has undergone a decarboxylation (cleavage of terminal carboxylate) during collision-induced dissociation (30 V) to produce daughter ion (**9**) at *m/z* 200 with 100% relative abundance (as anticipated, based on fragmentation mechanism). Decarboxylation at terminal position of alkyl chains, during tandem mass spectrometry, are quite usual and is well reported in literature^31^.

During round-1 fragmentation of structure (**6**), all of the structures (**6-11**) proposed in scheme-2, were spotted in the mass spectrum, including the AHLs’ signature daughter ions at *m/z* 102 and *m/z* 88 (Fig. 10, Mass Spectrum A). Moreover, fragmentation of daughter ion *m/z* 200 (structure **9**) also produced the AHLs’ signature daughter ions at *m/z* 102 (**10**) and *m/z* 88 (**11**) (Fig. 10, Mass Spectrum B), further confirming the validity of Scheme-2.

Intriguingly, the presence of all of the daughter ions (in tandem mass spectrum), proposed in Scheme-2, proved to be the structure (**6**) of AHL having *m/z* 262 in full scan, with significant level of confidence. Moreover, the other homologues AHLs having oxidized side chains, proposed in Table 5 (Serial No. 3) exhibiting extended acyl chains of (CH_2_)_2_ with addition of 28 mass units i. e. *m/z* 290, *m/z* 318, *m/z* 346 and *m/z* 374, have also followed the similar fragmentation pattern, as proposed in Scheme-2 during their tandem mass spectrometric analysis (Supplementary Fig. S1).

### Mass spectrometric analysis of 4-hydroxy-2-Alkylquinolines (HAQs)

**Figure 11:**
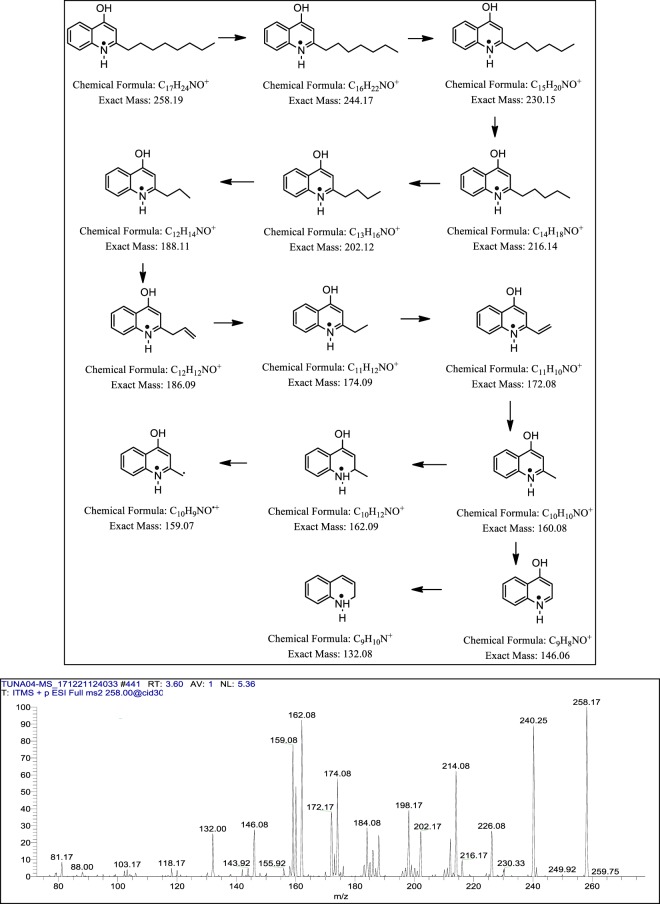
Putative structures of the fragment ions generated through MS/MS of the *m/z* 258 [M + H]^+^ by CID (energy 30.0) at positive ion mode.

Following, ESI-MS and tandem mass spectrometry analysis, CM3 revealed the presence of a range of HAQ molecules. The prominent peaks of HAQs produced by *P. aeruginosa* having *m/z* from 244 to 288 [M + H] ^+^ as shown in (Fig. 7b). These HAQs were classified in several group based on the presences of hydrogen, hydroxyl (OH) and alkyl (R) groups at two and three positions in the heterocyclic ring as shown in (Table 5). HAQs having *m/z* at 244, 258 and 272 [M + H] ^+^ represent analogues having hydrogen at positon 3 in the ring (Table 5). These HAQs are different from each other on the basis of saturation and unsaturation in the alkyl group chain length. An HAQ having mass to charge ratio at 258 [M + H] ^+^, representing 2-octyl-4-hydroxyquinoline (HHQ) (Fig. 11). When the collision induced dissociation is applied to this molecule, the molecule lost several CH_2_ moles leaving 2-heptyl-4-hydroxyquinoline, 2-hexyl-4-hydroxyquinoline, 2-pentyl-4-hydroxyquinoline and alkyl side chain is detached from the heterocyclic ring. The parent peak i.e. *m/z* 258 [M + H] ^+^ upon fragmentation yielded the daughter ions as shown in (Table 5). Likewise, HAQs having *m/z* at 260 [M + H] ^+^, demonstrating another analogue of this series where hydrogen at position 3 in the heterocyclic ring is substituted with an OH group called 3,4-dihydroxy-2-heptylquinoline (HHAQ)^32^. On collision induced dissociation, HHAQs shed off water molecule leaving molecule with *m/z* at 242 as shown in (Supplementary Fig. S2 IX). Further fragmentation produced the daughter ion peaks as shown in Table 5. Another HAQs analogue i.e. *m/z* at 288 [M + H] ^+^, indicating structure in which hydrogen at position 1 and position 3 are converted to OH and methyl (CH_3_) groups respectively. Upon fragmentation using CID, this molecule lost one water molecule leaving compound having *m/z* at 270 [M + H] ^+^ whereas, on the other hand CID resulted in alkyl side chain reduction as shown in (Supplementary Fig. S2 VIII) yielded daughter ions presented in (Table 5). For the remaining HAQs peaks MS/MS was performed having *m/z* at 244, 258, 272, 272and 286 [M + H]^+^ as shown in the supplementary Fig. S2.

### Mass spectrometric analysis of rhamnolipids

**Figure 12:**
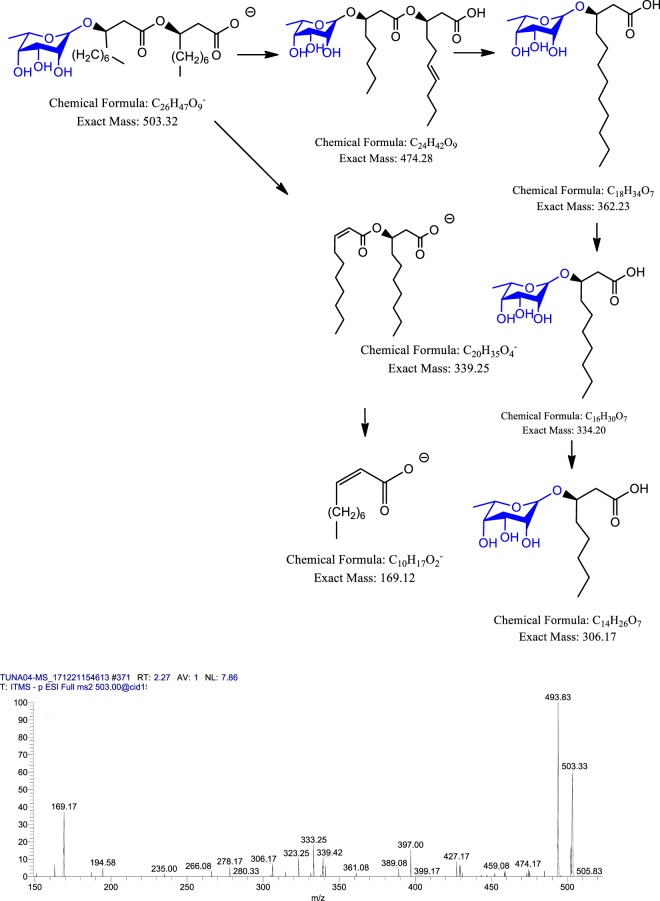
Fragmentation pattern and product ion spectra of rhamnolipids having *m/z* at 503 [M-H]^-^.

Rhamnolipids are glycolipids with diverse structures synthesized by several bacterial species. It was initially discovered as exoproducts of pathogenic Gram-negative *P. aeruginosa*^33^. CM3 was analyzed through ESI-MS direct injection method at both positive and negative full scan mode. Further, tandem mass spectrometry analysis of CM3 revealed numerous rhamnolipids including mono-rhamno-mono-lipidic congeners, mono-rhamno-di-lipidic congeners, di-rhamno-mono-lipidic congeners and di-rhamno-di-lipidic congeners peaks (Fig. 7c). Among rhamnolipids peaks, rhamnolipids having *m/z* at 503 [M-H]^-^ was subjected to MS/MS analysis due to its higher ion abundance. Upon CID, fragmentation of this molecule resulted in the liberation of two CH_2_ molecules from first fatty acid chain leaving *m/z* at 474 (Fig. 12). Further fragmentation of this molecule produced its daughter ion peaks listed in (Table 5). Other rhamnolipids such as 504, 649 and 803 produced by *P. aeruginosa* were characterized using MS/MS analysis while 762 was analyzed using ESI-MS only as shown in the supplementary Fig. S3.

## Discussion

Natural environments harbour diverse collection of microbial species. In particular, bacteria compete with their neighbours in a constant battle for space and nutrients acquisition^34^. To achieve success, bacteria are well-known to produce molecules that are growth inhibitory to other microbes. The producer strains are resistant to these molecules that are often secondary metabolites^12,35,36^. Since the discovery of penicillin, this area of research has transformed medicine in our fight against bacterial infections and remains at the forefront in our search for novel antibacterials. For example, Guo *et al*., (2015) isolated Actinomycetes from the red soil producing six novel secondary metabolites with biological activities^37^. Similarly, Claverías *et al*., (2015) isolated marine Actinobacteria producing diverse secondary metabolites with broad spectrum antibacterial activities against pathogenic bacteria^38^. Andrimid and Zeamine are antibiotics with broad spectrum activities produced by *Serratia plymuthica* with powerful antibacterial, antifungal and anticancer activities^39^. Dreyer *et al*., (2018) isolated *Xenorhabdus* species that associated with *Steinernema* genus of nematodes produce several antibacterial, antifungal compounds and anticancer compounds^40^.

In the present study, we isolated gut bacteria of turtle, identified and cultured in RPMI to prepare the CM. The CM were screened against selected Gram-positive and Gram-negative bacteria to determine their bactericidal effects. Furthermore, CM were partially characterized by heat inactivation at 95 °C for 10 min and then tested for antibacterial properties. The results revealed that CM showed profound antibacterial activities against selected bacteria and the activity was heat-resistant suggesting that the bioactive molecules may be secondary metabolites. CM did not affect human skin (HaCaT) cell lines. Among all CM, CM3 from *P. aeruginosa* was selected for further analysis. CM3 extract were prepared and subjected to Tandem Mass spectrometric. The results demonstrated that *P. aeruginosa* (CM3) produced a variety of secondary metabolites including i.e., HAQs, a series of already reported ae well as novel AHLs and a various homologues of rhamnolipids (Fig. 10 series 2, Table 5 serial No. 3 and Supplementary Fig. S1). These molecules may be mutually or individually responsible for the notable bactericidal activities against several selected pathogenic bacteria.

The molecular ion peaks with higher abundance were selected for further analysis and the results from tandem mass spectrometry revealed a large repertoire of AHLs molecules. These AHLs molecules were classified on the basis of their structures as shown in (Table 5, S1 in supplementary data). AHLs are involved in quorum sensing of this pathogen^41^. Previously, Patel *et al*., (2016) screened Gram-negative bacteria including *P. aeruginosa* for AHLs involved in cell-to-cell communication^26^. In another study, Kušar *et al*., (2016) quantified AHLs from clinical samples of *P. aeruginosa* isolated from dog with otitis externa^29^, while Chen *et al*., (2013) isolated *Pseudomonas putida* from human tongue that produced two types of AHLs, *N-*octanoylhomoserine lactone and *N-*dodecanoylhomoserine lactone^42^. In our study, we isolated *P. aeruginosa* from the turtle gut which is an untapped source that produced known as well as novel AHLs molecules. To our knowledge, for the first time we report the isolation and characterization of AHLs molecules from the turtle gut bacteria.

Different bacterial species have been shown to produce different AHLs^26^. The basic structure of AHLs consists of a homoserine lactone ring adjoined with an *N*-acyl chain. The fatty acyl chain can vary in length from four to 14 carbon atoms, the chain may or may not contain an oxo-, or hydroxy-group at the 3-carbon position^26,43^, saturation, varied degree of unsaturation (*Z*, *E*, or both geometries), straight chain, methyl branched^43^ and *ρ*-coumaroyl^44^ in the bacteria isolated from diverse environments. However, according to the best of our knowledge, so far the AHLs structure (**6**) and its homologues, as described in Table 5 Serial No. 3 (Supplementary Fig. S1) are not yet reported in literature. It is still not clear from where these unusual acyl chains were acquired by bacteria. Generally, bacteria use the fatty acyl chains from their cellular pool, however, in some exceptional cases, acyl chains are reported to be acquired from environment sources i.e. the *Rhodopseudomonas palustris*, *Bradyrhizobium sp*. and *Silicibacter pomeroyi* use an acyl-HSL synthase to produce highly unusual ρ-coumaroyl-HSL by using environmental ρ-coumaric acid rather than fatty acids from cellular pools^44^. There is a possibility that the AHLs analogues having highly oxidized acyl chain (along with other AHLs) may have significant ecologic role in the diverse QS pathways of the *Pseudomonas aeruginosa* to survive in highly specific environment of turtle gut in the presence of other microbes and several host factors.

In addition, we detected 4-hydroxy-2-alkylquinolines (HAQs) molecules. These molecules are known to exhibit antibacterial activities and are involved in quorum-sensing as well as regulate virulence factors^45^. The CM3 analysis using ESI-MS/MS revealed ion peaks of HAQs molecules. Few of these HAQs were classified into groups based on differences in the alkyl side chain or even in the heterocyclic ring as shown in Table 5. The differences in the ring structures include the substitution of hydrogen or hydroxyl group at the position two or three and *N-*oxide group in place of the quinoline nitrogen. Recently, Yasmin *et al*., (2014) and (2017) identified several HAQs molecules from *P. aeruginosa* isolated from the plants rhizosphere^27,46^. In another study, Lépine *et al*., (2004) isolated several HAQs molecules from the culture supernatant of *P. aeruginosa*^34^. Déziel *et al*., identified numerous HAQs from a wild-type *P. aeruginosa*^47^. Here, for the first time we mined gut bacteria from novel source i.e. turtle gut, producing several HAQs molecules. Given the unique source, it is anticipated that these molecules are of value for further research in the rational development of potential antibacterials.

The mass spectrometric analysis of CM3 also revealed rhamnolipids (Table 5). Our findings are supported by Dobler *et al*., (2017) where they isolated rhamnolipids from wild-type as well as modified *P. aeruginosa*^48^. In another study, Cheng *et al*., (2017) isolated a novel *P. aeruginosa* strain from petroleum sludge that producing rhamnolipids that efficiently emulsify crude oil^49^. Given that turtles thrive in unhygienic conditions, their gut bacteria are of potential value to mine for potential rhamnolipids. Further characterization and functional studies of compounds isolated in this study could be a basis for the rational development of therapeutic antibacterials. These findings are significant and open several avenues for further studies in our search for novel antibacterials. In this regards, different types of animals/invertebrates inhabiting polluted environments as well as microbiota of such environments should be explored. Additionally, the immune system of such animals should be explored for potential antibacterial molecule(s).

## Conclusions

In summary, here we report the isolation of *Pseudomonas aeruginosa* from the gut of *Cuora amboinensis* (turtle). Conditioned media produced from *Pseudomonas* showed broad-spectrum antibacterial activities against several Gram-positive (*B. cereus*, *S. pyogenes* and MRSA) and Gram-negative (*E. coli* K1, *S. marcescens, P. aeruginosa, S. enterica* and *K. pneumoniae*) bacteria in a heat-resistant manner. Tandem Mass Spectrometric analyses revealed the presence of various secondary metabolites, i.e., a series of known as well as novel *N-*acyl-homoserine lactones, several homologues of 4-hydroxy-2-alkylquinolines, and rhamnolipids that may be of potential value in the rational development of chemotherapeutic interventions against bacterial infections.

## Supplementary information

Supplementary info
