# Supplementary material for: Gut bacteria of Cuora amboinensis (turtle) produce broad-spectrum antibacterial molecules
Source: Sci Rep. 2019 Nov 18;9:17012. doi: 10.1038/s41598-019-52738-w (PMC6861250; doi:10.1038/s41598-019-52738-w)
Supplement: Supplementary file 1 — Supplementary info [file 41598_2019_52738_MOESM1_ESM.pdf]

Gut bacteria of *Cuora amboinensis* (turtle) produce broad-spectrum antibacterial molecules

[1#]Noor Akbar, [1#]Naveed Ahmed Khan, [1]K Sagathevan, [2*]Mazhar Iqbal, [2]Abdul Tawab, [1*]Ruqaiyyah Siddiqui

Fig. S1 Profiling of the fragmentation data of various known and novel AHLs generated through the MS/MS of **I)** *m/z* 200, **II)** *m/z* 256, **III)** *m/z*284, **IV)** *m/z*218, **V)** *m/z* 246, **VI)** *m/z* 302, **VII)** *m/z* 330, **VIII)** *m/z* 290, **IX)** *m/z* 318, **X)** *m/z* 346, **XI)** and **XII)** *m/z* 374 $[M+H]^+$ by CID (energy 30.0) at positive ion mode.

I.

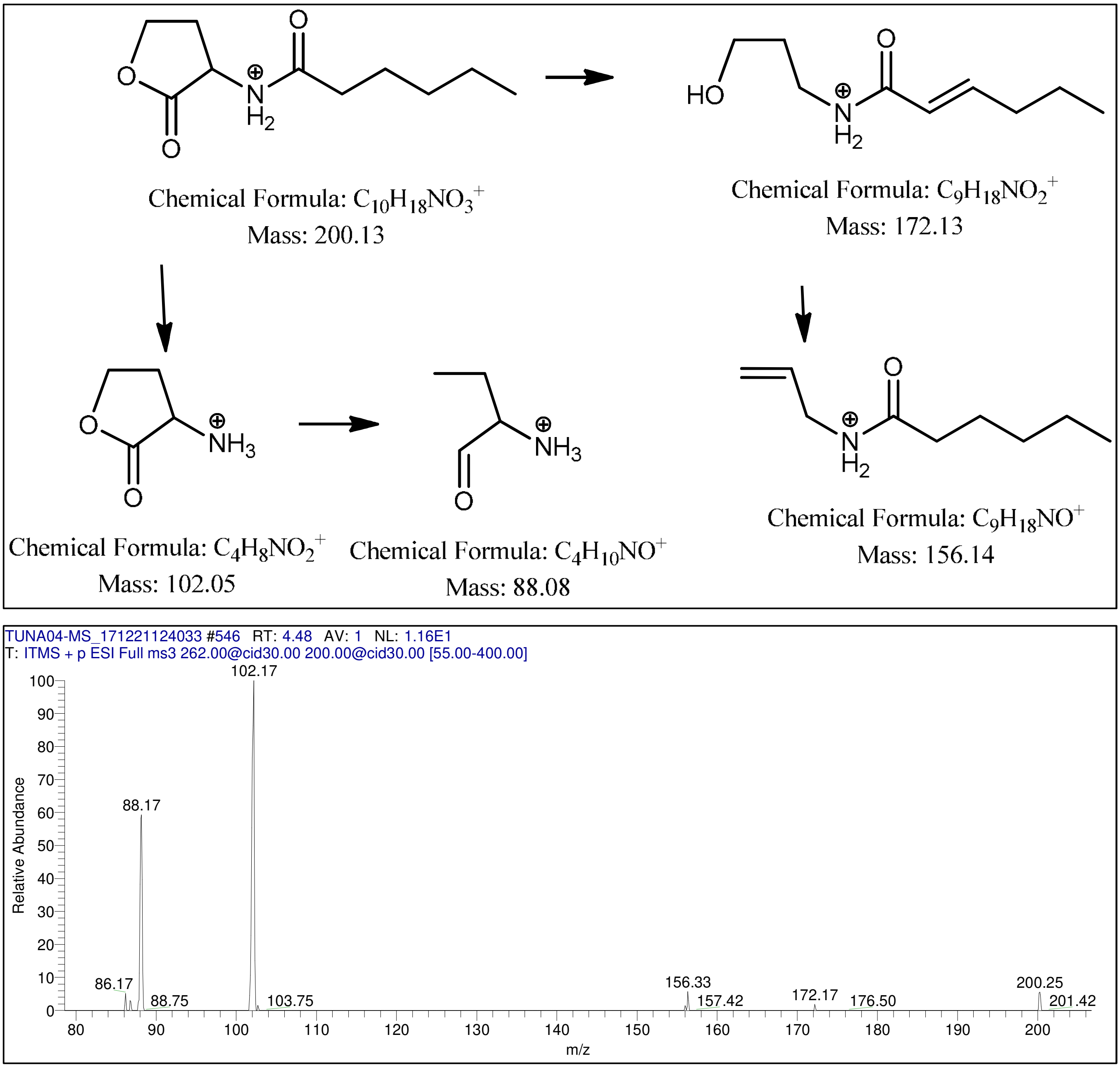

II.

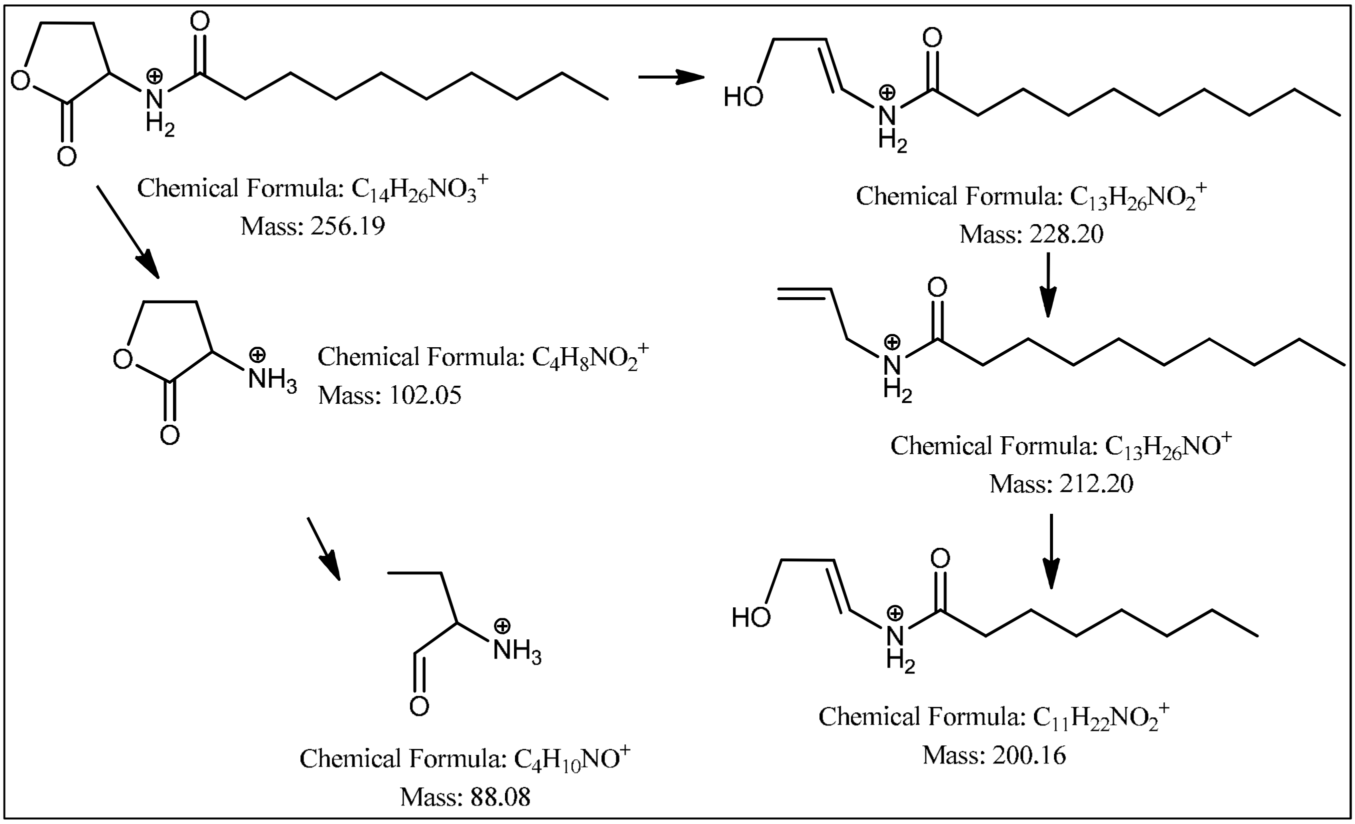
Chemical Formula: $C_{14}H_{26}NO_3^+$
Mass: 256.19
Chemical Formula: $C_{13}H_{26}NO_2^+$
Mass: 228.20
Chemical Formula: $C_4H_8NO_2^+$
Mass: 102.05
Chemical Formula: $C_{13}H_{26}NO^+$
Mass: 212.20
Chemical Formula: $C_{11}H_{22}NO_2^+$
Mass: 200.16
Chemical Formula: $C_4H_{10}NO^+$
Mass: 88.08

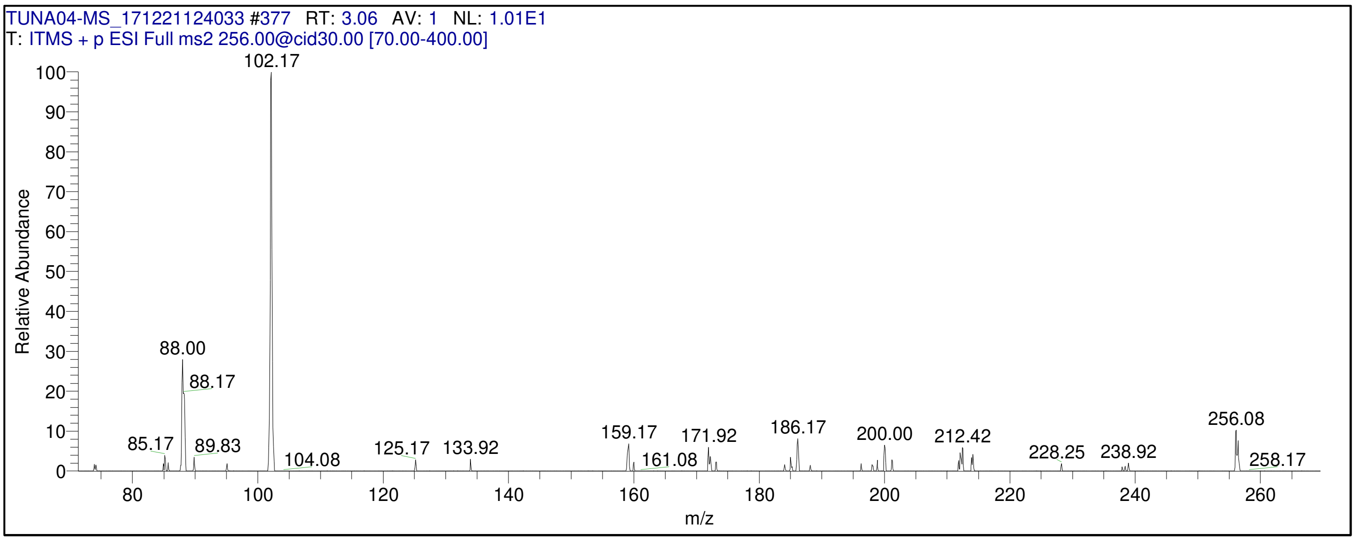
TUNA04-MS_171221124033 #377 RT: 3.06 AV: 1 NL: 1.01E1
T: ITMS + p ESI Full ms2 256.00@cid30.00 [70.00-400.00]
Relative Abundance
m/z
102.17
88.00
88.17
85.17
89.83
104.08
125.17
133.92
159.17
161.08
171.92
186.17
200.00
212.42
228.25
238.92
256.08
258.17

III.

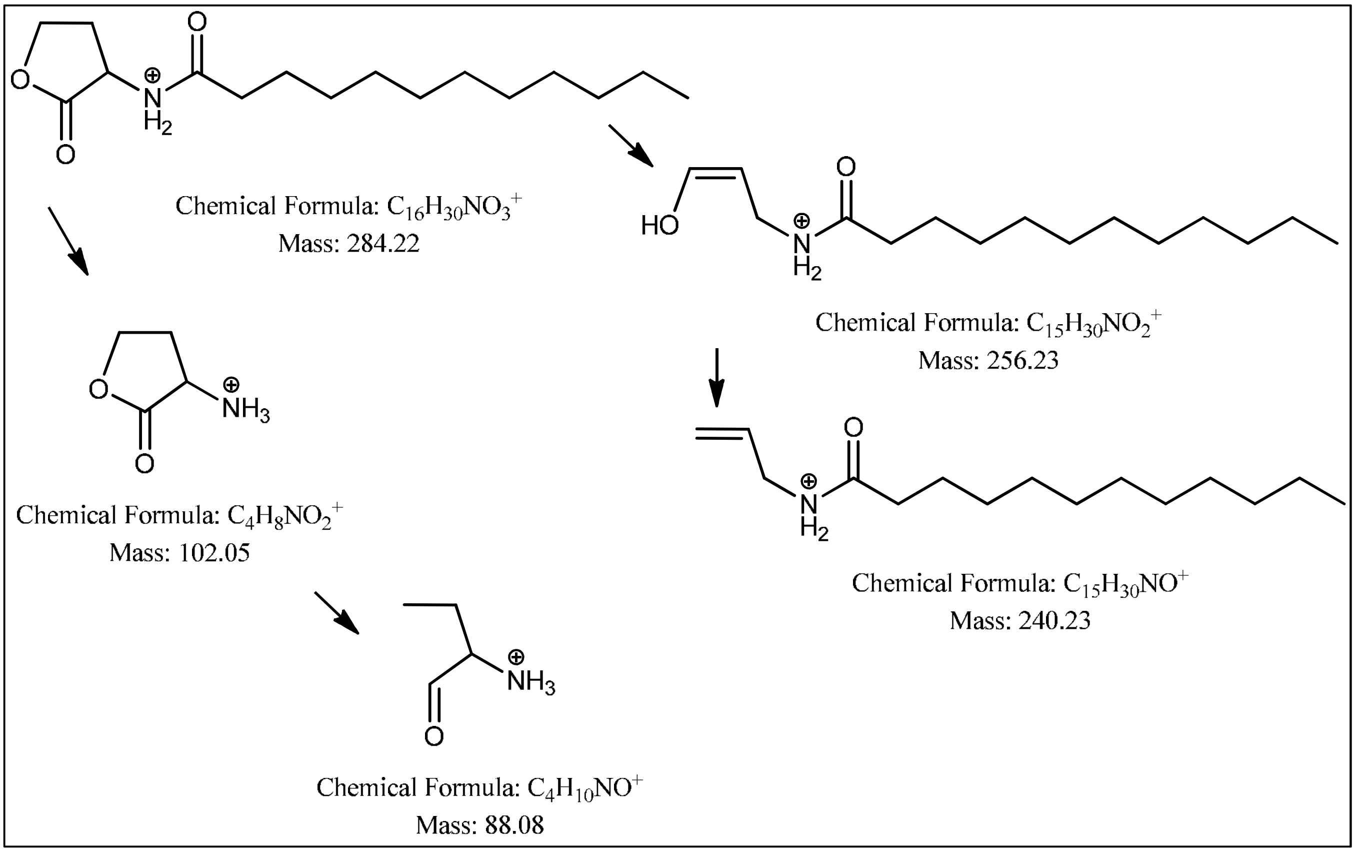
Chemical Formula: $C_{16}H_{30}NO_3^+$
Mass: 284.22
Chemical Formula: $C_{15}H_{30}NO_2^+$
Mass: 256.23
Chemical Formula: $C_4H_8NO_2^+$
Mass: 102.05
Chemical Formula: $C_{15}H_{30}NO^+$
Mass: 240.23
Chemical Formula: $C_4H_{10}NO^+$
Mass: 88.08

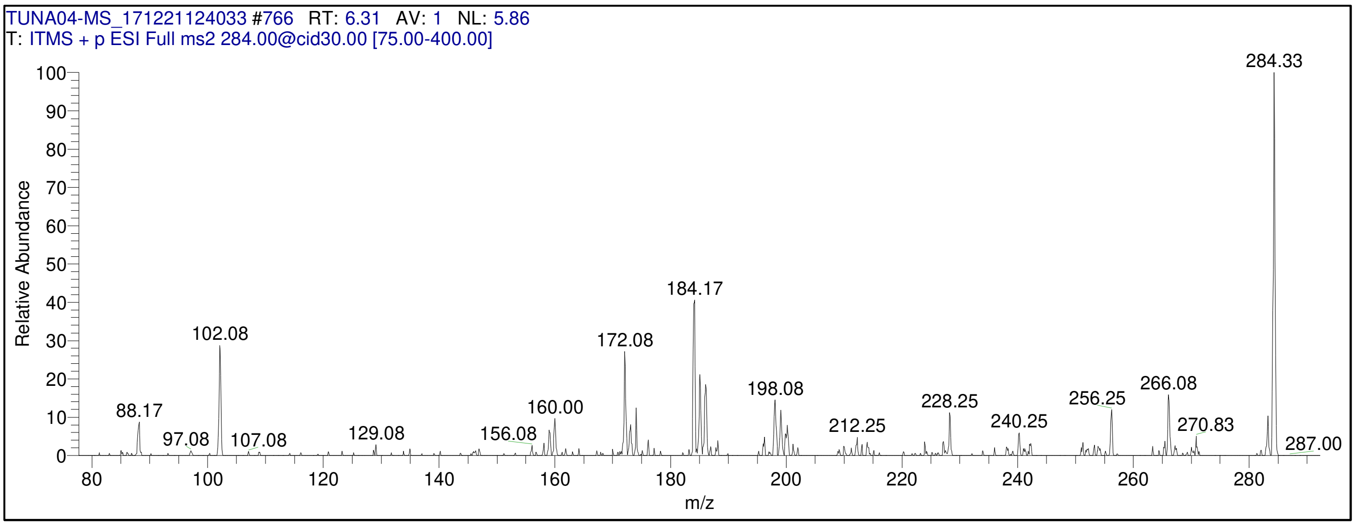
TUNA04-MS_171221124033 #766 RT: 6.31 AV: 1 NL: 5.86
T: ITMS + p ESI Full ms2 284.00@cid30.00 [75.00-400.00]
Relative Abundance
m/z
284.33
184.17
102.08
172.08
160.00
198.08
228.25
256.25
266.08
88.17
97.08
107.08
129.08
156.08
212.25
240.25
270.83
287.00

IV.

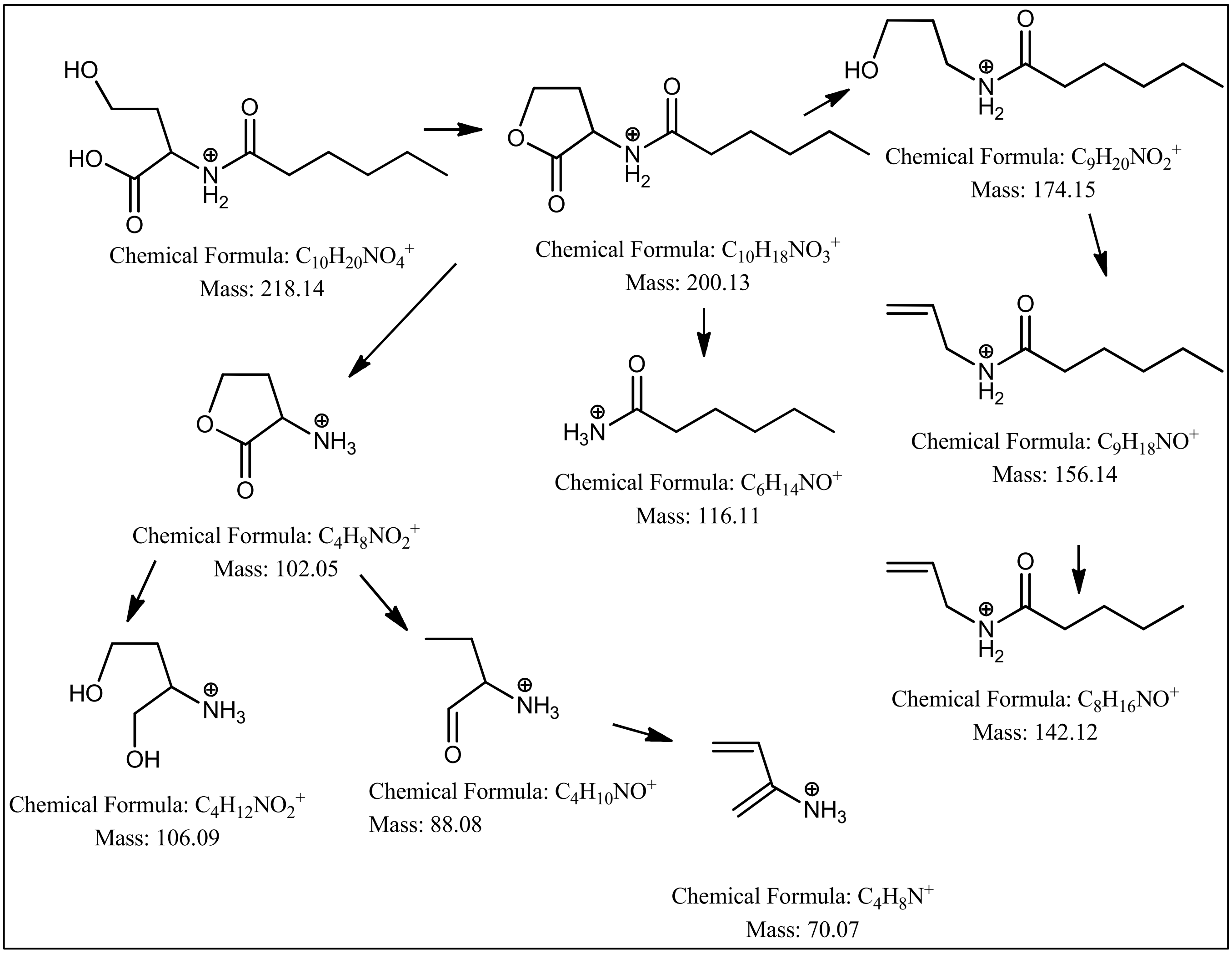
HO
HO
O
N
H2
O
Chemical Formula: C10H20NO4+
Mass: 218.14
O
O
N
H2
O
Chemical Formula: C10H18NO3+
Mass: 200.13
HO
N
H2
O
Chemical Formula: C9H20NO2+
Mass: 174.15
O
NH3
O
Chemical Formula: C4H8NO2+
Mass: 102.05
H3N
O
Chemical Formula: C6H14NO+
Mass: 116.11
N
H2
O
Chemical Formula: C9H18NO+
Mass: 156.14
N
H2
O
Chemical Formula: C8H16NO+
Mass: 142.12
HO
NH3
OH
Chemical Formula: C4H12NO2+
Mass: 106.09
NH3
O
Chemical Formula: C4H10NO+
Mass: 88.08
NH3
Chemical Formula: C4H8N+
Mass: 70.07

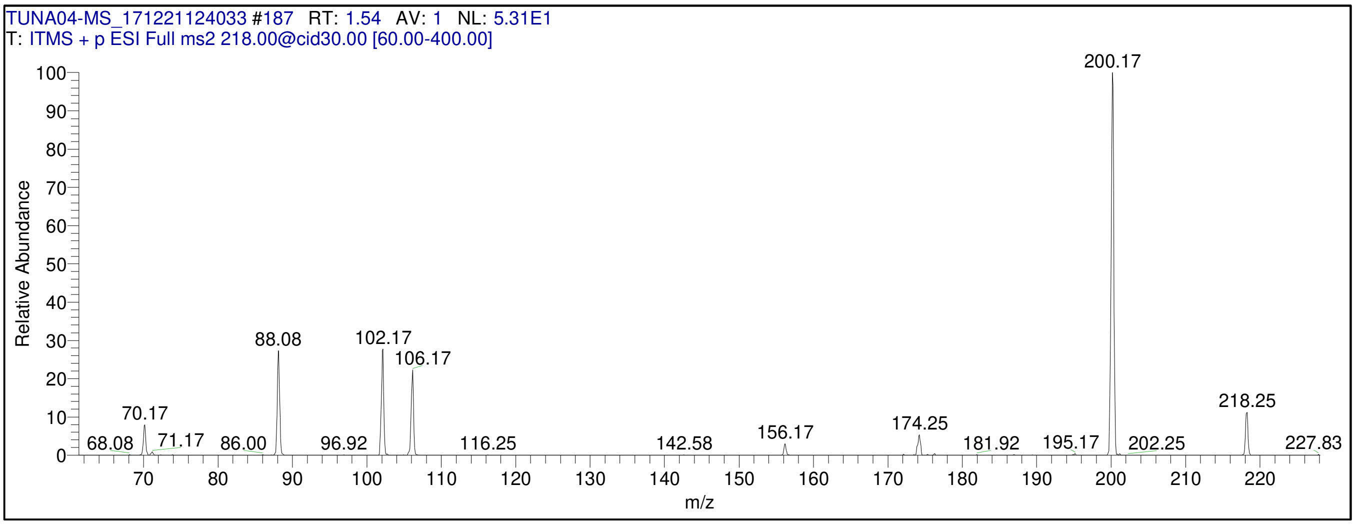
TUNA04-MS_171221124033 #187 RT: 1.54 AV: 1 NL: 5.31E1
T: ITMS + p ESI Full ms2 218.00@cid30.00 [60.00-400.00]
Relative Abundance
m/z
200.17
88.08
102.17
106.17
218.25
70.17
174.25
156.17
68.08
71.17
86.00
96.92
116.25
142.58
181.92
195.17
202.25
227.83

V.

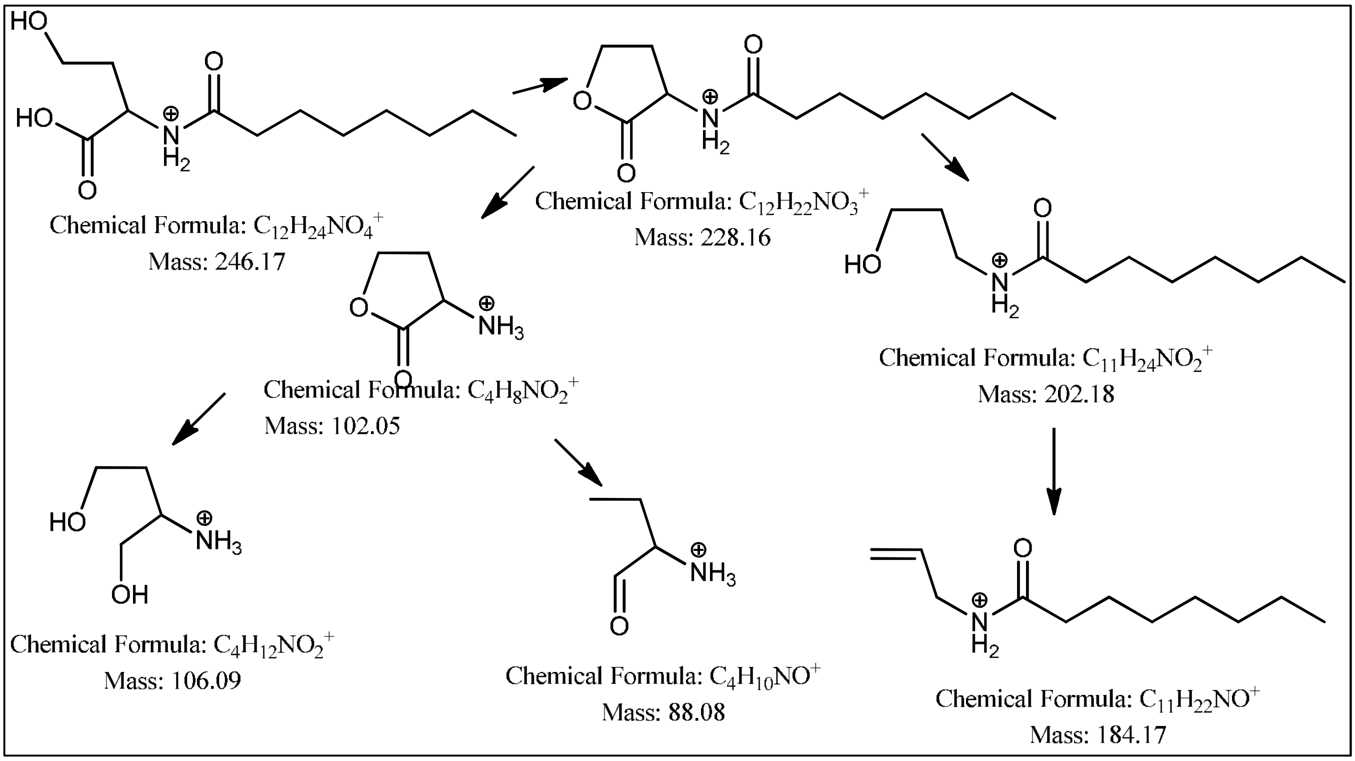

HO
HO
O
O
N
H2
Chemical Formula: C12H24NO4+
Mass: 246.17
O
O
O
N
H2
Chemical Formula: C12H22NO3+
Mass: 228.16
O
O
NH3
Chemical Formula: C4H8NO2+
Mass: 102.05
HO
O
N
H2
Chemical Formula: C11H24NO2+
Mass: 202.18
HO
NH3
OH
Chemical Formula: C4H12NO2+
Mass: 106.09
NH3
O
Chemical Formula: C4H10NO+
Mass: 88.08
O
N
H2
Chemical Formula: C11H22NO+
Mass: 184.17

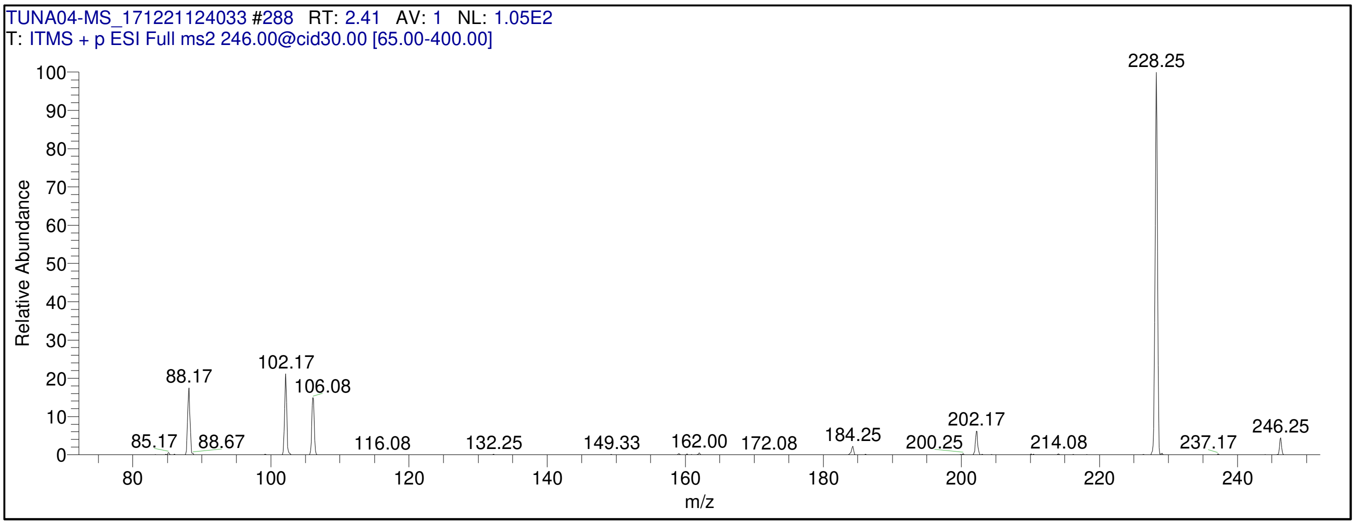

TUNA04-MS_171221124033 #288 RT: 2.41 AV: 1 NL: 1.05E2
T: ITMS + p ESI Full ms2 246.00@cid30.00 [65.00-400.00]
Relative Abundance
m/z
228.25
88.17
102.17
106.08
85.17
88.67
116.08
132.25
149.33
162.00
172.08
184.25
200.25
202.17
214.08
237.17
246.25

VI.

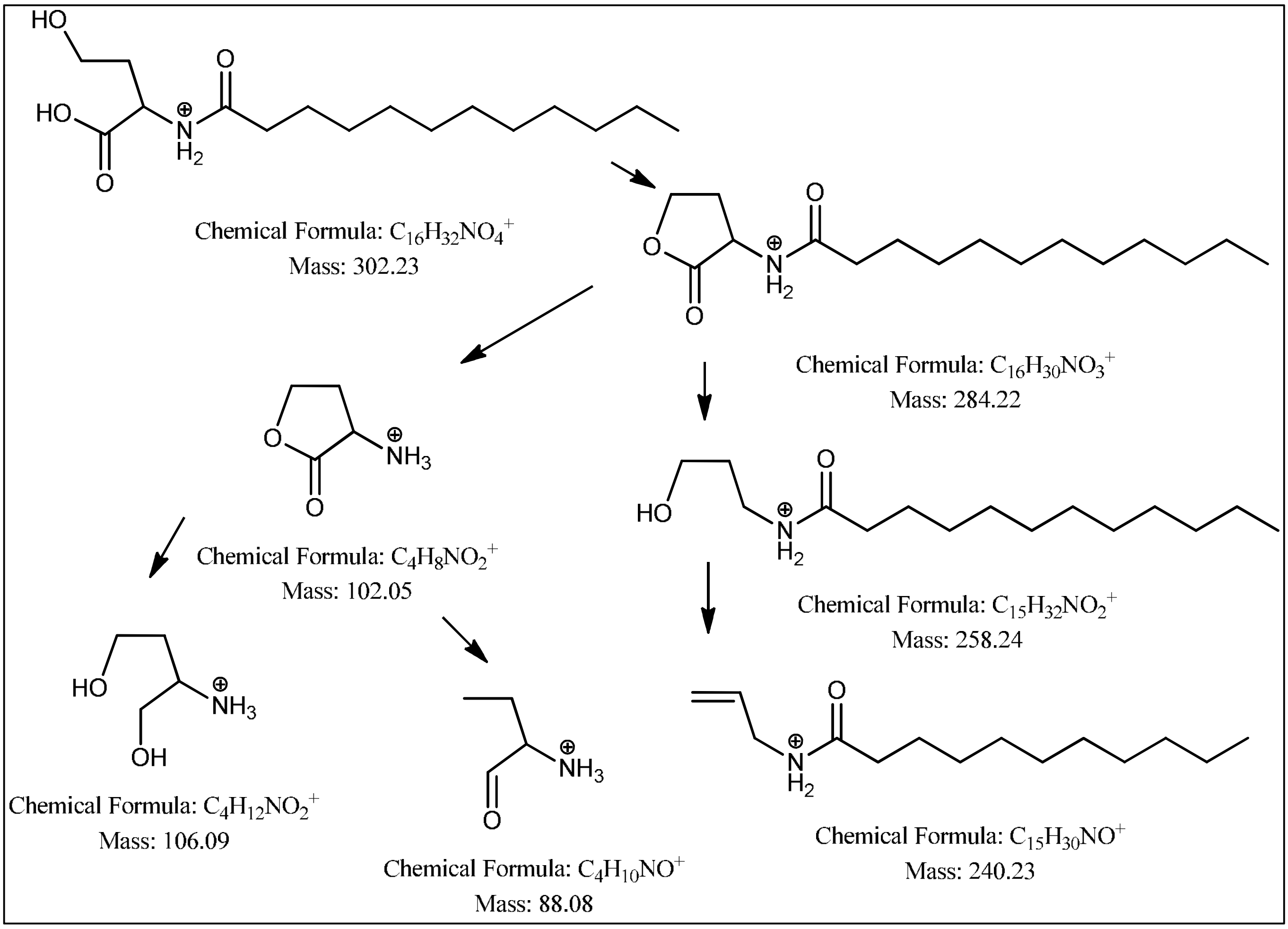

Chemical Formula: $C_{16}H_{32}NO_4^+$
Mass: 302.23
Chemical Formula: $C_{16}H_{30}NO_3^+$
Mass: 284.22
Chemical Formula: $C_4H_8NO_2^+$
Mass: 102.05
Chemical Formula: $C_{15}H_{32}NO_2^+$
Mass: 258.24
Chemical Formula: $C_4H_{12}NO_2^+$
Mass: 106.09
Chemical Formula: $C_4H_{10}NO^+$
Mass: 88.08
Chemical Formula: $C_{15}H_{30}NO^+$
Mass: 240.23

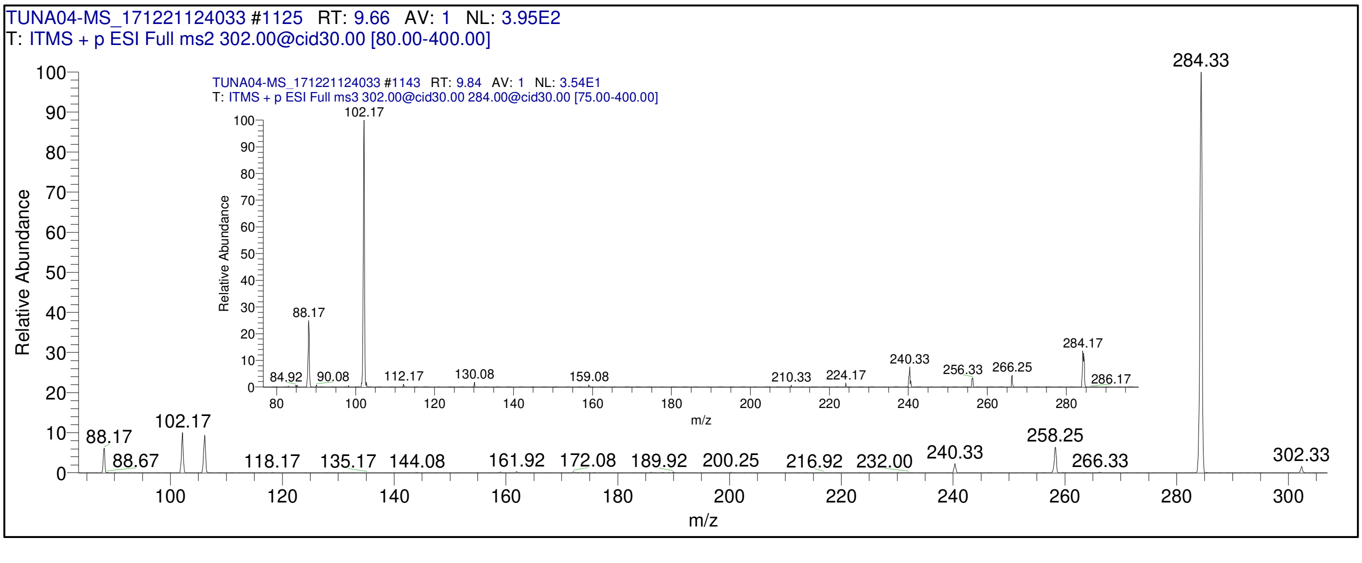

TUNA04-MS_171221124033 #1125 RT: 9.66 AV: 1 NL: 3.95E2
T: ITMS + p ESI Full ms2 302.00@cid30.00 [80.00-400.00]
TUNA04-MS_171221124033 #1143 RT: 9.84 AV: 1 NL: 3.54E1
T: ITMS + p ESI Full ms3 302.00@cid30.00 284.00@cid30.00 [75.00-400.00]
Relative Abundance
m/z

VII.

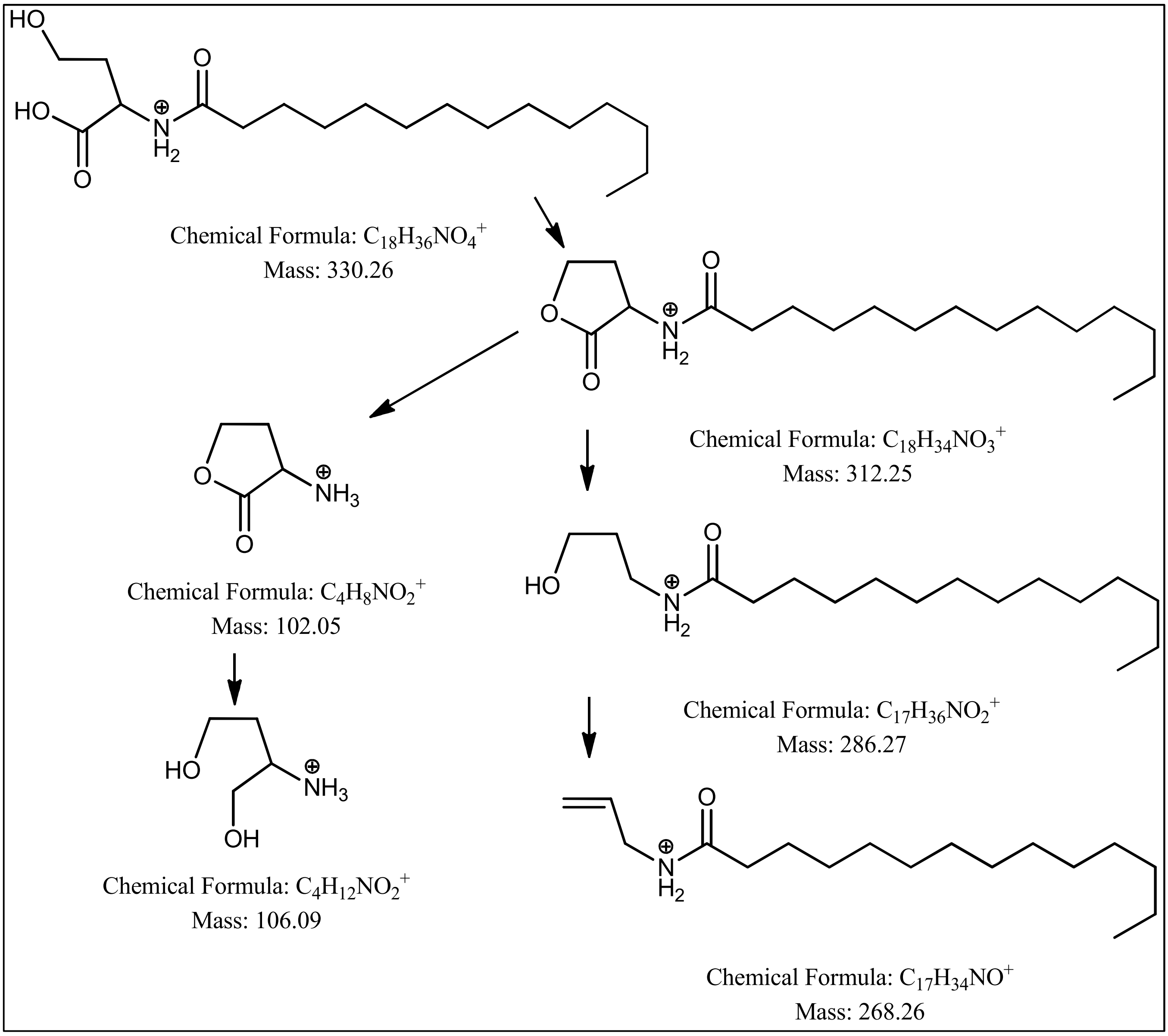
HO
HO
O
O
N
H2
Chemical Formula: $C_{18}H_{36}NO_4^+$
Mass: 330.26
O
O
O
N
H2
Chemical Formula: $C_{18}H_{34}NO_3^+$
Mass: 312.25
O
O
NH3
Chemical Formula: $C_4H_8NO_2^+$
Mass: 102.05
HO
O
N
H2
Chemical Formula: $C_{17}H_{36}NO_2^+$
Mass: 286.27
HO
NH3
OH
Chemical Formula: $C_4H_{12}NO_2^+$
Mass: 106.09
O
N
H2
Chemical Formula: $C_{17}H_{34}NO^+$
Mass: 268.26

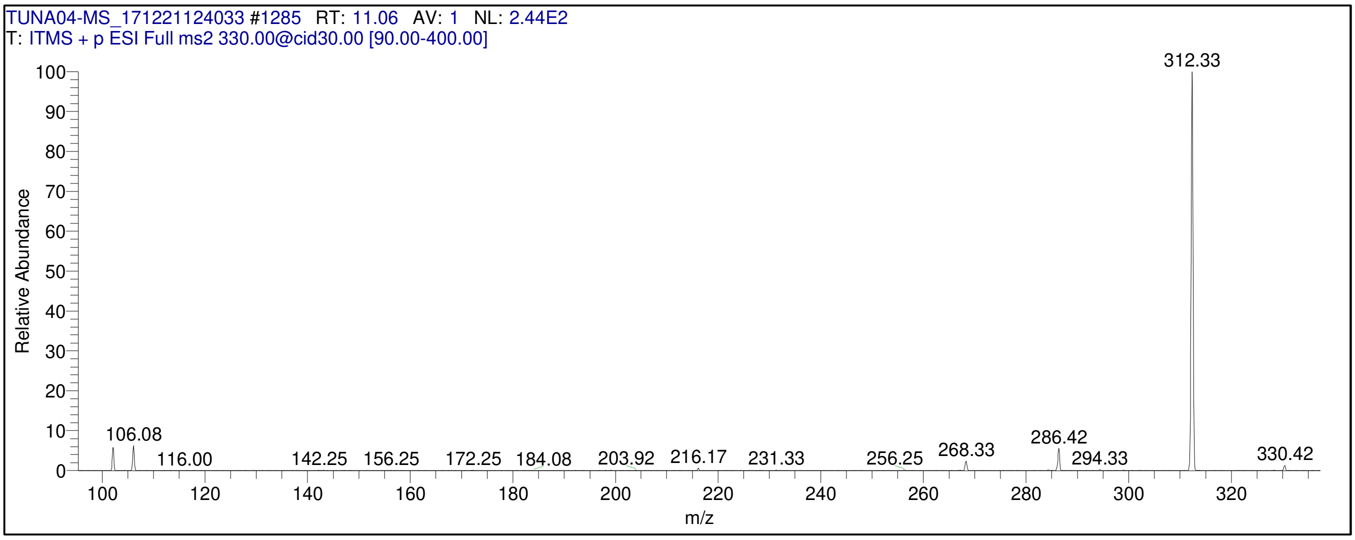
TUNA04-MS_171221124033 #1285 RT: 11.06 AV: 1 NL: 2.44E2
T: ITMS + p ESI Full ms2 330.00@cid30.00 [90.00-400.00]
Relative Abundance
m/z
312.33
106.08
116.00
142.25
156.25
172.25
184.08
203.92
216.17
231.33
256.25
268.33
286.42
294.33
330.42

VIII.

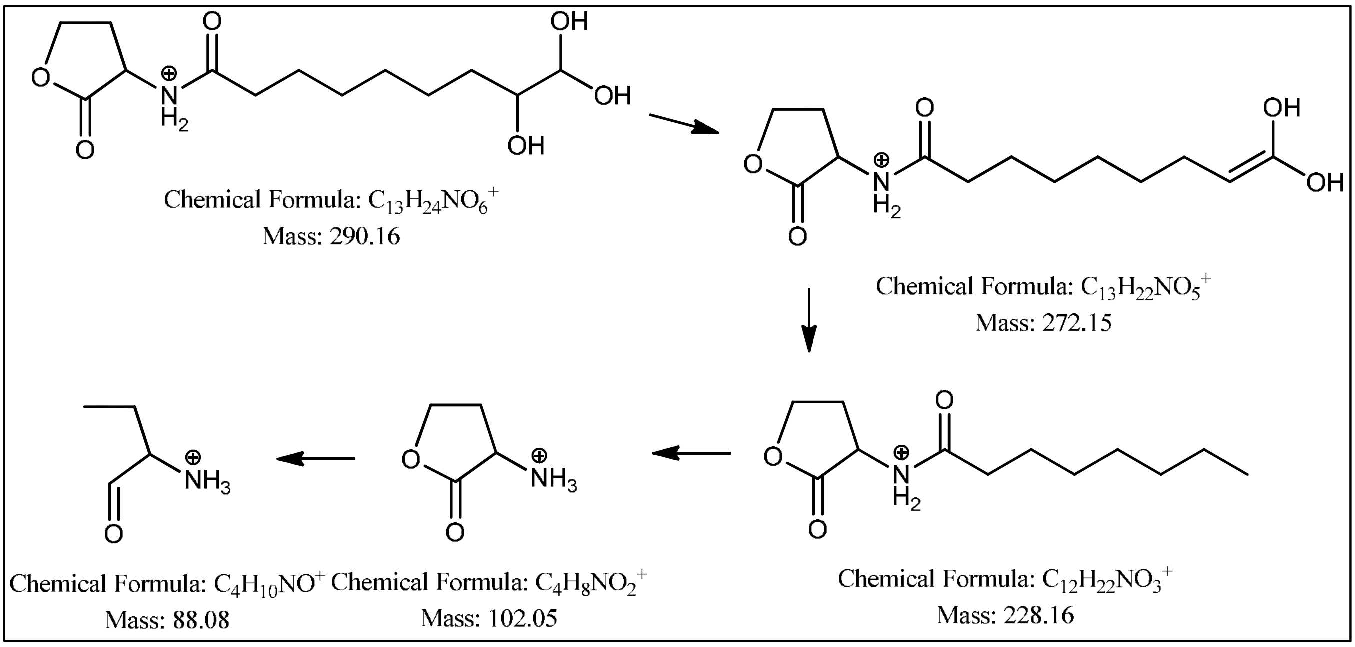
Chemical Formula: $C_{13}H_{24}NO_6^+$
Mass: 290.16
Chemical Formula: $C_{13}H_{22}NO_5^+$
Mass: 272.15
Chemical Formula: $C_{12}H_{22}NO_3^+$
Mass: 228.16
Chemical Formula: $C_4H_8NO_2^+$
Mass: 102.05
Chemical Formula: $C_4H_{10}NO^+$
Mass: 88.08

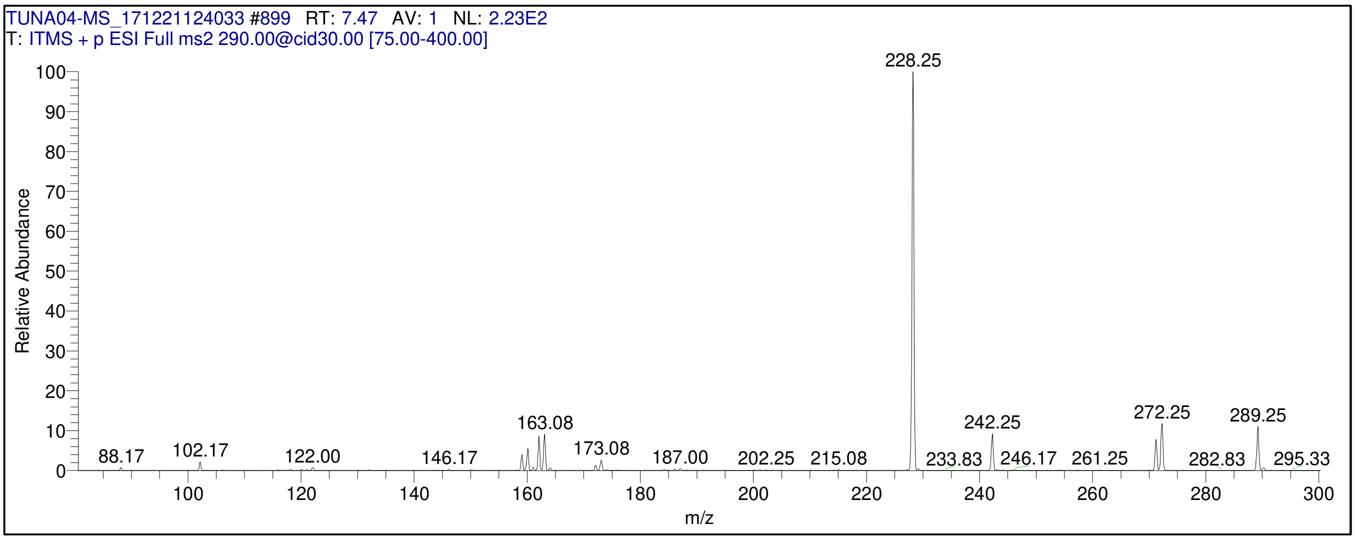
TUNA04-MS_171221124033 #899 RT: 7.47 AV: 1 NL: 2.23E2
T: ITMS + p ESI Full ms2 290.00@cid30.00 [75.00-400.00]
Relative Abundance
m/z
228.25
88.17
102.17
122.00
146.17
163.08
173.08
187.00
202.25
215.08
233.83
242.25
246.17
261.25
272.25
282.83
289.25
295.33

IX.

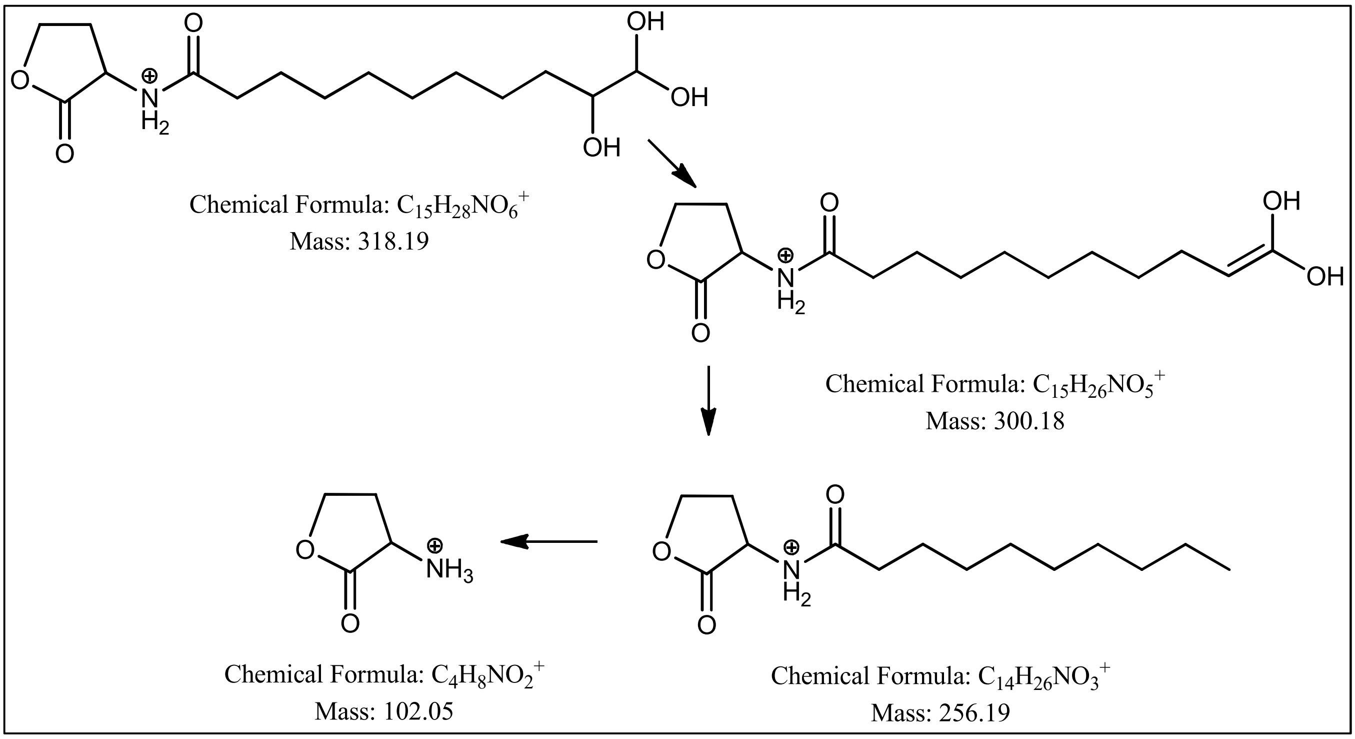

Chemical Formula: $C_{15}H_{28}NO_6^+$
Mass: 318.19
Chemical Formula: $C_{15}H_{26}NO_5^+$
Mass: 300.18
Chemical Formula: $C_4H_8NO_2^+$
Mass: 102.05
Chemical Formula: $C_{14}H_{26}NO_3^+$
Mass: 256.19

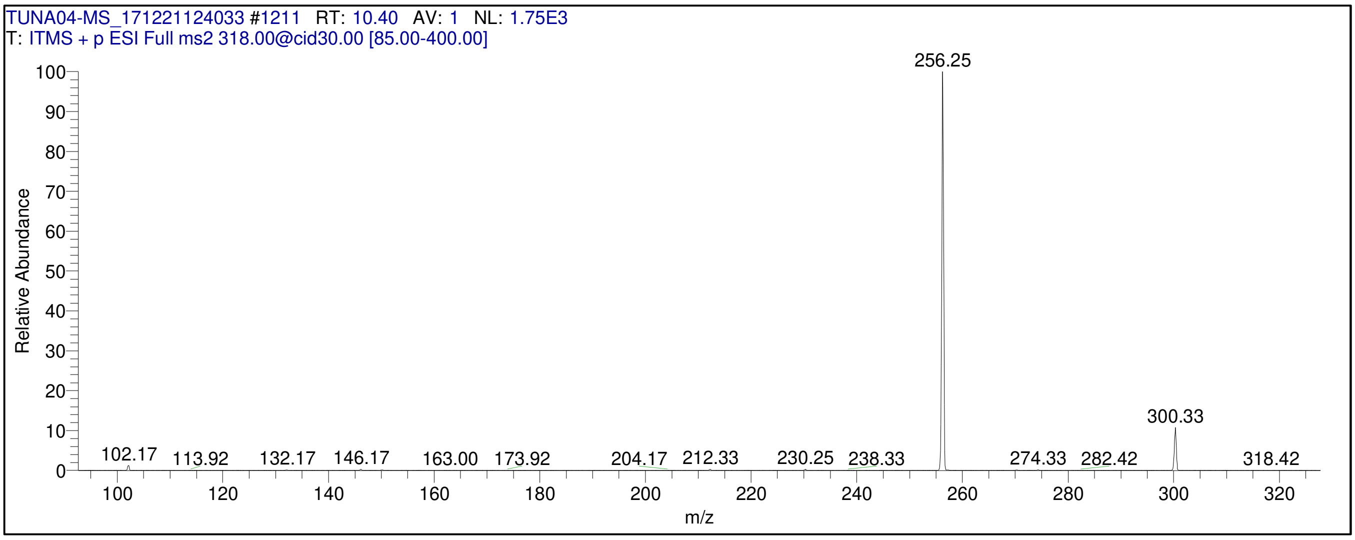

TUNA04-MS_171221124033 #1211 RT: 10.40 AV: 1 NL: 1.75E3
T: ITMS + p ESI Full ms2 318.00@cid30.00 [85.00-400.00]
Relative Abundance
m/z
256.25
300.33
102.17
113.92
132.17
146.17
163.00
173.92
204.17
212.33
230.25
238.33
274.33
282.42
318.42

X.

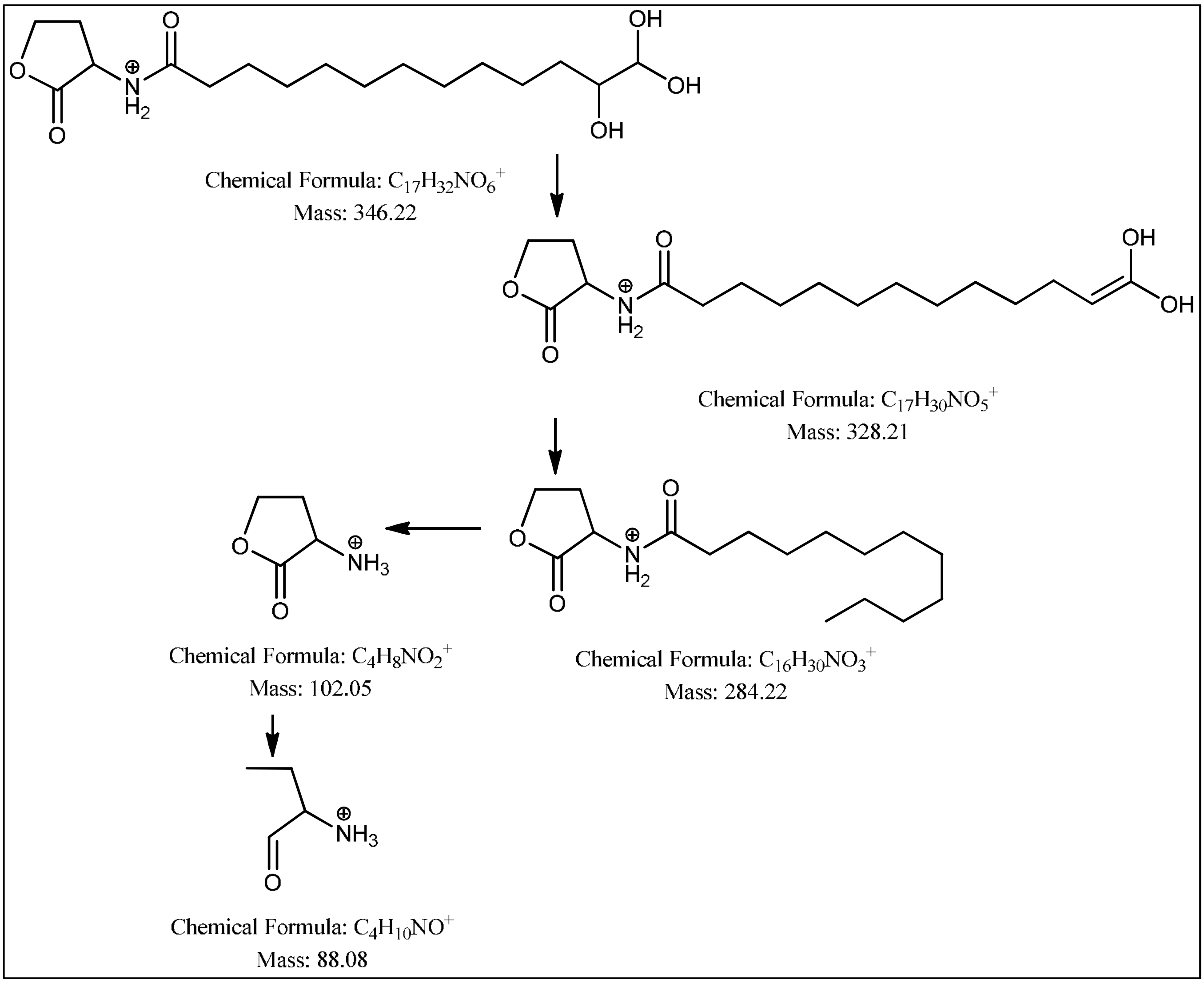
Chemical Formula: C17H32NO6+
Mass: 346.22
Chemical Formula: C17H30NO5+
Mass: 328.21
Chemical Formula: C4H8NO2+
Mass: 102.05
Chemical Formula: C16H30NO3+
Mass: 284.22
Chemical Formula: C4H10NO+
Mass: 88.08

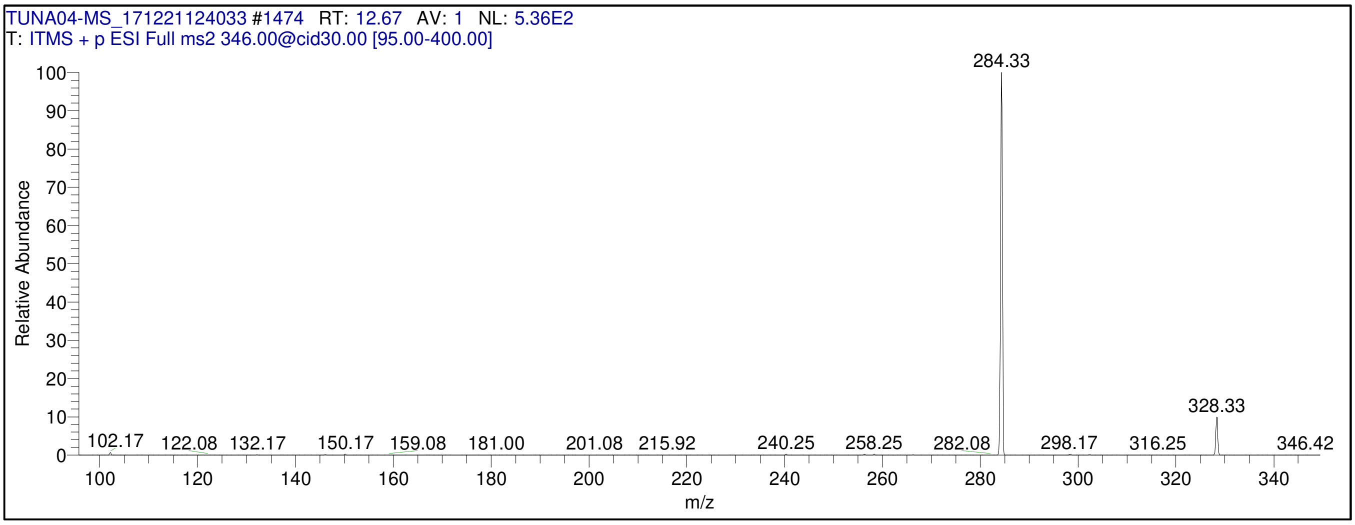
TUNA04-MS_171221124033 #1474 RT: 12.67 AV: 1 NL: 5.36E2
T: ITMS + p ESI Full ms2 346.00@cid30.00 [95.00-400.00]
284.33
328.33
102.17 122.08 132.17 150.17 159.08 181.00 201.08 215.92 240.25 258.25 282.08 298.17 316.25 346.42
Relative Abundance
m/z

XI.

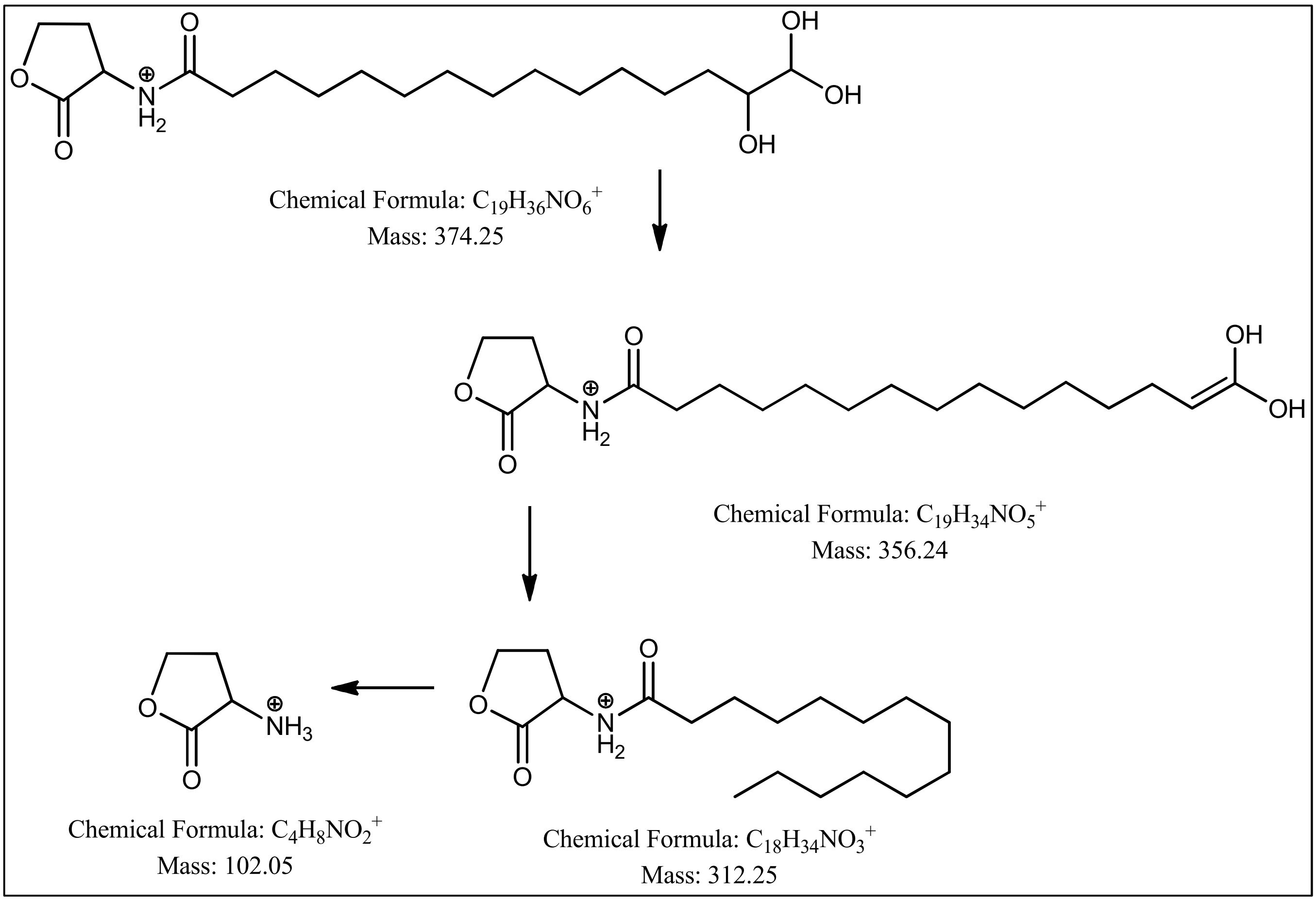

Chemical Formula: $C_{19}H_{36}NO_6^+$
Mass: 374.25
Chemical Formula: $C_{19}H_{34}NO_5^+$
Mass: 356.24
Chemical Formula: $C_4H_8NO_2^+$
Mass: 102.05
Chemical Formula: $C_{18}H_{34}NO_3^+$
Mass: 312.25

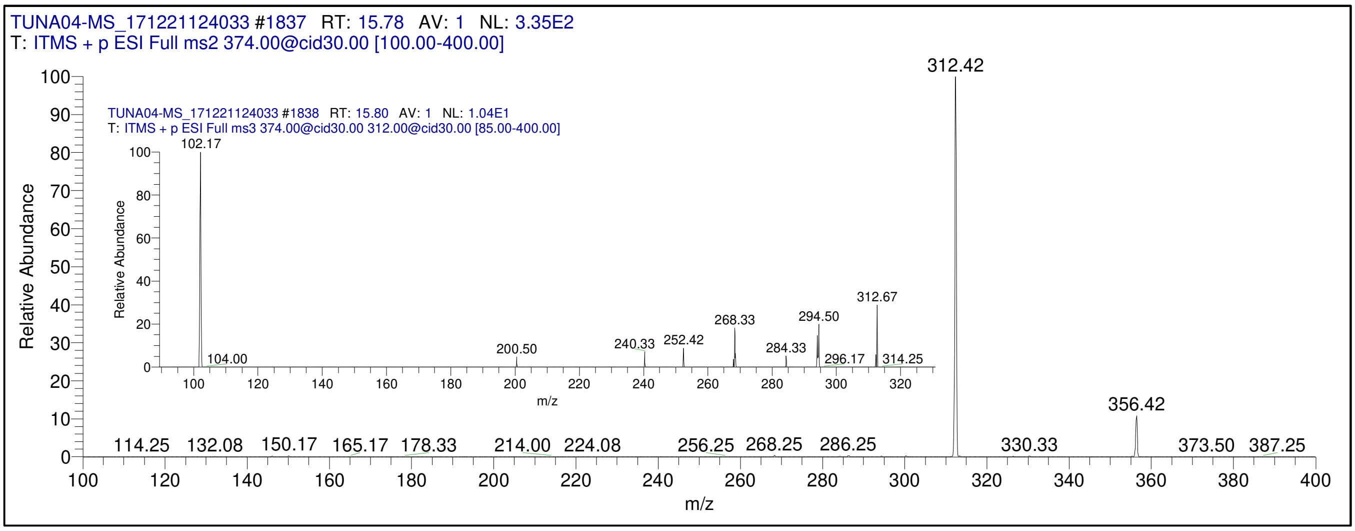

TUNA04-MS_171221124033 #1837 RT: 15.78 AV: 1 NL: 3.35E2
T: ITMS + p ESI Full ms2 374.00@cid30.00 [100.00-400.00]
TUNA04-MS_171221124033 #1838 RT: 15.80 AV: 1 NL: 1.04E1
T: ITMS + p ESI Full ms3 374.00@cid30.00 312.00@cid30.00 [85.00-400.00]
Relative Abundance
m/z
312.42
356.42
102.17
312.67
294.50
268.33

Fig. S2 Putative structures of HAQs molecules fragment ion produced by CID of the **I)** *m/z* 244, **II)** *m/z* 258, **III)** *m/z* 258, **IV)** *m/z* 272, **V)** *m/z* 272, **VI)** *m/z* 286, **VII)** *m/z* 286, **VIII) *m/z* 288** and **IX)** *m/z* 260 [M+H] $^+$ at positive ion mode.

I.

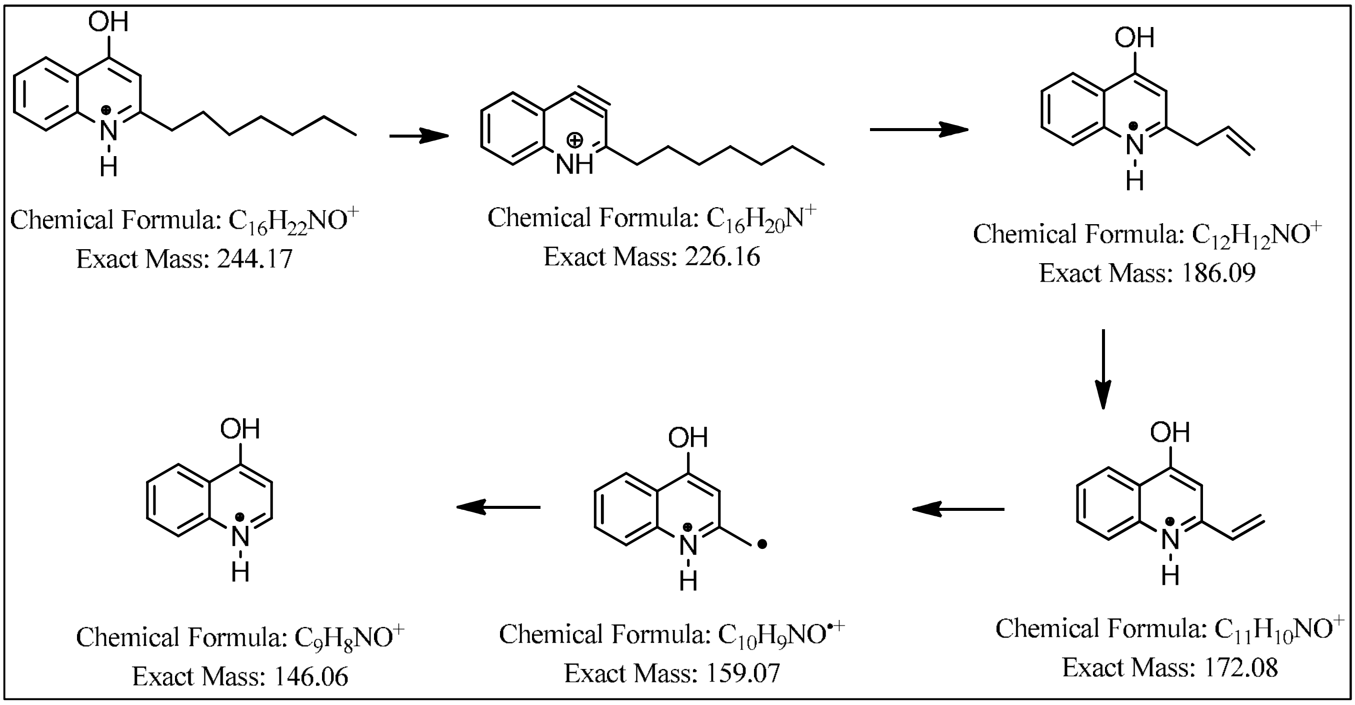

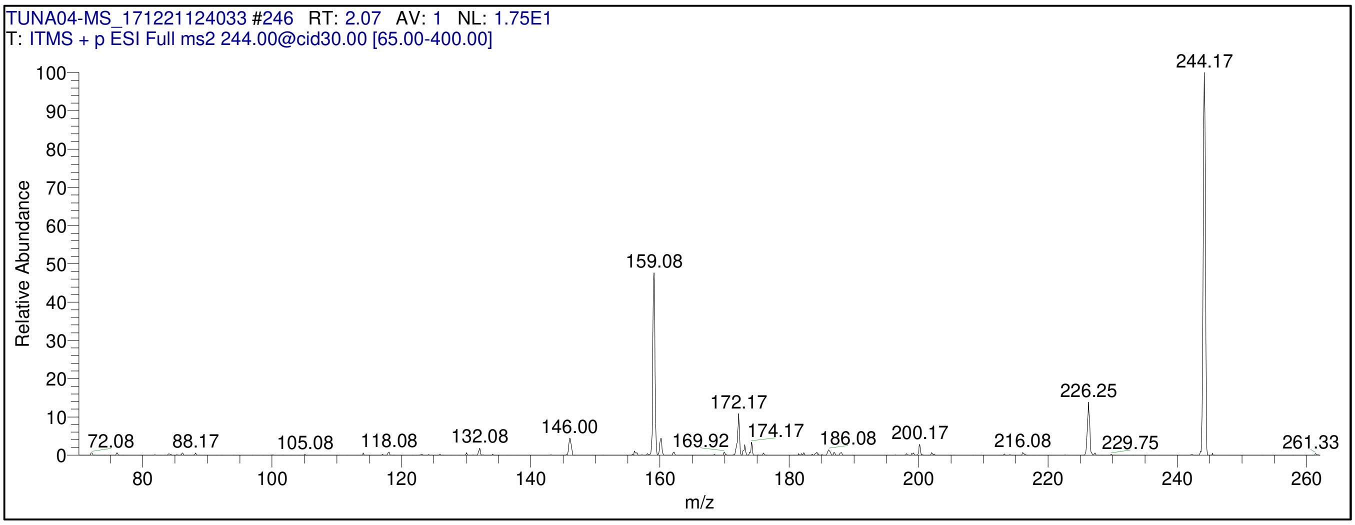

II.

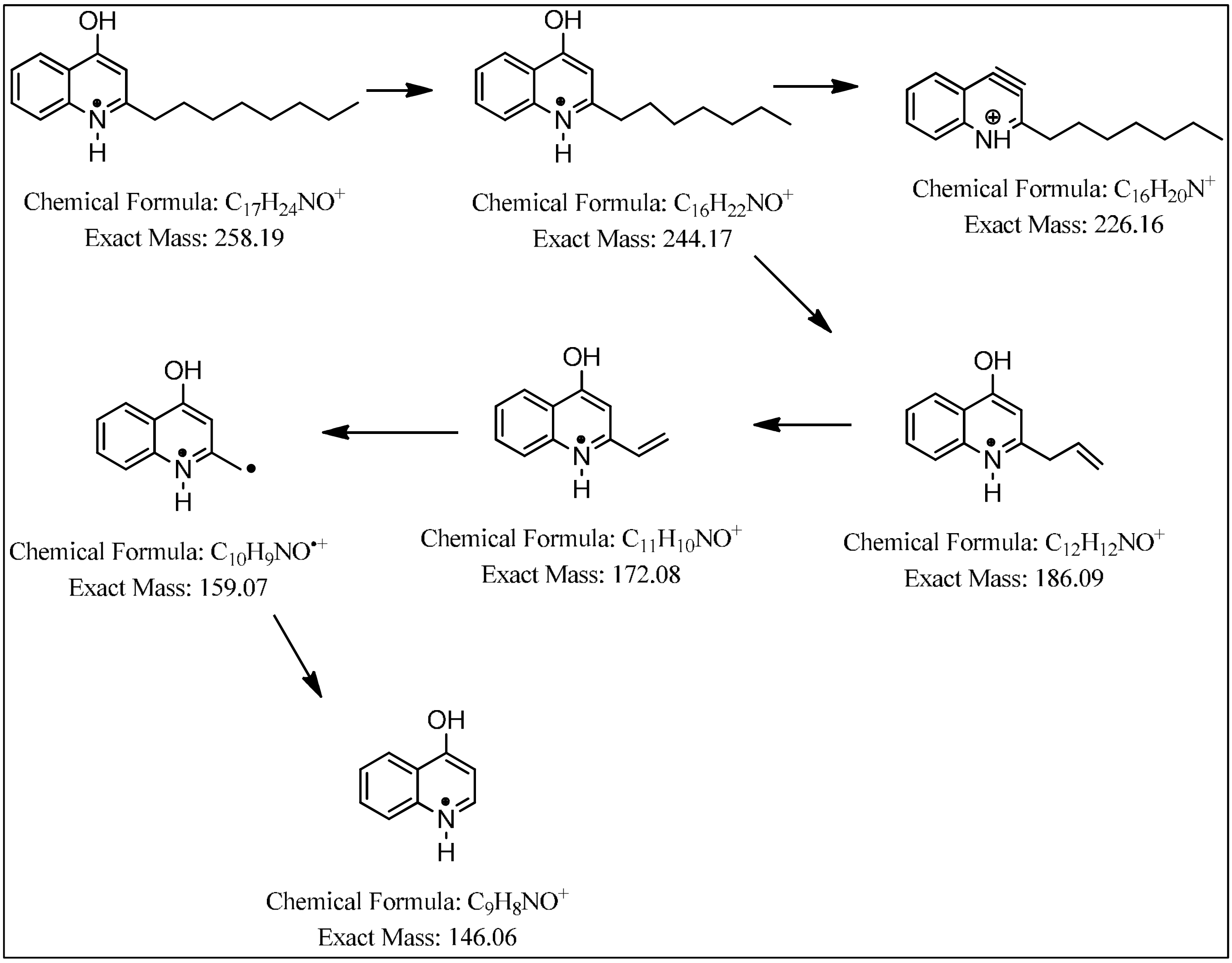
OH
N
H
Chemical Formula: $C_{17}H_{24}NO^+$
Exact Mass: 258.19
Chemical Formula: $C_{16}H_{22}NO^+$
Exact Mass: 244.17
NH
Chemical Formula: $C_{16}H_{20}N^+$
Exact Mass: 226.16
Chemical Formula: $C_{12}H_{12}NO^+$
Exact Mass: 186.09
Chemical Formula: $C_{11}H_{10}NO^+$
Exact Mass: 172.08
Chemical Formula: $C_{10}H_9NO^{\bullet+}$
Exact Mass: 159.07
Chemical Formula: $C_9H_8NO^+$
Exact Mass: 146.06

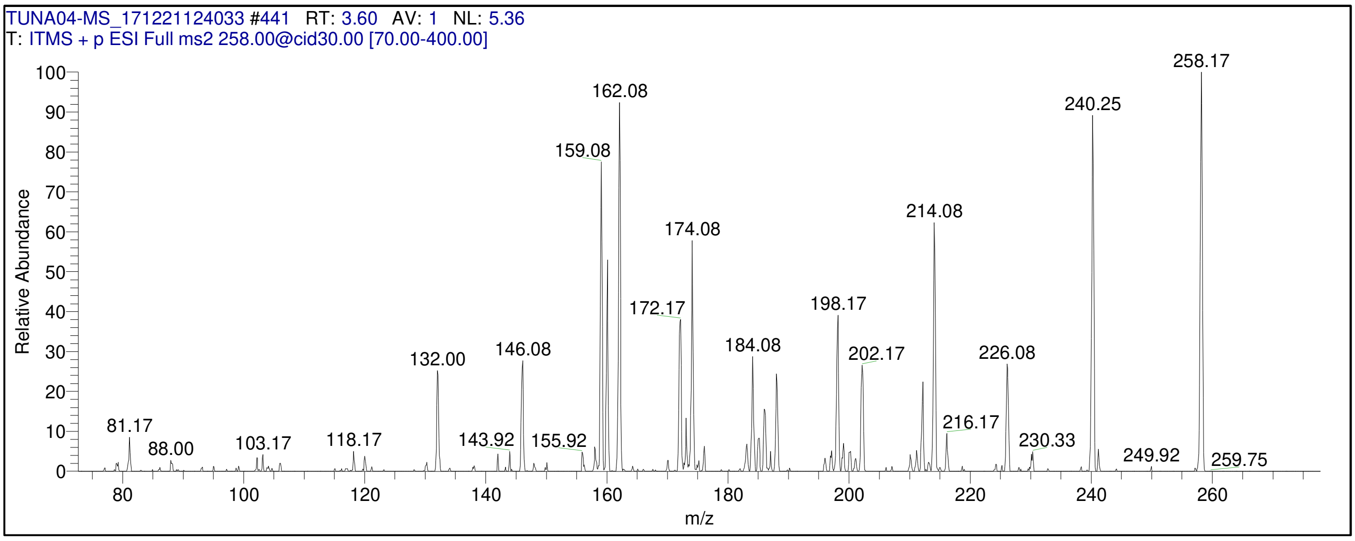
TUNA04-MS_171221124033 #441 RT: 3.60 AV: 1 NL: 5.36
T: ITMS + p ESI Full ms2 258.00@cid30.00 [70.00-400.00]
Relative Abundance
m/z
258.17
162.08
240.25
159.08
174.08
214.08
172.17
198.17
184.08
202.17
226.08
132.00
146.08
216.17
230.33
81.17
88.00
103.17
118.17
143.92
155.92
249.92
259.75

III.

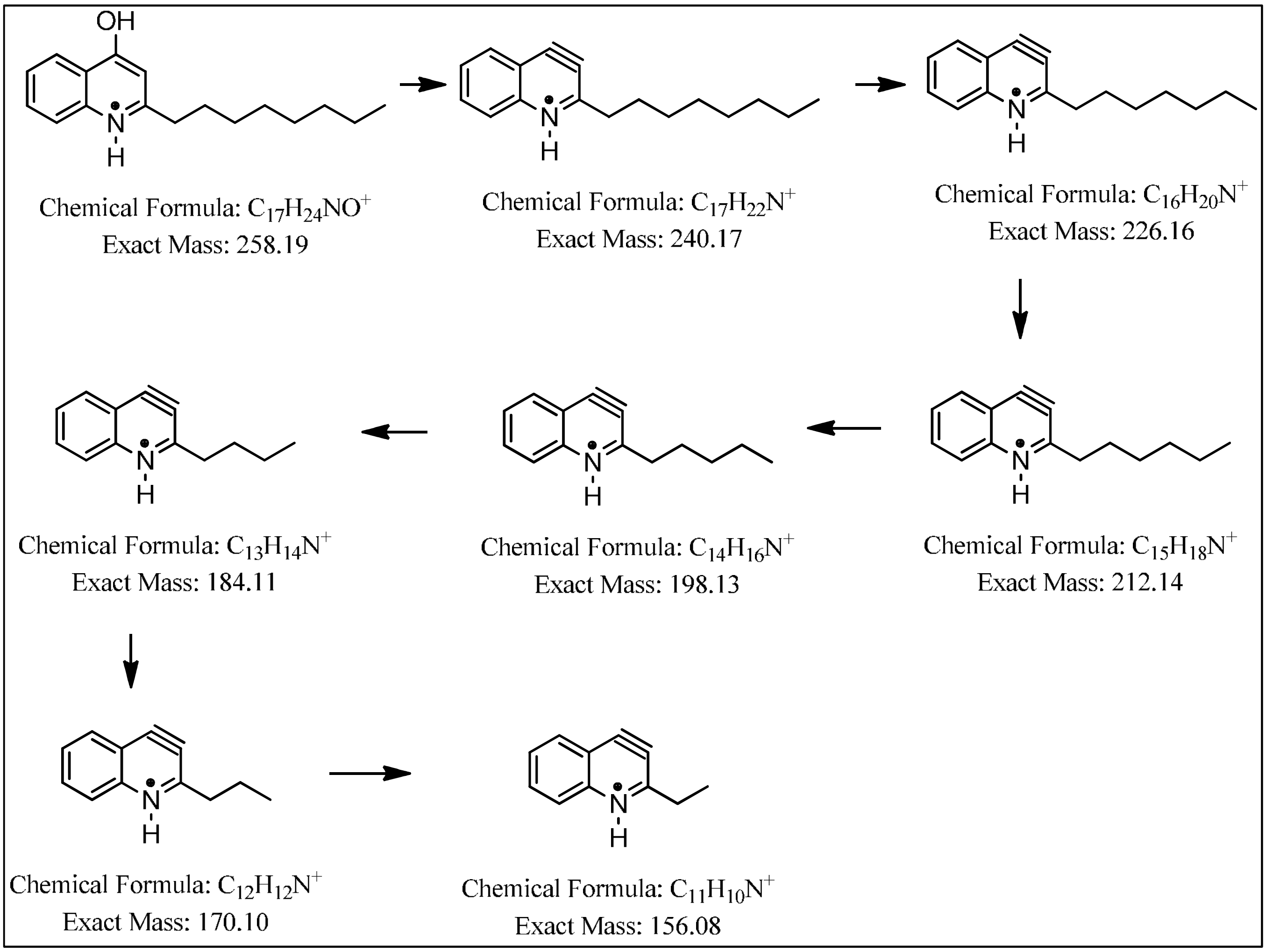
OH
N
H
Chemical Formula: C17H24NO+
Exact Mass: 258.19
Chemical Formula: C17H22N+
Exact Mass: 240.17
Chemical Formula: C16H20N+
Exact Mass: 226.16
Chemical Formula: C15H18N+
Exact Mass: 212.14
Chemical Formula: C14H16N+
Exact Mass: 198.13
Chemical Formula: C13H14N+
Exact Mass: 184.11
Chemical Formula: C12H12N+
Exact Mass: 170.10
Chemical Formula: C11H10N+
Exact Mass: 156.08

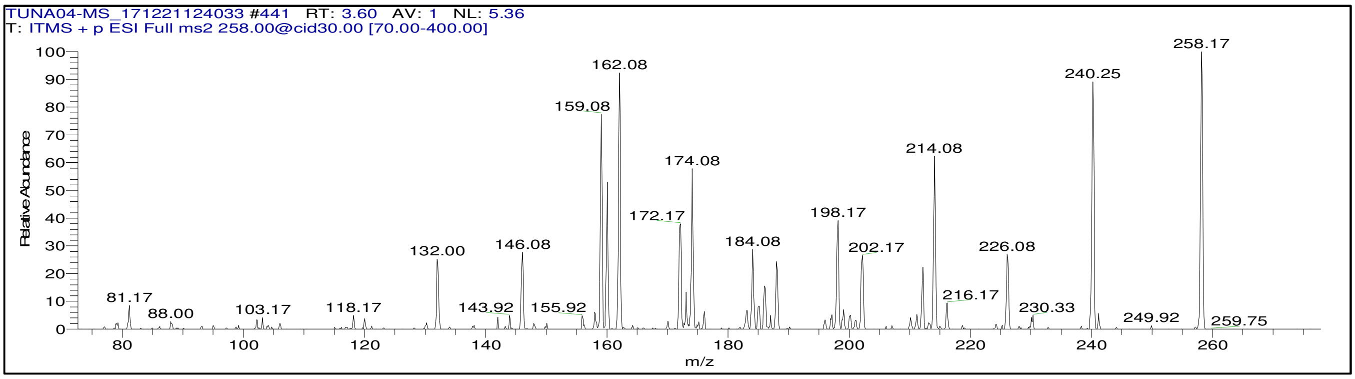
TUNA04-MS_171221124033 #441 RT: 3.60 AV: 1 NL: 5.36
T: ITMS + p ESI Full ms2 258.00@cid30.00 [70.00-400.00]
Relative Abundance
m/z
258.17
240.25
162.08
159.08
214.08
174.08
172.17
198.17
184.08
202.17
226.08
132.00
146.08
216.17
81.17
88.00
103.17
118.17
143.92
155.92
230.33
249.92
259.75

IV.

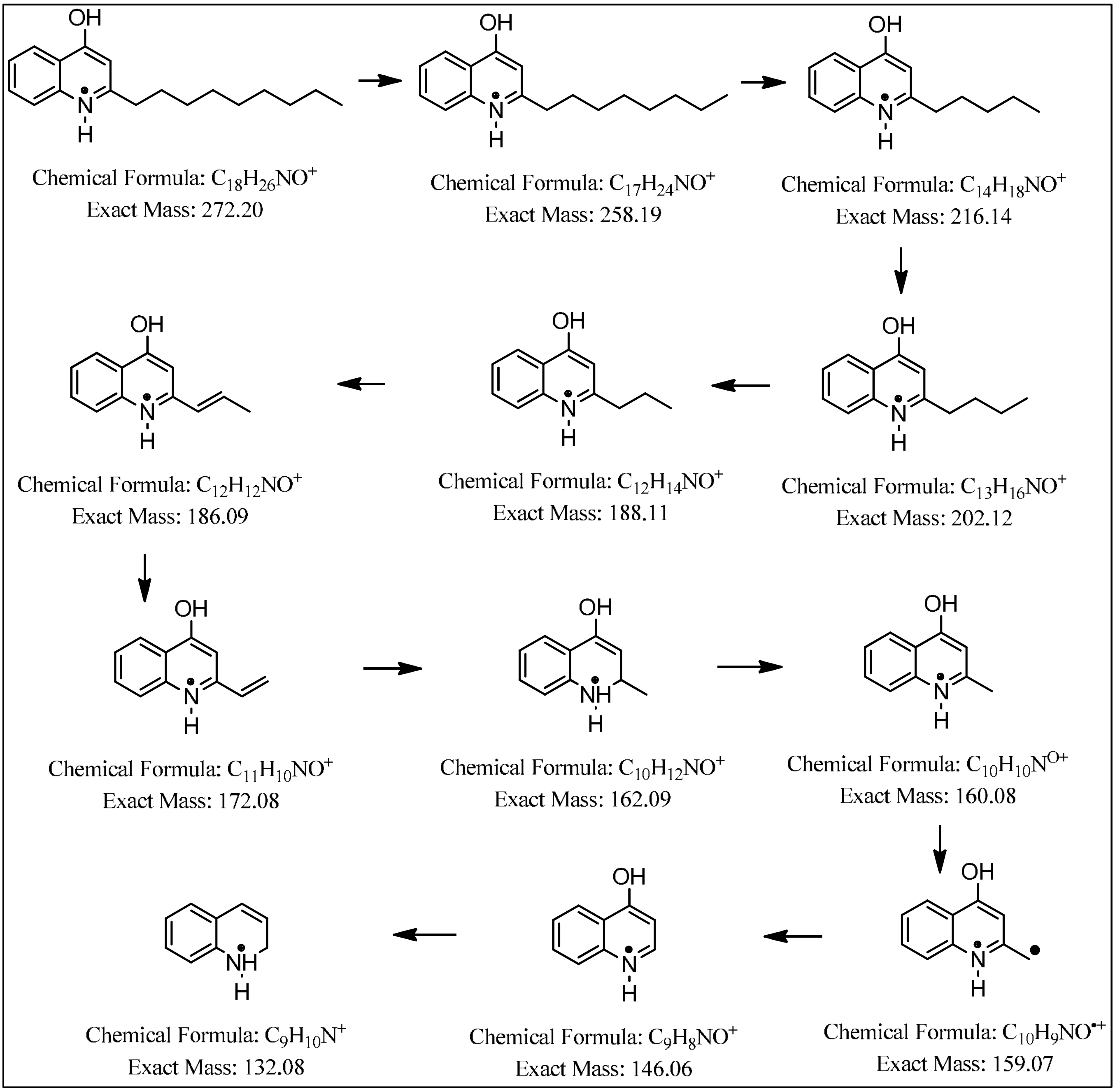
OH
N
H
Chemical Formula: $C_{18}H_{26}NO^+$
Exact Mass: 272.20
Chemical Formula: $C_{17}H_{24}NO^+$
Exact Mass: 258.19
Chemical Formula: $C_{14}H_{18}NO^+$
Exact Mass: 216.14
Chemical Formula: $C_{13}H_{16}NO^+$
Exact Mass: 202.12
Chemical Formula: $C_{12}H_{14}NO^+$
Exact Mass: 188.11
Chemical Formula: $C_{12}H_{12}NO^+$
Exact Mass: 186.09
Chemical Formula: $C_{11}H_{10}NO^+$
Exact Mass: 172.08
Chemical Formula: $C_{10}H_{12}NO^+$
Exact Mass: 162.09
Chemical Formula: $C_{10}H_{10}N^{O+}$
Exact Mass: 160.08
Chemical Formula: $C_{10}H_9NO^{\bullet+}$
Exact Mass: 159.07
Chemical Formula: $C_9H_8NO^+$
Exact Mass: 146.06
Chemical Formula: $C_9H_{10}N^+$
Exact Mass: 132.08

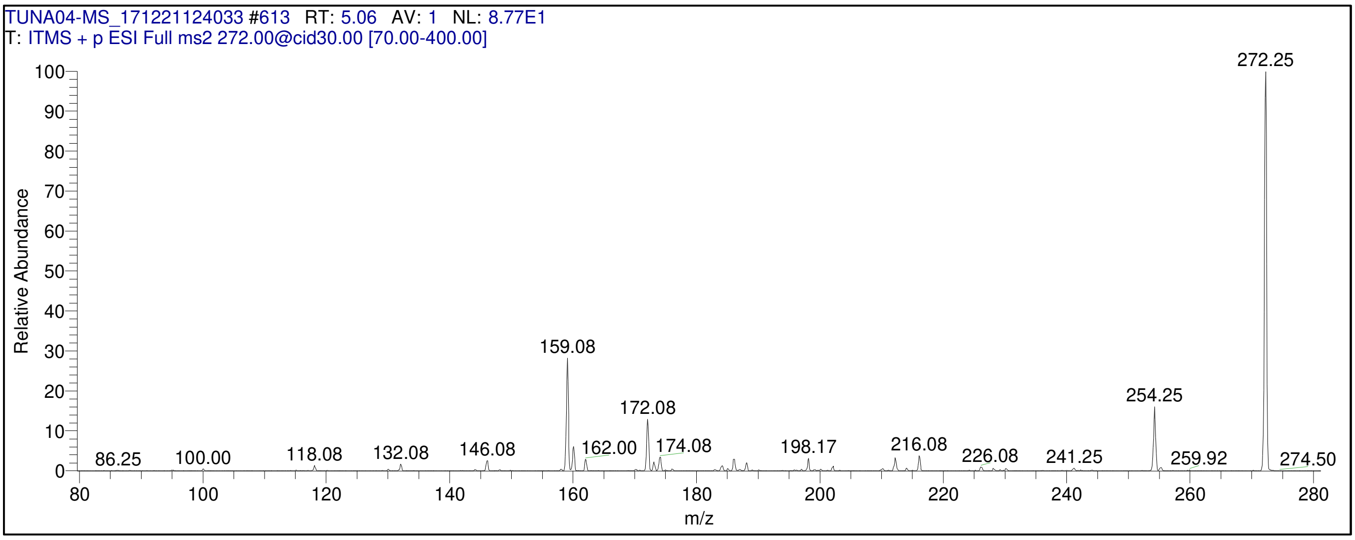
TUNA04-MS_171221124033 #613 RT: 5.06 AV: 1 NL: 8.77E1
T: ITMS + p ESI Full ms2 272.00@cid30.00 [70.00-400.00]
Relative Abundance
m/z
272.25
254.25
159.08
172.08
86.25
100.00
118.08
132.08
146.08
162.00
174.08
198.17
216.08
226.08
241.25
259.92
274.50

V.

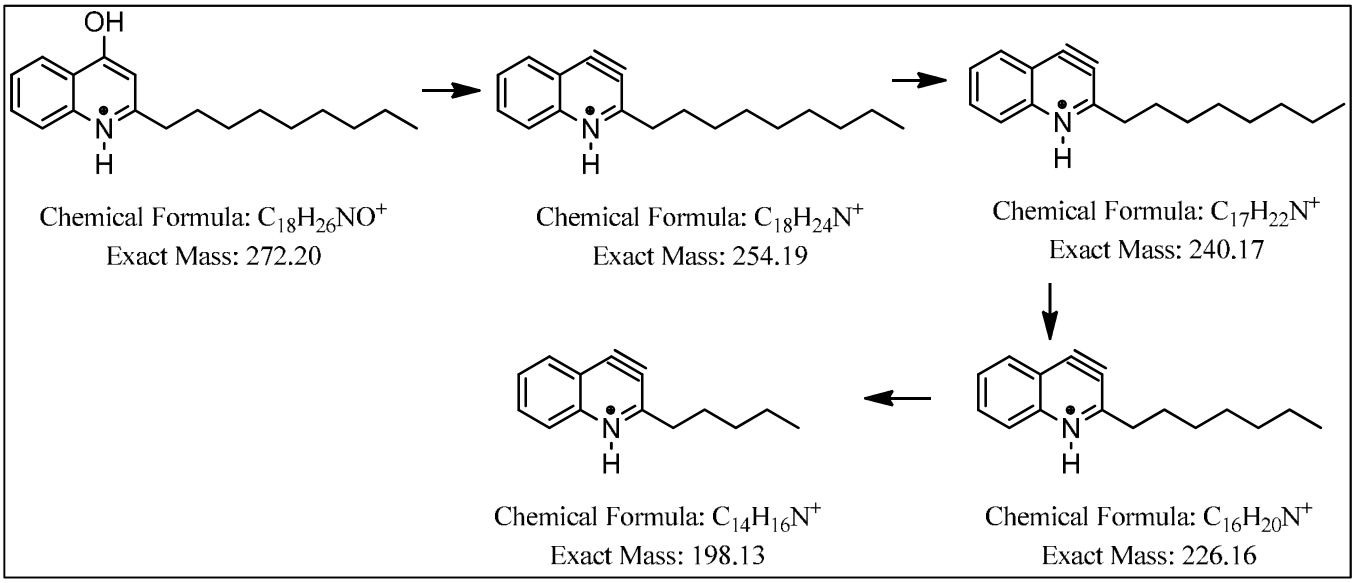
OH
N
H
Chemical Formula: C18H26NO+
Exact Mass: 272.20
Chemical Formula: C18H24N+
Exact Mass: 254.19
Chemical Formula: C17H22N+
Exact Mass: 240.17
Chemical Formula: C16H20N+
Exact Mass: 226.16
Chemical Formula: C14H16N+
Exact Mass: 198.13

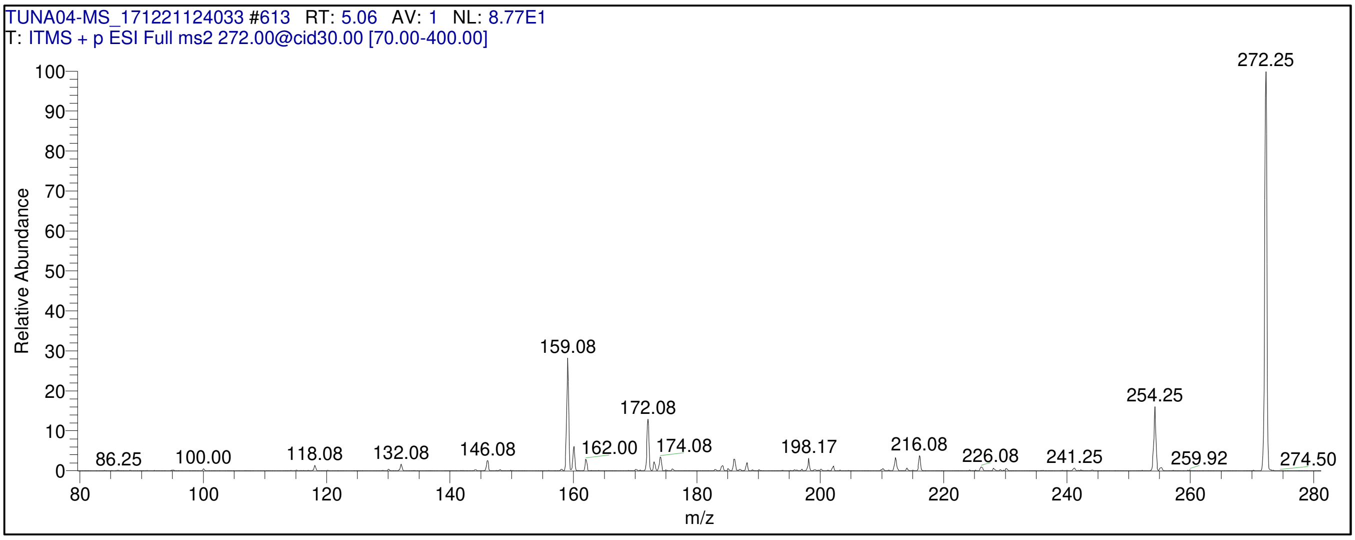
TUNA04-MS_171221124033 #613 RT: 5.06 AV: 1 NL: 8.77E1
T: ITMS + p ESI Full ms2 272.00@cid30.00 [70.00-400.00]
Relative Abundance
m/z
272.25
159.08
254.25
172.08
86.25
100.00
118.08
132.08
146.08
162.00
174.08
198.17
216.08
226.08
241.25
259.92
274.50

VI.

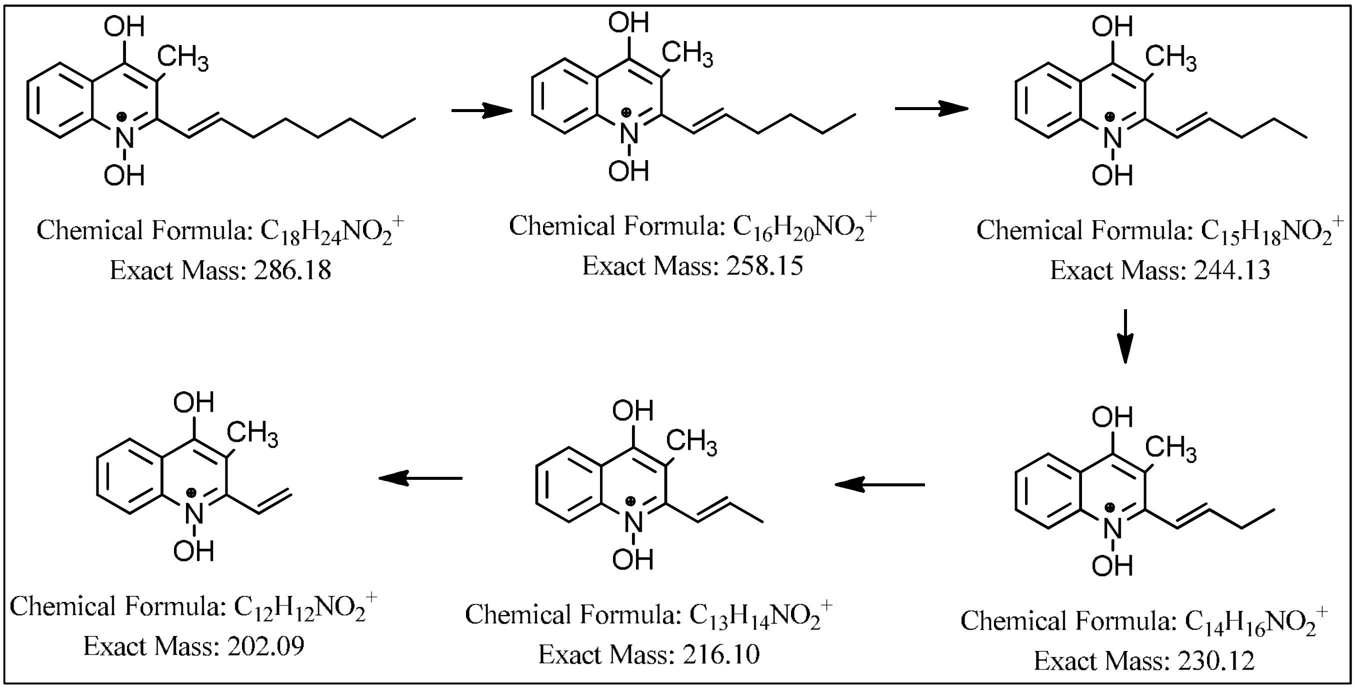
Chemical Formula: $C_{18}H_{24}NO_2^+$
Exact Mass: 286.18
Chemical Formula: $C_{16}H_{20}NO_2^+$
Exact Mass: 258.15
Chemical Formula: $C_{15}H_{18}NO_2^+$
Exact Mass: 244.13
Chemical Formula: $C_{14}H_{16}NO_2^+$
Exact Mass: 230.12
Chemical Formula: $C_{13}H_{14}NO_2^+$
Exact Mass: 216.10
Chemical Formula: $C_{12}H_{12}NO_2^+$
Exact Mass: 202.09

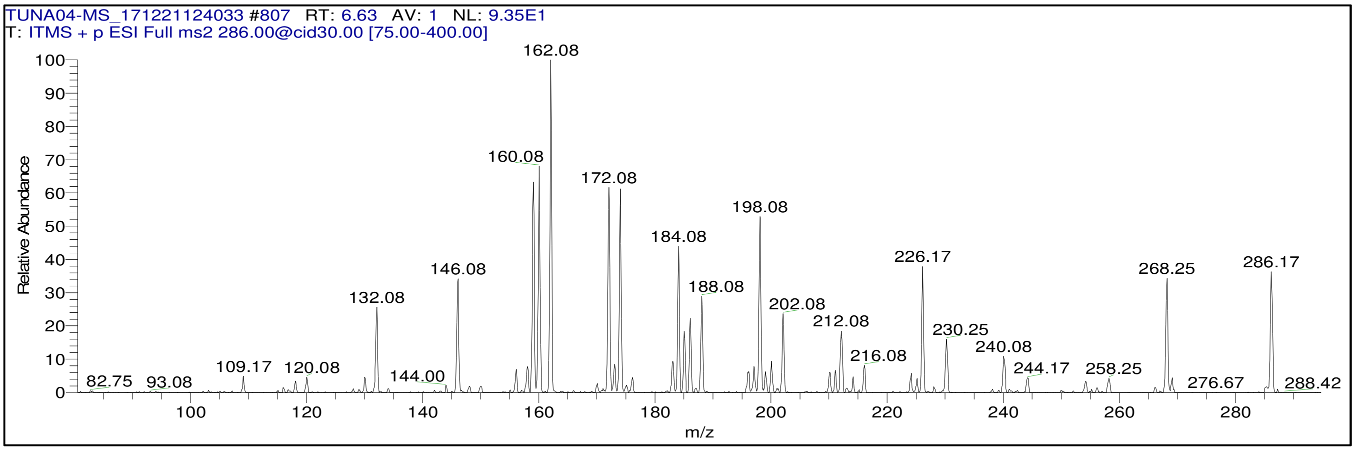
TUNA04-MS_171221124033 #807 RT: 6.63 AV: 1 NL: 9.35E1
T: ITMS + p ESI Full ms2 286.00@cid30.00 [75.00-400.00]
Relative Abundance
m/z
162.08
160.08
172.08
198.08
184.08
226.17
146.08
286.17
268.25
188.08
132.08
202.08
212.08
230.25
240.08
216.08
109.17
120.08
244.17
258.25
144.00
82.75
93.08
276.67
288.42

VII.

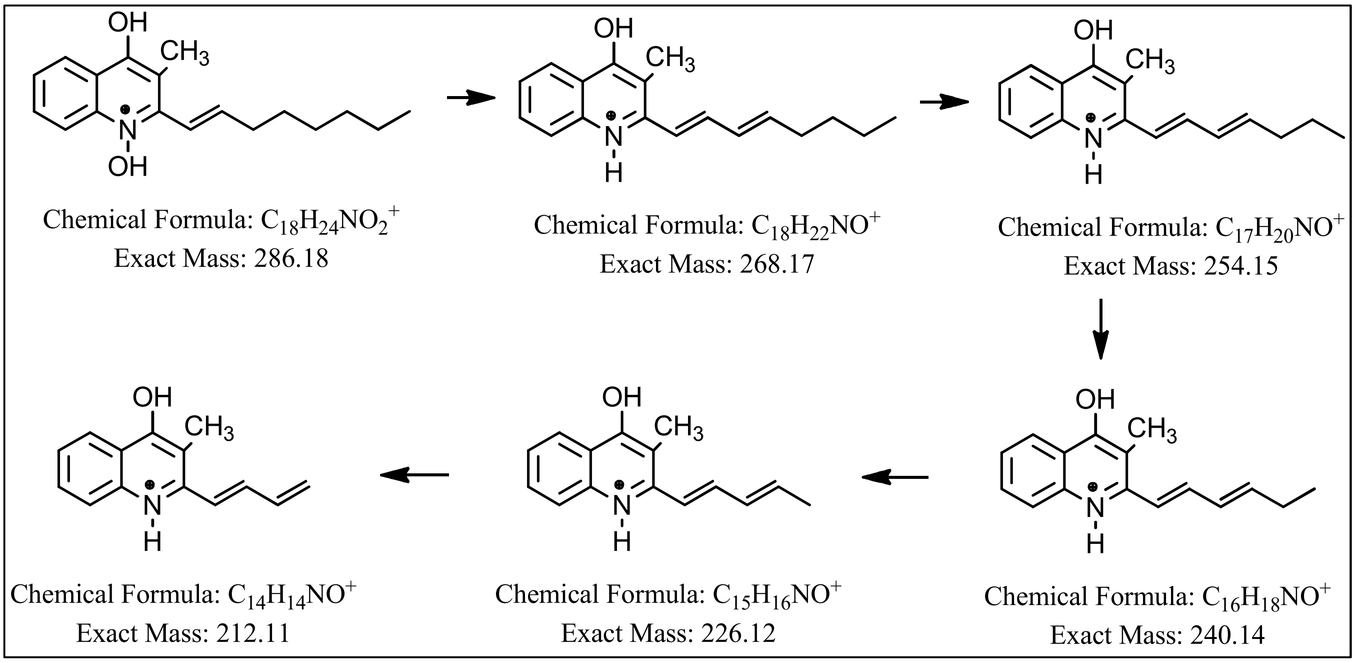

OH
CH3
N
OH
Chemical Formula: $C_{18}H_{24}NO_2^+$
Exact Mass: 286.18
H
Chemical Formula: $C_{18}H_{22}NO^+$
Exact Mass: 268.17
Chemical Formula: $C_{17}H_{20}NO^+$
Exact Mass: 254.15
Chemical Formula: $C_{14}H_{14}NO^+$
Exact Mass: 212.11
Chemical Formula: $C_{15}H_{16}NO^+$
Exact Mass: 226.12
Chemical Formula: $C_{16}H_{18}NO^+$
Exact Mass: 240.14

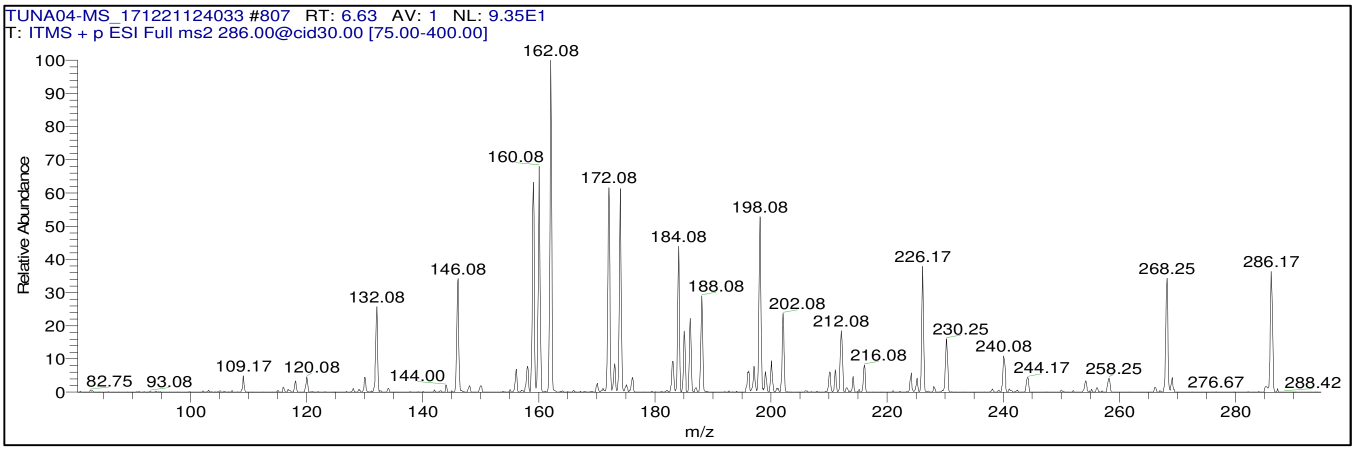

TUNA04-MS_171221124033 #807 RT: 6.63 AV: 1 NL: 9.35E1
T: ITMS + p ESI Full ms2 286.00@cid30.00 [75.00-400.00]
Relative Abundance
m/z
162.08
160.08
172.08
198.08
184.08
226.17
286.17
268.25
146.08
188.08
132.08
202.08
212.08
230.25
216.08
240.08
244.17
258.25
109.17
120.08
144.00
82.75
93.08
276.67
288.42

VIII

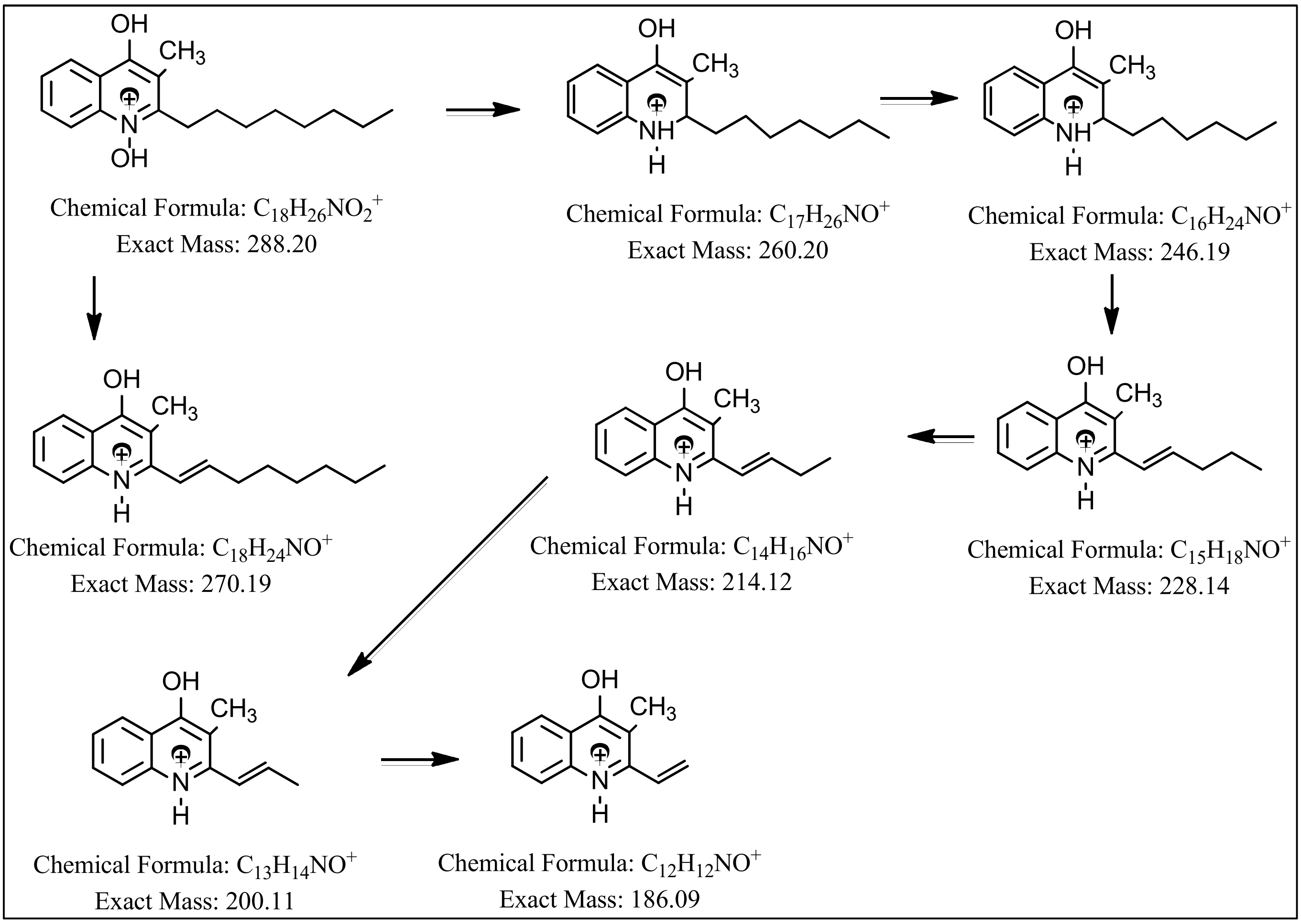
Chemical Formula: $C_{18}H_{26}NO_2^+$
Exact Mass: 288.20
Chemical Formula: $C_{17}H_{26}NO^+$
Exact Mass: 260.20
Chemical Formula: $C_{16}H_{24}NO^+$
Exact Mass: 246.19
Chemical Formula: $C_{18}H_{24}NO^+$
Exact Mass: 270.19
Chemical Formula: $C_{14}H_{16}NO^+$
Exact Mass: 214.12
Chemical Formula: $C_{15}H_{18}NO^+$
Exact Mass: 228.14
Chemical Formula: $C_{13}H_{14}NO^+$
Exact Mass: 200.11
Chemical Formula: $C_{12}H_{12}NO^+$
Exact Mass: 186.09

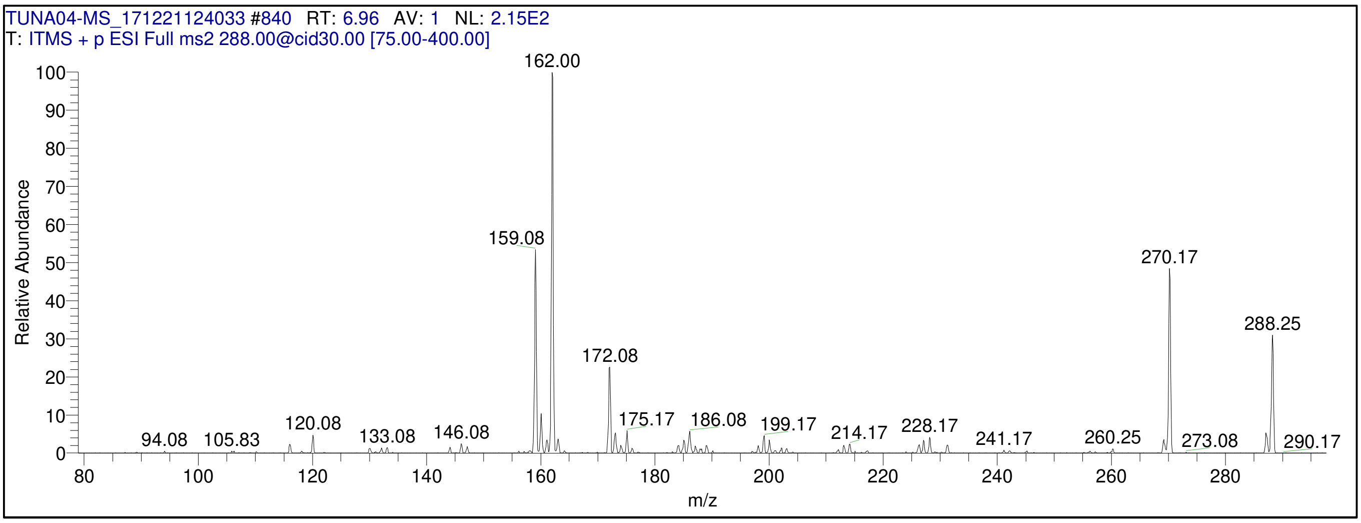
TUNA04-MS_171221124033 #840 RT: 6.96 AV: 1 NL: 2.15E2
T: ITMS + p ESI Full ms2 288.00@cid30.00 [75.00-400.00]
Relative Abundance
m/z
162.00
159.08
270.17
288.25
172.08
94.08
105.83
120.08
133.08
146.08
175.17
186.08
199.17
214.17
228.17
241.17
260.25
273.08
290.17

IX.

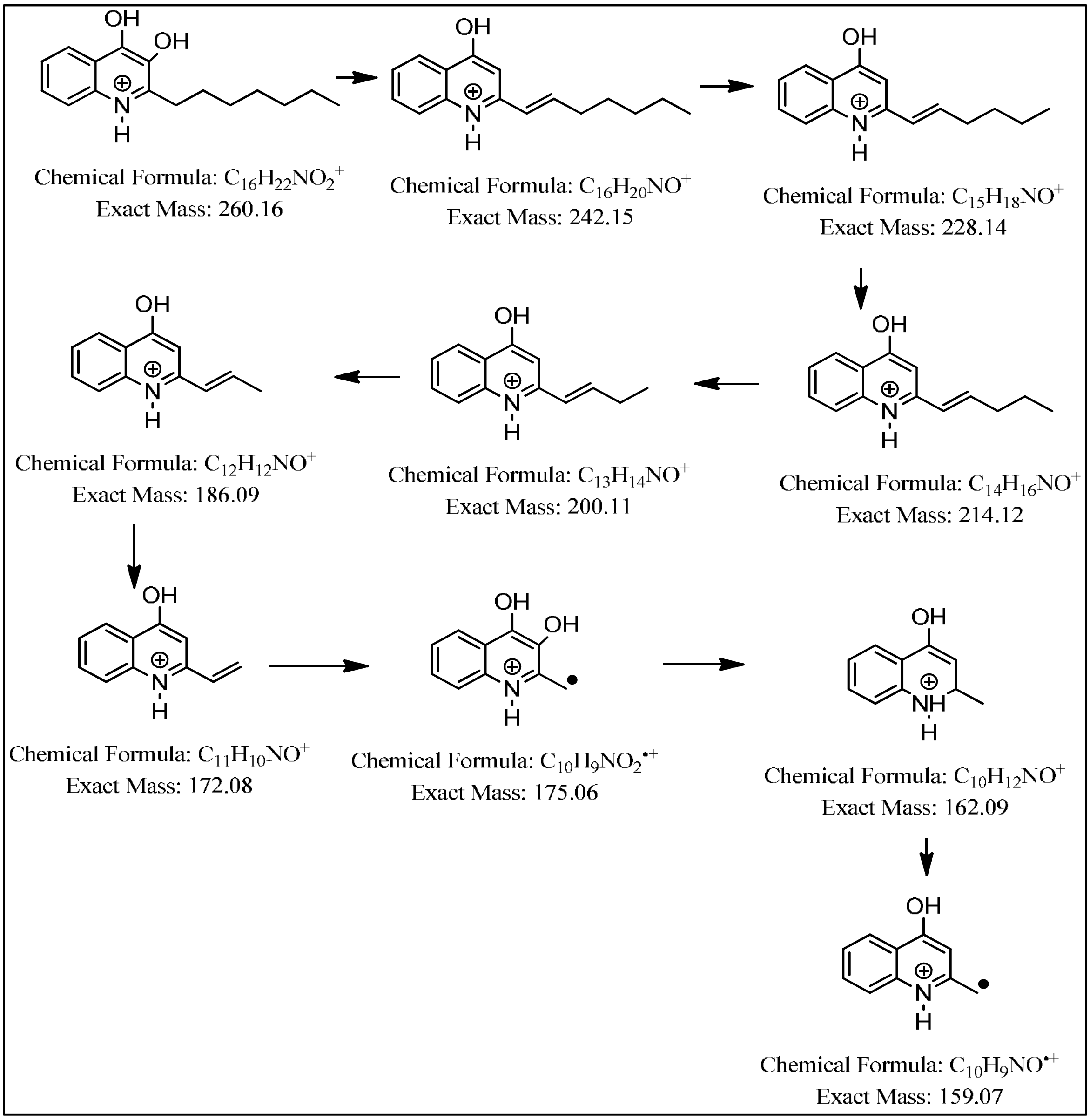
Chemical Formula: $C_{16}H_{22}NO_2^+$
Exact Mass: 260.16
Chemical Formula: $C_{16}H_{20}NO^+$
Exact Mass: 242.15
Chemical Formula: $C_{15}H_{18}NO^+$
Exact Mass: 228.14
Chemical Formula: $C_{12}H_{12}NO^+$
Exact Mass: 186.09
Chemical Formula: $C_{13}H_{14}NO^+$
Exact Mass: 200.11
Chemical Formula: $C_{14}H_{16}NO^+$
Exact Mass: 214.12
Chemical Formula: $C_{11}H_{10}NO^+$
Exact Mass: 172.08
Chemical Formula: $C_{10}H_9NO_2^{\bullet+}$
Exact Mass: 175.06
Chemical Formula: $C_{10}H_{12}NO^+$
Exact Mass: 162.09
Chemical Formula: $C_{10}H_9NO^{\bullet+}$
Exact Mass: 159.07

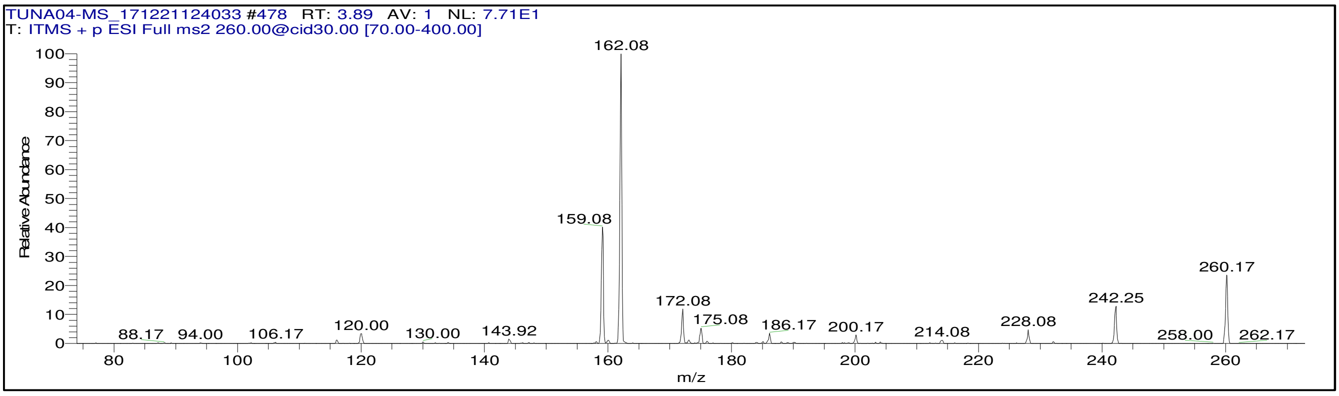
TUNA04-MS_171221124033 #478 RT: 3.89 AV: 1 NL: 7.71E1
T: ITMS + p ESI Full ms2 260.00@cid30.00 [70.00-400.00]
Relative Abundance
m/z
162.08
159.08
172.08
175.08
186.17
200.17
214.08
228.08
242.25
260.17
258.00
262.17
88.17
94.00
106.17
120.00
130.00
143.92

Fig. S3 Putative structures of the fragment ion produced by CID of the **I)** *m/z* 504, **II)** *m/z* 649 [M-H]$^-$, **III)** *m/z* 803 [M+H]$^+$ and **IV)** illustrative structures of the rhamnolipids molecules obtained through LC/MS having *m/z* 762, *m/z* 677, *m/z* 649, 503, 475 and 357 [M-H]$^-$.

I.

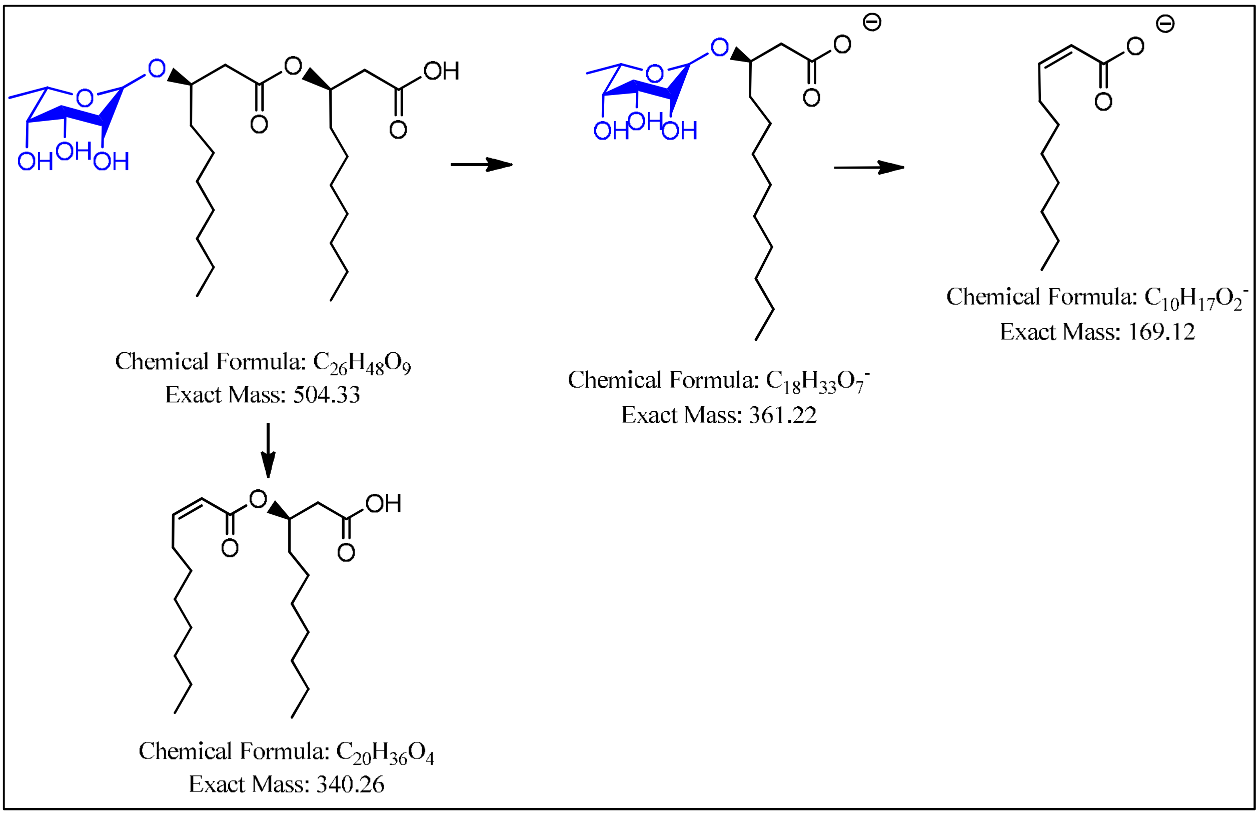

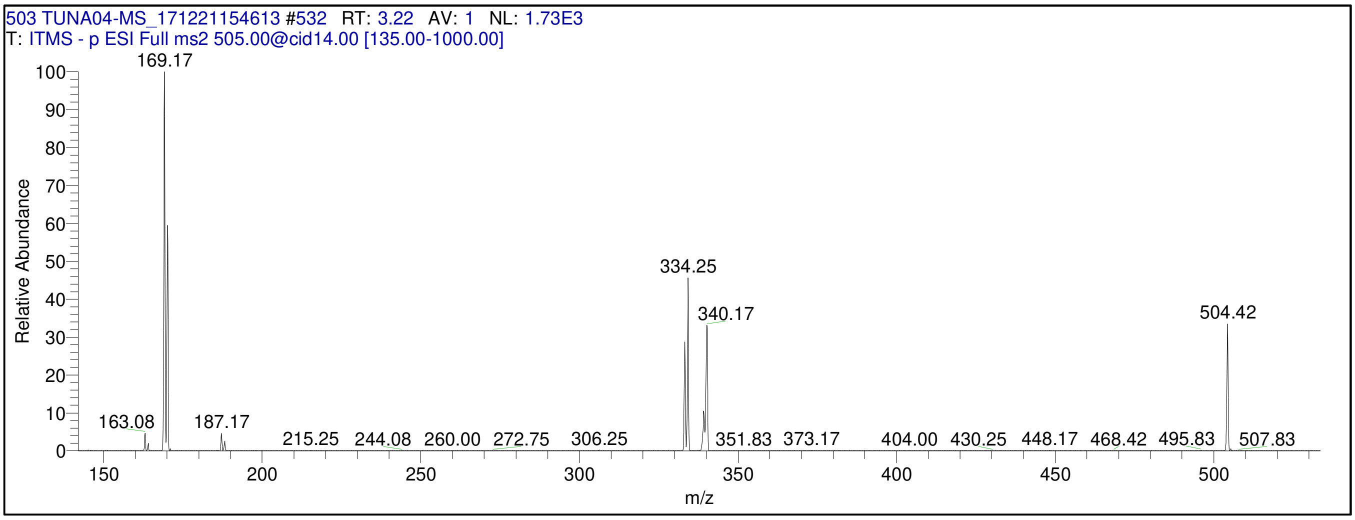

II.

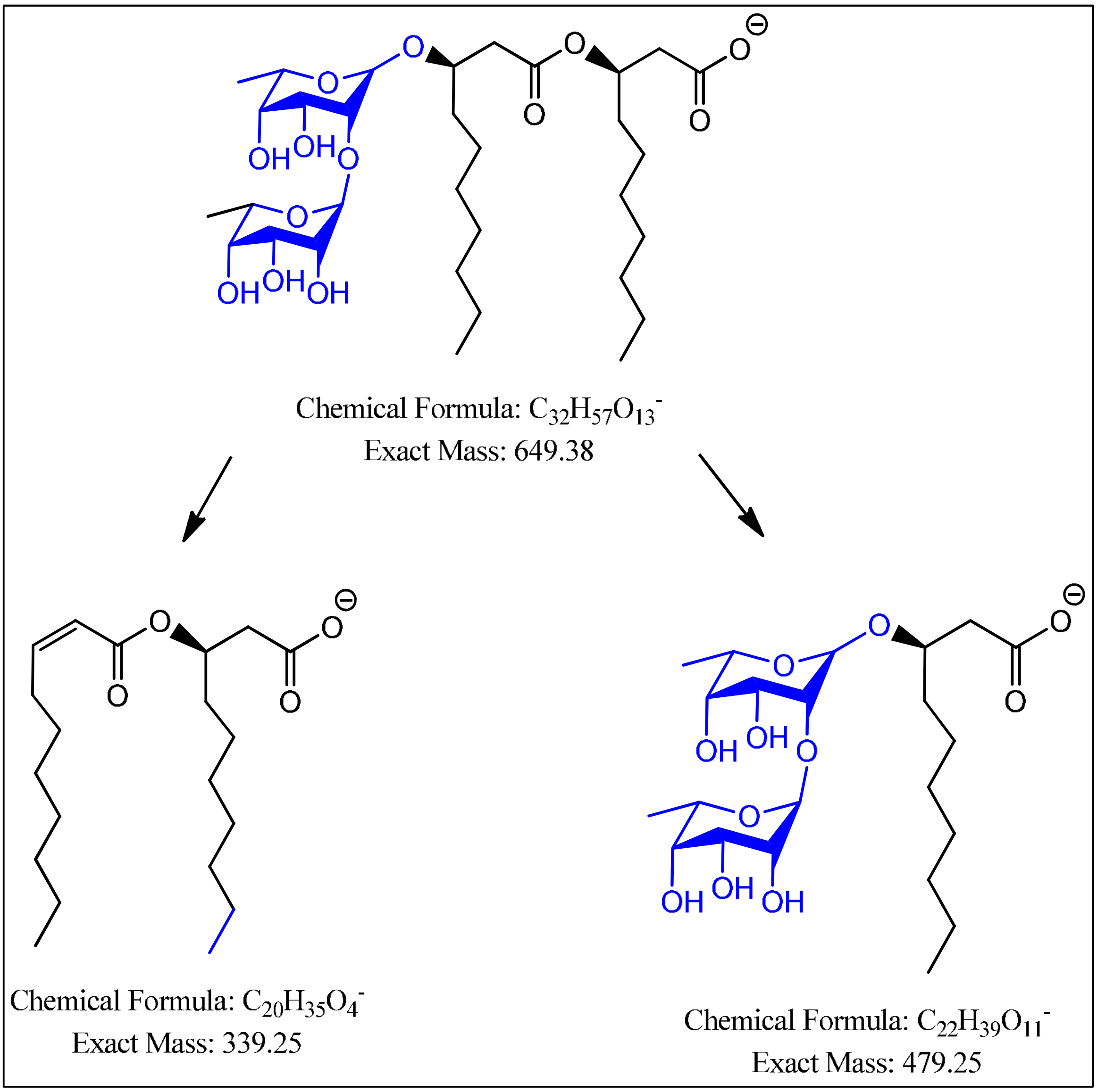
Chemical Formula: $C_{32}H_{57}O_{13}^{-}$
Exact Mass: 649.38
Chemical Formula: $C_{20}H_{35}O_{4}^{-}$
Exact Mass: 339.25
Chemical Formula: $C_{22}H_{39}O_{11}^{-}$
Exact Mass: 479.25

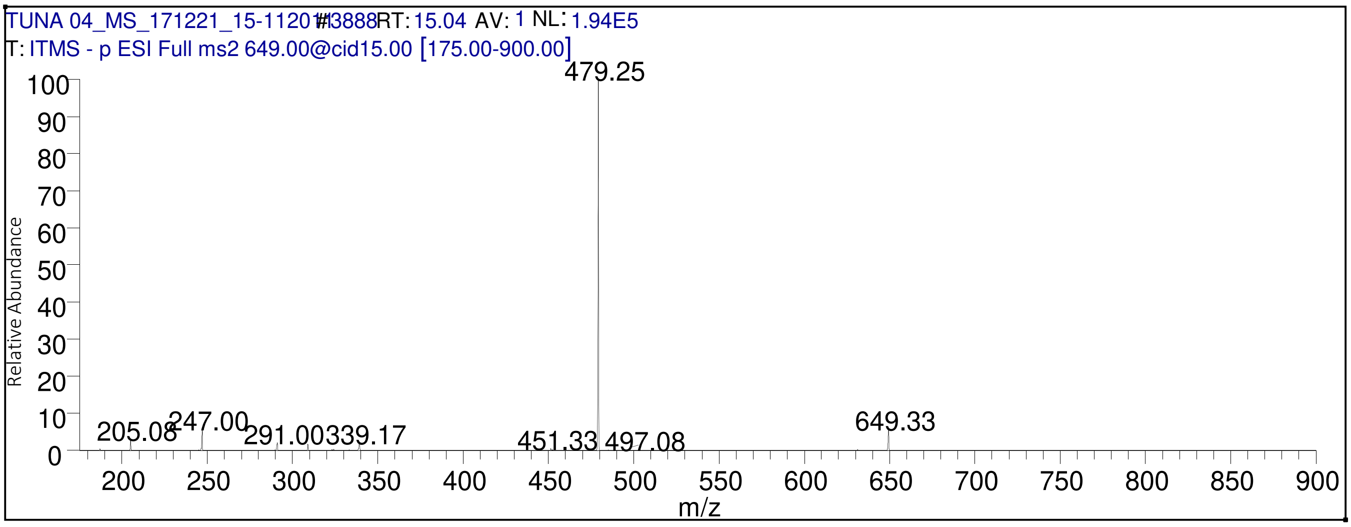
TUNA 04_MS_171221_15-11201#3888 RT: 15.04 AV: 1 NL: 1.94E5
T: ITMS - p ESI Full ms2 649.00@cid15.00 [175.00-900.00]
479.25
205.08
247.00
291.00
339.17
451.33
497.08
649.33
Relative Abundance
m/z

III.

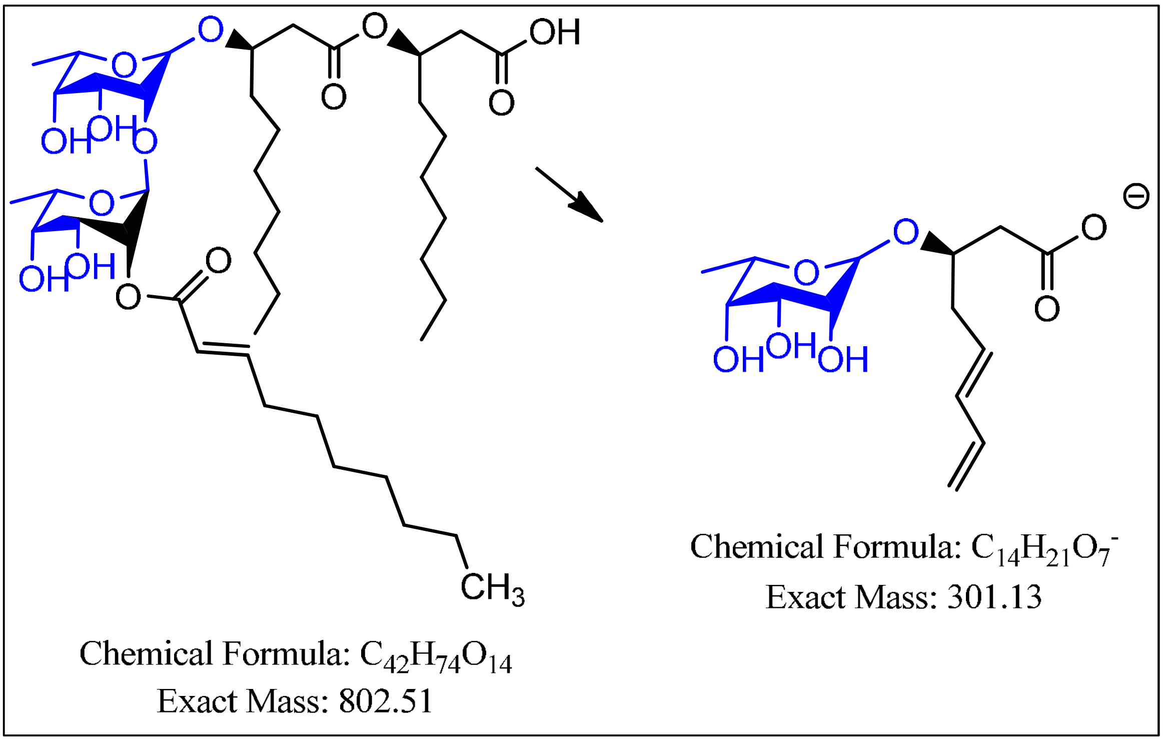

OH
Chemical Formula: C42H74O14
Exact Mass: 802.51
Chemical Formula: C14H21O7-
Exact Mass: 301.13

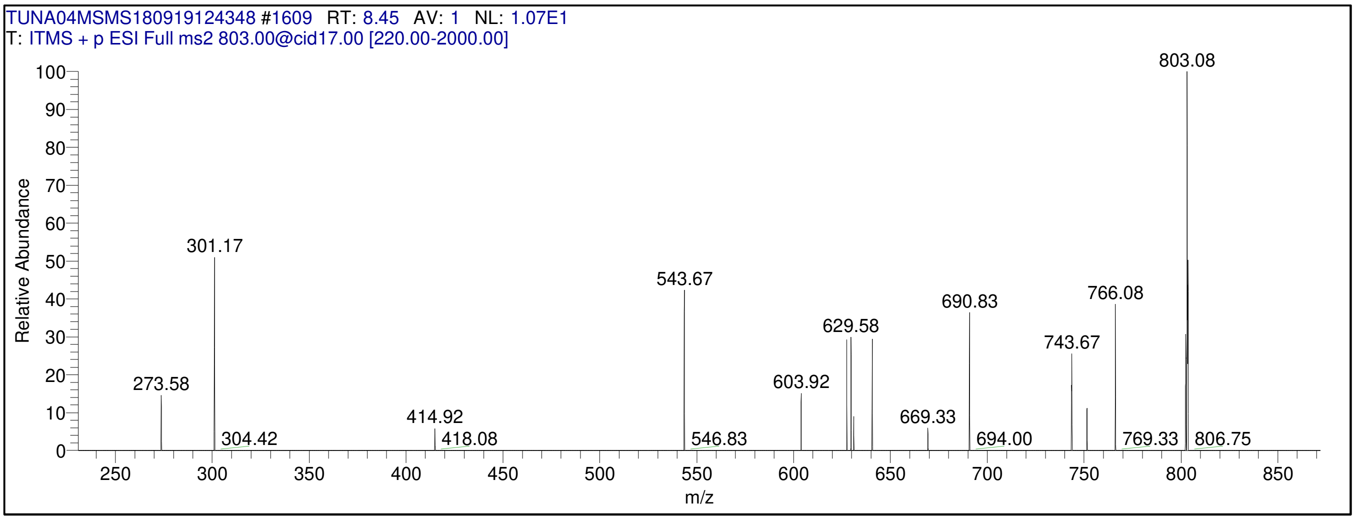

TUNA04MSMS180919124348 #1609 RT: 8.45 AV: 1 NL: 1.07E1
T: ITMS + p ESI Full ms2 803.00@cid17.00 [220.00-2000.00]
Relative Abundance
m/z
803.08
301.17
543.67
690.83
766.08
629.58
743.67
603.92
273.58
414.92
669.33
304.42
418.08
546.83
694.00
769.33
806.75

IV.

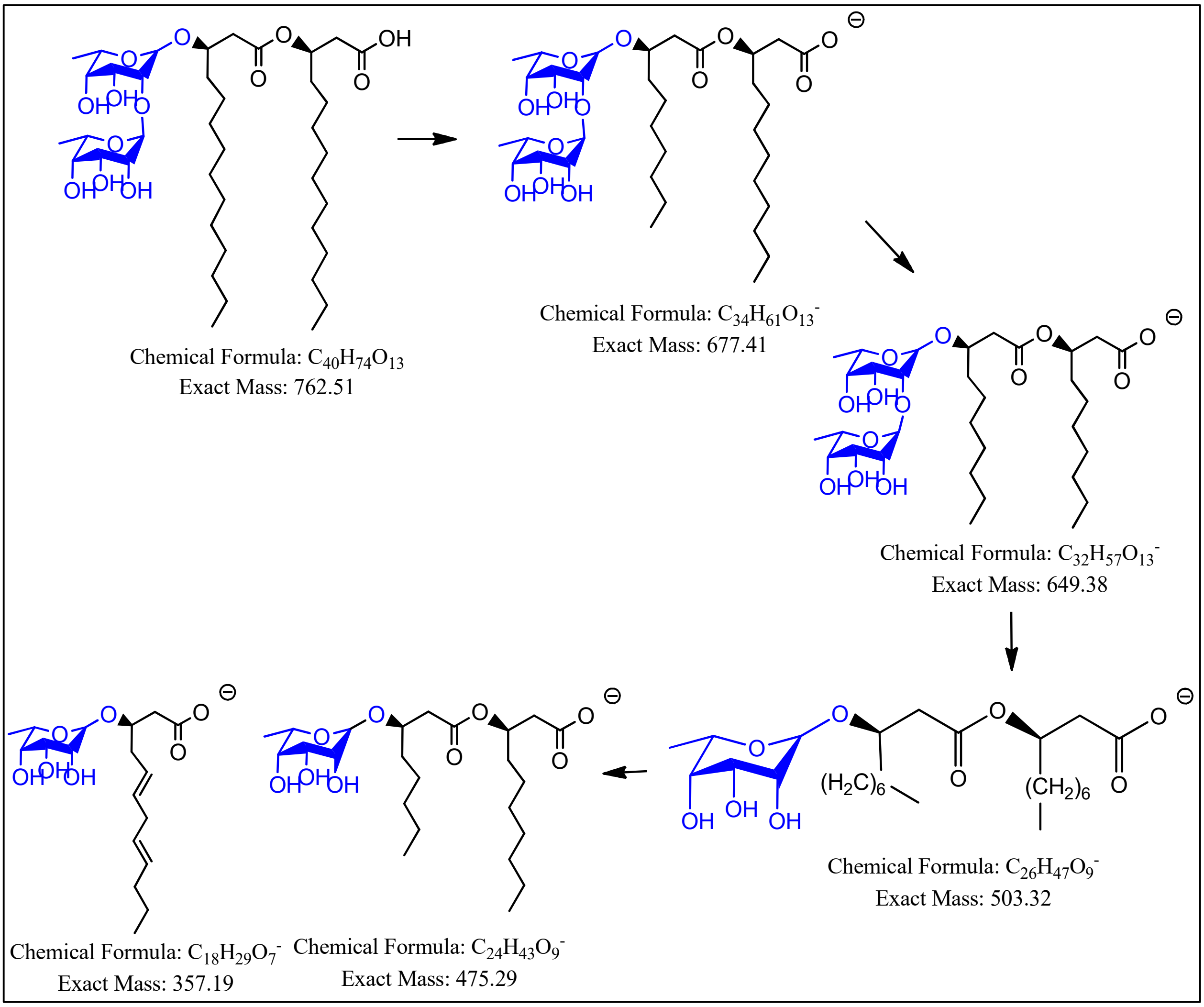

Chemical Formula: C40H74O13
Exact Mass: 762.51
Chemical Formula: C34H61O13-
Exact Mass: 677.41
Chemical Formula: C32H57O13-
Exact Mass: 649.38
Chemical Formula: C26H47O9-
Exact Mass: 503.32
Chemical Formula: C24H43O9-
Exact Mass: 475.29
Chemical Formula: C18H29O7-
Exact Mass: 357.19

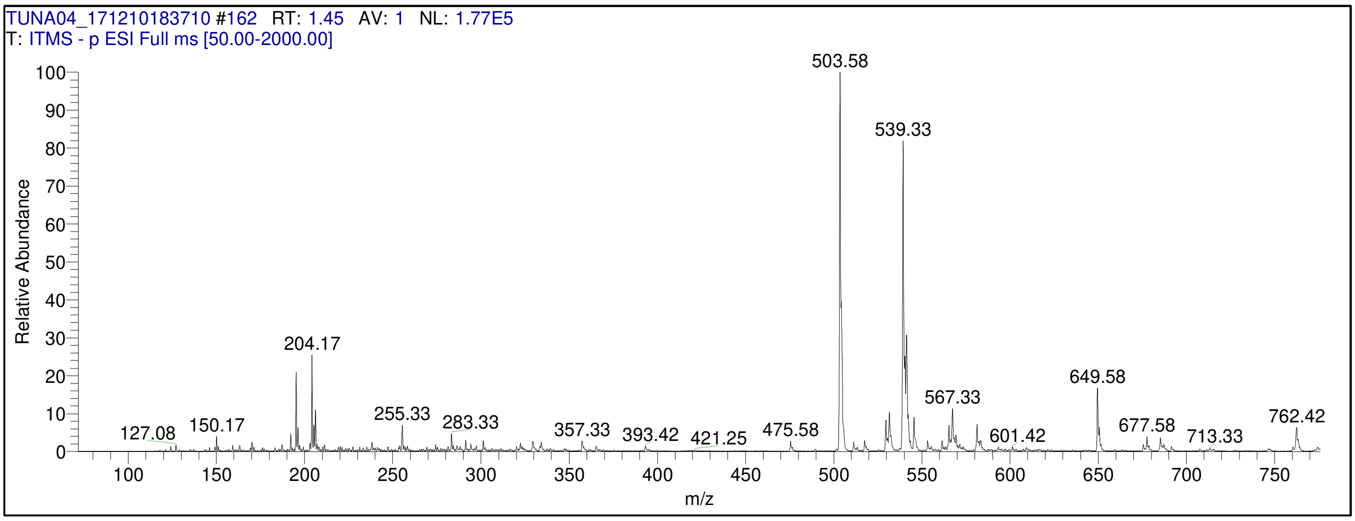

TUNA04_171210183710 #162 RT: 1.45 AV: 1 NL: 1.77E5
T: ITMS - p ESI Full ms [50.00-2000.00]
Relative Abundance
m/z
503.58
539.33
204.17
649.58
567.33
762.42
255.33
283.33
127.08
150.17
357.33
393.42
421.25
475.58
601.42
677.58
713.33

Table S4. Turtle gut bacterial 16S rRNA consensus gene sequences.

Sample/bacteria	Consensus sequences
>CM1_16S	TAGCACAGAGAGCTTGCTCTCGGGTGACGAGTGGCGGACGGGTGAGTAATGTCTGG<br>GAAACTGCCTGATGGAGGGGGATAACTACTGGAAACGGTAGCTAATACCGCATAAC<br>GTCGCAAGACCAAAGAGGGGGACCTTCGGGCCTCTTGCCATCAGATGTGCCCAGATG<br>GGATTAGCTAGTAGGTGGGGTAATGGCTCACCTAGGCGACGATCCCTAGCTGGTCTG<br>AGAGGATGACCAGCCACACTGGAACTGAGACACGGTCCAGACTCCTACGGGAGGCA<br>GCAGTGGGGAATATTGCACAATGGGCGCAAGCCTGATGCAGCCATGCCGCGTGTATG<br>AAGAAGGCCTTCGGGTTGTAAAGTACTTTCAGCGGGGAGGAAGGTGTTGTGGTTAAT<br>AACCGCAGCAATTGACGTTACCCGCAGAAGAAGCACCGGCTAACTCCGTGCCAGCAG<br>CCGCGGTAATACGGAGGGTGCAAGCGTTAATCGGAATTACTGGGCGTAAAGCGCAC<br>GCAGGCGGTCTGTCAAGTCGGATGTGAAATCCCCGGGCTCAACCTGGGAACTGCATT<br>CGAAACTGGCAGGCTAGAGTCTTGTAGAGGGGGGTAGAATTCCAGGTGTAGCGGTG<br>AAATGCGTAGAGATCTGGAGGAATACCGGTGGCGAAGGCGGCCCCCTGGACAAAGA<br>CTGACGCTCAGGTGCGAAAGCGTGGGGAGCAAACAGGATTAGATACCCTGGTAGTC<br>CACGCCGTAAACGATGTCGACTTGGAGGTTGTTCCCTTGAGGAGTGGCTTCCGGAGC<br>TAACGCGTTAAGTCGACCGCCTGGGGAGTACGGCCGCAAGGTTAAAACTCAAATGAA<br>TTGACGGGGGCCCGCACAAGCGGTGGAGCATGTGGTTTAATTCGATGCAACGCGAA<br>GAACCTTACCTACTCTTGACATCCAGAGAACTTAGCAGAGATGCTTTGGTGCCTTCGG<br>GAACTCTGAGACAGGTGCTGCATGGCTGTCGTCAGCTCGTGTTGTGAAATGTTGGGT<br>TAAGTCCCGCAACGAGCGCAACCCTTATCCTTTGTTGCCAGCGGTTCGGCCGGGAACT<br>CAAAGGAGACTGCCAGTGATAAACTGGAGGAAGGTGGGGATGACGTCAAGTCATCA<br>TGGCCCTTACGAGTAGGGCTACACACGTGCTACAATGGCGCATACAAAGAGAAGCGA<br>CCTCGCGAGAGCAAGCGGACCTCATAAAGTGCGTCGTAGTCCGGATTGGAGTCTGCA<br>ACTCGACTCCATGAAGTCGGAATCGCTAGTAATCGTAGATCAGAATGCTACGGTGAA<br>TACGTTCCCGGGCCTTGTACACACCGCCCGTCACACC
>CM2_16S	GCTACTTTTGCCGGCGAGCGGCGGACGGGTGAGTAATGCCTGGGAAATTGCCCAGTC<br>GAGGGGGATAACAGTTGGAAACGACTGCTAATACCGCATACGCCCTACGGGGGAAA<br>GCAGGGGACCTTCGGGCCTTGCGCGATTGGATATGCCCAGGTGGGATTAGCTAGTTG<br>GTGAGGTAATGGCTCACCAAGGCGACGATCCCTAGCTGGTCTGAGAGGATGATCAGC<br>CACACTGGAACTGAGACACGGTCCAGACTCCTACGGGAGGCAGCAGTGGGGAATAT<br>TGCACAATGGGGGAAACCCTGATGCAGCCATGCCGCGTGTGTGAAGAAGGCCTTCG<br>GGTTGTAAAGCACTTTCAGCGAGGAGGAAAGGTTGATGCCTAATACGTATCAACTGT<br>GACGTTACTCGCAGAAGAAGCACCGGCTAACTCCGTGCCAGCAGCCGCGGTAATACG<br>GAGGGTGCAAGCGTTAATCGGAATTACTGGGCGTAAAGCGCACGCAGGCGGTTGGA<br>TAAGTTAGATGTGAAAGCCCCGGGCTCAACCTGGGAATTGCATTTAAAACTGTCCAG<br>CTAGAGTCTTGTAGAGGGGGGTAGAATTCCAGGTGTAGCGGTGAAATGCGTAGAGA<br>TCTGGAGGAATACCGGTGGCGAAGGCGGCCCCCTGGACAAAGACTGACGCTCAGGT<br>GCGAAAGCGTGGGGAGCAAACAGGATTAGATACCCTGGTAGTCCACGCCGTAAACG<br>ATGTCGATTTGGAGGCTGTGTCCTTGAGACGTGGCTTCCGGAGCTAACGCGTTAAATC<br>GACCGCCTGGGGAGTACGGCCGCAAGGTTAAAACTCAAATGAATTGACGGGGGCCC<br>GCACAAGCGGTGGAGCATGTGGTTTAATTCGATGCAACGCGAAGAACCTTACCTGGC<br>CTTGACATGTCTGGAATCCTGTAGAGATACGGGAGTGCCTTCGGGAATCAGAACACA<br>GGTGCTGCATGGCTGTCGTCAGCTCGTGTCGTGAGATGTTGGGTTAAGTCCCGCAAC<br>GAGCGCAACCCCTGTCCTTTGTTGCCAGCACGTAATGGTGGGAACTCAAGGGAGACT<br>GCCGGTGATAAACCGGAGGAAGGTGGGGATGACGTCAAGTCATCATGGCCCTTACG<br>GCCAGGGCTACACACGTGCTACAATGGCGCGTACAGAGGGCTGCAAGCTAGCGATA<br>GTGAGCGAATCCCAAAAAGCGCGTCGTAGTCCGGATCGGAGTCTGCAACTCGACTCC<br>GTGAAGTCGGAATCGCTAGTAATCGCAAATCAGAATGTTGCGGTGAATACGTTCCCG<br>GGCCTTGTACACACCGCCCGTCACACCATGGGAGTGGGTTGCACCAG
